# Resolving Discrepancy between Nucleotides and Amino Acids in Deep-Level Arthropod Phylogenomics: Differentiating Serine Codons in 21-Amino-Acid Models

**DOI:** 10.1371/journal.pone.0047450

**Published:** 2012-11-20

**Authors:** Andreas Zwick, Jerome C. Regier, Derrick J. Zwickl

**Affiliations:** 1 Department of Entomology, State Museum of Natural History, Stuttgart, Germany; 2 Institute for Bioscience and Biotechnology Research and Department of Entomology, University of Maryland, College Park, Maryland, United States of America; 3 Department of Ecology and Evolutionary Biology, University of Kansas, Lawrence, Kansas, United States of America; Midwestern University, United States of America

## Abstract

**Background:**

In a previous study of higher-level arthropod phylogeny, analyses of nucleotide sequences from 62 protein-coding nuclear genes for 80 panarthopod species yielded significantly higher bootstrap support for selected nodes than did amino acids. This study investigates the cause of that discrepancy.

**Methodology/Principal Findings:**

The hypothesis is tested that failure to distinguish the serine residues encoded by two disjunct clusters of codons (TCN, AGY) in amino acid analyses leads to this discrepancy. In one test, the two clusters of serine codons (*Ser1*, *Ser2*) are conceptually translated as separate amino acids. Analysis of the resulting 21-amino-acid data matrix shows striking increases in bootstrap support, in some cases matching that in nucleotide analyses. In a second approach, nucleotide and 20-amino-acid data sets are artificially altered through targeted deletions, modifications, and replacements, revealing the pivotal contributions of distinct *Ser1* and *Ser2* codons. We confirm that previous methods of coding nonsynonymous nucleotide change are robust and computationally efficient by introducing two new degeneracy coding methods. We demonstrate for degeneracy coding that neither compositional heterogeneity at the level of nucleotides nor codon usage bias between *Ser1* and *Ser2* clusters of codons (or their separately coded amino acids) is a major source of non-phylogenetic signal.

**Conclusions:**

The incongruity in support between amino-acid and nucleotide analyses of the forementioned arthropod data set is resolved by showing that “standard” 20-amino-acid analyses yield lower node support specifically when serine provides crucial signal. Separate coding of *Ser1* and *Ser2* residues yields support commensurate with that found by degenerated nucleotides, without introducing phylogenetic artifacts. While exclusion of *all* serine data leads to reduced support for serine-sensitive nodes, these nodes are still recovered in the ML topology, indicating that the enhanced signal from *Ser1* and *Ser2* is not qualitatively different from that of the other amino acids.

## Introduction

With the advent of next generation sequencing techniques, the number of available expressed sequence tag libraries and entire transcriptomes has grown at an unprecedented pace. These techniques have fostered a surge in the number of studies that interpret data in a phylogenetic framework, and even tackle reconstruction of the tree of life. The majority of deep-level phylogenies relies on amino acid alignment and analysis, despite nucleotides being the primary sequence data. This reflects the common notion that rapidly evolving synonymous nucleotide changes are mostly uninformative at this level, yet often cause analytical problems like long branch attraction and model violations, e.g., nucleotide compositional heterogeneity [1]–[4]. While the conceptual translation to amino acids eliminates these potentially problematic synonymous nucleotide changes, the modeling of changes across 20 amino acids is more complex and computationally demanding than the one across four nucleotide states. As a result, the choice of analytical method will de facto be increasingly limited with ever increasing phylogenomic data set sizes. Nonsynonymous-only coding schemes for nucleotides [5], [6] are an alternative and outperform current amino acid analyses computationally. For example, *degen1* coding, in which all codons that encode the same amino acid are fully degenerated (described in Materials and Methods, [5]), is compatible with all major analysis packages and greatly reduces computational demands as compared to amino acid and codon model analyses (3–10 times less RAM and 2–60 times faster, respectively, in the case of [5]).

However, a recently published case study of relationships within arthropods [5] shows striking quantitative differences in support between amino acid and nucleotide coding methods for several key nodes, which raises uncertainties about the accuracy of both methods. In that report (see also [7]–[11]), we dealt with the challenge of nucleotide compositional heterogeneity by analyzing the data with a traditional amino acid model (JTT; [12]), a codon model and a standard nucleotide model using variously coded nucleotide data, including *degen1* degeneracy coding. Analytical results were well supported and broadly consistent across these different approaches, except that amino acid results had greatly decreased support for six of the nodes, all of which happen to be “deep” and of much current interest (cf., *20AA* and *degen1* values for nodes identified by filled circles in Figure 1 of [5]). For these six nodes but not the others (Figure S1, Table S1), bootstrap support provided by amino acids is reduced (−55% points on average) relative to that of nonsynonymous nucleotides. This is unexpected, since both types of characters are based on the same principle of capturing amino acid change.

**Figure 1:**
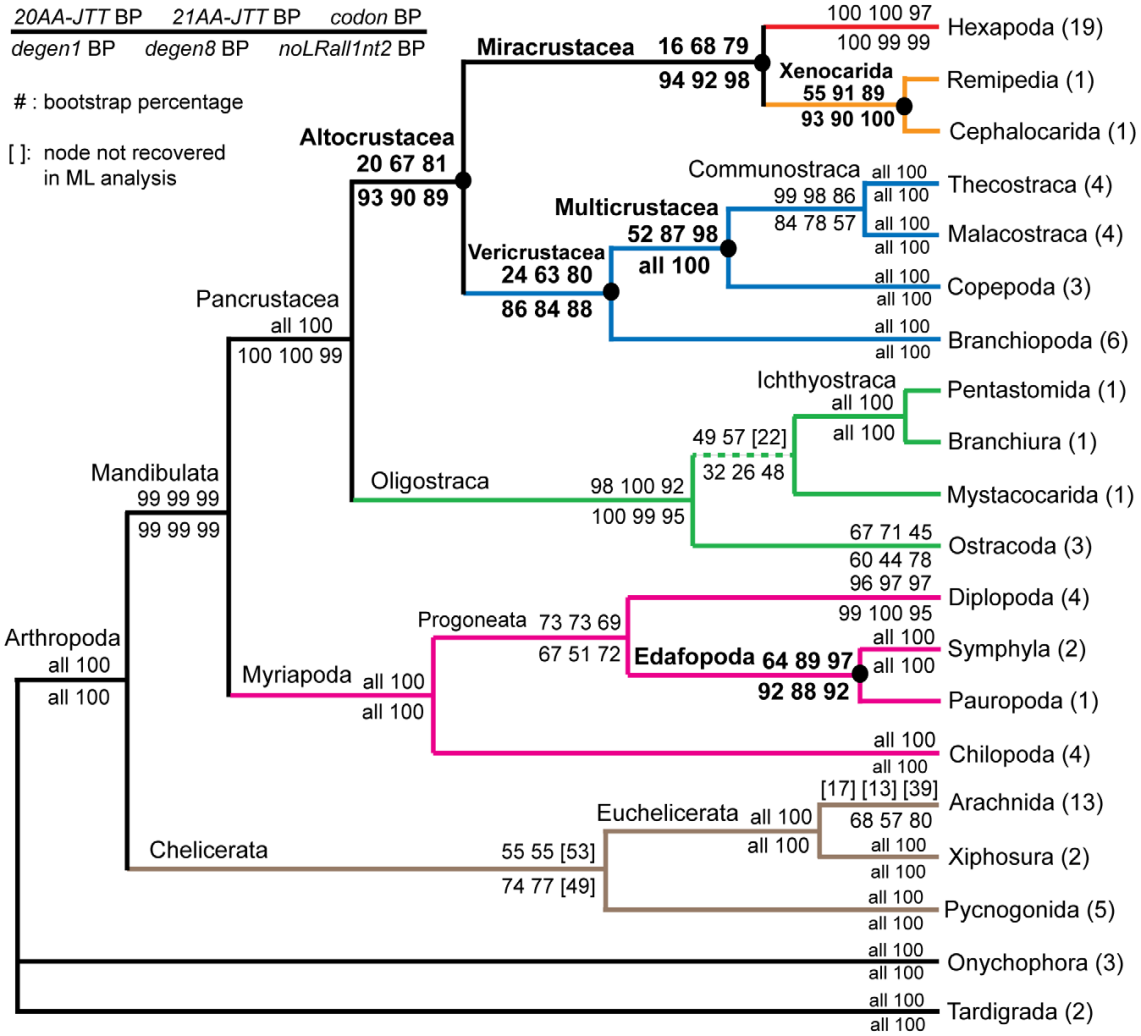
Deep-level arthropod relationships based on six analytical approaches. Aligned sequences from 75 arthropods and five outgroup species for 62 nuclear protein-coding genes were analyzed under the likelihood criterion using six strategies: *20AA-JTT*, a 20-amino-acid JTT model [12]; *21AA-JTT*, a 21-amino-acid JTT model; *codon*, a codon model; *degen1*; *degen8; noLRall1nt2*. These strategies are described in the Data Set Encoding section of Materials and Methods and in [5], [6]. Numbers of species representing terminal taxa are in parentheses. Bootstrap percentages (BP) are on internal branches (*20AA, 21AA, codon, degen1, degen8,* and *noLRall1nt2;* see figure key for order). Six nodes with a major increase in their bootstrap support from *20AA JTT* to *21AA JTT* are identified with filled circles. A more complete listing of results can be found in Table S1.

One obvious difference between *degen1*-encoded nucleotide and amino acid analyses is that all current amino acid models fail to distinguish two classes of serine residues that are encoded by disjunct and non-adjacent clusters of codons – TCN (*Ser1*) and ACY (*Ser2*) [13], [14]. Indeed, this failure to distinguish *Ser1* and *Ser2* in standard 20-amino-acid models represents a loss of potentially useful, phylogenetic information and might be considered less optimal modeling ([13], but see [14], [15]), since substitutions between them are otherwise invisible and the substitution rates between the two *Ser* and other amino acids are are almost certainly unequal (see Empirical Codon Rate matrix of [16]). So, the biological rationale for a focus on serine in this report is a consequence of the organization of its codons. At the nucleotide level, *Ser1* and *Ser2* interconversion requires either a non-*Ser* intermediate (for single-nucleotide substitutions) or else a (near-) simultaneous double mutation. Either way provides a reason to suspect that their rates of inter-conversion are reduced relative to standard synonymous change. Like *Ser1* and *Ser2*, *Leu* and *Arg* are also each encoded by six codons, but unlike *Ser1* and *Ser2*, their codons are clustered together on the codon table and can undergo single-nucleotide-based interconversion without passaging another amino acid, just like all of the other non-*Ser* amino acids encoded by multiple codons. In this report the potential utility of distinguishing *Ser1* and *Ser2* is empirically tested for arthropod phylogenomics through implementation of new 21-amino-acid models. Furthermore, by introducing additional new methods for degeneracy coding of nucleotides, we show that degeneracy methods generally are robust to a variety of assumptions, arguing that the original nucleotide results [5] remain credible and that the observed discrepancy in support values results from a problem with the amino acid analyses.

## Results and Discussion

### Analysis of 21-amino-acid data matrices

As there was no software available to analyze under likelihood a 21-amino-acid matrix, that is, one that separately encodes *Ser1* and *Ser2*, we initially performed a simple parsimony analysis (Figure S2). The result reinforces the utility of distinguishing *Ser1* and *Ser2*, in that bootstrap values for the six nodes of interest increase with a 21-amino-acid translation, although overall bootstrap support across the tree is, not surprisingly, much reduced relative to the likelihood analysis.

To directly compare likelihood analysis results, the software GARLI [17] was modified to accommodate 21 amino acids with the GTR model, which estimates a rate matrix from the given data. Complementarily, we also constructed a fixed rate model from the published rate matrix of a codon model [16]. Because the standard JTT model had already been used for the 20-amino-acid arthropod data set [5], we also expanded the JTT rate matrix in GARLI to 21 amino acids by extrapolating rate factors from the results of the published codon-model rate matrix [16] for *Ser1* and *Ser2* (Table S2). Under these three 21-amino-acid models, bootstrap values for all six nodes increase strongly – by an average of 35% points (Table 1) – with minimal changes in the other nodes (average of 0.5% points; Table S1). Three of the six nodes now have bootstrap values >85%, comparable to the nonsynonymous nucleotide results. The other three nodes display increases of on average 40% points. We interpret this increased bootstrap support to indicate that distinguishing *Ser1* from *Ser2* in the 21-amino-acid models 1) provides additional signal and/or 2) leads to reduced character conflict. While high bootstrap values *per se* are not indicative of accuracy, congruence in ML topologies across distinct analyses, in particular, those that code all serines as *Ser1+2* versus those than code serines separately as either *Ser1* or as *Ser2*, indicates that the increased bootstrap values that result from separately coding *Ser1* and *Ser2* represent overall stronger phylogenetic signals (see below).

**Table 1:** Comparison of bootstrap percentages of phylogenetic estimates and data set manipulations for selected taxonomic groups.^a^

		Edafopoda	Altocrust-acea	Vericrust-acea	Multicrust-acea	Miracrust-acea	Xenocarida
**phylogenetic estimates**							
GTR+I+G	degen1	92	93	86	100	94	93
JTT	20AA	64	20	24	52	16	55
	21AA	89	66	64	90	66	87
	Δ	**+25**	**+46**	**+40**	**+38**	**+50**	**+32**
GTR	20AA	59	16	13	37	10	39
	21AA	80	58	40	74	45	70
	Δ	**+21**	**+41**	**+27**	**+37**	**+35**	**+31**
ECM	20AA	70	6	15	43	8	27
	21AA	87	52	54	86	42	64
	Δ	**+17**	**+46**	**+39**	**+43**	**+34**	**36**
**mimicking amino acids with nucleotides**							
Ser1 to Ser2	degen1	54	57	40	84	42	59
	Δ	**−38**	**−36**	**−46**	**−16**	**−52**	**−34**
Ser2 to Ser1	degen1	66	63	47	90	41	58
	Δ	**−26**	**−30**	**−39**	**−10**	**−53**	**−35**
**identifying important serines**							
no Ser1, no Ser2	degen1	59	32	26	53	27	52
	Δ	**−33**	**−61**	**−60**	**−47**	**−67**	**−41**
no co-Ser1, no co-Ser2	degen1	52	51	47	75	38	68
	Δ	**−40**	**−42**	**−39**	**−25**	**−56**	**−25**
no co-Ser1	degen1	60	55	41	75	42	69
	Δ	**−32**	**−38**	**−45**	**−25**	**−52**	**−24**
no co-Ser2	degen1	59	52	53	92	33	61
	Δ	**−33**	**−41**	**−33**	**−8**	**−61**	**−32**
no non-co-Ser1, no non-co-Ser2	degen1	94	88	78	100	91	91
	Δ	+2	−5	−8	0	−3	−2
no non-co-Ser1	degen1	94	90	82	100	93	92
	Δ	+2	−3	−4	0	−1	−1
no non-co-Ser2	degen1	92	90	84	100	93	92
	Δ	0	−3	−2	0	−1	−1
**separating serine codon clusters artificially in amino acid data**							
co-Ser1 to Phe	20AA	94	59	59	89	65	84
	Δ	**+29**	**+39**	**+35**	**+37**	**+49**	**+29**
co-Ser1 to Trp	20AA	95	69	66	97	64	84
	Δ	**+30**	**+49**	**+42**	**+45**	**+48**	**+29**
co-Ser1 to Tyr	20AA	95	65	70	95	67	83
	Δ	**+31**	**+45**	**+46**	**+43**	**+51**	**+28**
co-Ser2 to Phe	20AA	94	52	56	97	78	91
	Δ	**+30**	**+33**	**+32**	**+45**	**+62**	**+36**
co-Ser2 to Trp	20AA	95	67	69	98	80	92
	Δ	**+31**	**+47**	**+46**	**+46**	**+64**	**+37**
co-Ser2 to Tyr	20AA	93	62	62	97	73	90
	Δ	**+29**	**+42**	**+38**	**+45**	**+57**	**+35**

a Maximum likelihood bootstrap results of *phylogenetic estimates* ( = uppermost cluster of bootstrap values) for all nodes are in Table S1. Bootstrap results of manipulated matrices (*mimicking amino acids with nucleotides, indentifying important serines, separating serine codon clusters artificially in amino acid data*) are in Table S2. *GTR+I+G degen1*: Empirical nucleotide rate matrix estimated from the degen1-encoded data matrix according to a general time reversible model with rate heterogeneity estimated by a gamma function plus invariant sites. *JTT 21AA & 20AA*: Amino acid rate matrix of Jones, Taylor, and Thornton [12] with or without separate rate estimates for Ser1 and Ser2 (21AA and 20AA, respectively). *GTR 20AA & 21AA*: Empirical amino acid rate matrix estimated from the actual data according to a general time reversible model. *ECM 20AA & 21AA*: Amino acid rate matrix estimated from the PANDIT-based Empirical Codon Model [16]. Δ: For amino acid analyses, the difference in bootstrap percentages of the 21AA result minus the 20AA result. For nucleotide analyses, the difference in bootstrap percentages of the altered data set minus the unaltered data set (GTR+I+G). Differences >15% are bold. *Ser1 to Ser2*, *Ser2 to Ser1*: Ser1 codons (TCN) were artificially changed to Ser2 (AGY) from the degen1 data matrix, or vice versa. *No Ser1, no Ser2*: Ser1 and Ser2 codons were deleted (changed to NNN) from the degen1 data matrix. *No (non-)co-Ser1, no (non-)co-Ser2*: (Non-)ComminglingSer1 and Ser2 codons were both separately and together deleted from the degen1 data matrix. *Co-Ser1 (Ser2) to Phe, Trp, Tyr*: *Co-Ser1 (Ser2)* residues were changed to Phe, Trp, or Try residues, respectively.

### Artificial manipulation of data matrices, particularly serine codons and residues

The importance of serine for the given data set can be independently demonstrated by directly and artificially manipulating the nonsynonymous nucleotide data matrices (Table 1). Eliminating all and only *Ser1* and *Ser2* codons from the nucleotide matrix (*no Ser1, no Ser2*) leads to a major reduction in support (on average −51% points) of all six nodes in likelihood analyses, directly demonstrating the important contribution of serine. Support levels for other nodes that are strongly supported in the nucleotide analysis (typically with bootstraps of 100%) are largely unaffected (on average −1.9% points) in these serine deletion experiments, consistent with a surfeit of support from non-serine codons (Table S3). Likewise, if *Ser1* codons are converted to *Ser2* codons (*Ser1 to Ser2*), or vice versa (*Ser2 to Ser1*), thereby mimicking the conflation of *Ser1* and *Ser2* in the standard 20 amino acid matrix, bootstrap support for all six nodes decreases on average -35% points in nucleotide analyses to levels typical of the 20-amino-acid results.

Depending on the particular character (site) in the sequence alignments, *Ser1* and *Ser2* codons can either commingle (*“co-Ser1, co-Ser2”*) or not (*non-co-Ser1, non-co-Ser2*; see Methods). Deleting all *non-co-Ser1* codons (*no non-co-Ser1*), all *non-co-Ser2* codons (*no non-co-Ser2*), or both (*no non-co-Ser1, no non-co-Ser2*) together has minimal impact on bootstrap values in nucleotide likelihood analyses (on average −1% point; Table 1), suggesting that the phylogenetic signal from serine resides elsewhere. In contrast, deleting all *co-Ser1, co-Ser2*, or both together (*no co-Ser1, no co-Ser2, “no co-Ser1, no co-Ser2”*), leads to a major drop in bootstrap support (on average −34% points; Table 1), indicating that serine provides more phylogenetic signal at sites where *Ser1* and *Ser2* both appear.

Conversely, the retention of only serine at the *co-Ser* sites (*no non-Ser at co-Ser*, i.e., the deletion of all codons at *co-Ser* sites other than those encoding serine; Table S3) reduces bootstrap valeus markedly (≥15% points) for only one of the six nodes of interest (Xenocarida, −28% points), while the remain five nodes (Altocrustacea, Vericrustacea, Multicrustacea, Miracrustacea, and Edafopoda) are largely unaffected (on average −3.2% points, but Miracrustacea −10% points). This suggests that those five nodes, which are very sensitive to the elimination of codons for either *co-Ser1* or *co-Ser2* but not for other amino acids at the *co-Ser* sites, receive their strongest support directly from the distinction between *Ser1* and *Ser2* codons at *co-Ser* sites, i.e., the change between the *Ser* codon groups that is intermediate in rate between all of the other synonymous and nonsynonymous changes. On the other hand, the node Xenocarida (and to a lesser extent Miracrustacea), which is sensitive to the elimination of codons for either *co-Ser1* or *co-Ser2* and also for other amino acids at the *co-Ser* sites, is likely to receive strong support at those sites both from nonsynonymous substitutions not involving serine and from changes between the *Ser* codon groups. Interestingly, changes between *Ser* and non-serine codons at *co-Ser* sites appear to contribute little, determined by splitting *Ser* and *non-Ser* codons found at each *co-Ser* site into pairs of sites, one with *Ser1* and *Ser2* only and the other with *non-Ser* only, thereby ignoring informative change between *Ser* and *non-Ser* (*split co-Ser: non-Ser/Ser*). Regardless, all this demonstrates the value of distinguishing between *Ser1* and *Ser2* in phylogenetic analyses of this arthropod data set.

Apart from these six nodes of interest, three of the other 60 well supported nodes (Communostraca, Paleoptera, and Arachnida) have markedly reduced bootstrap values in the *no non-Ser at co-Ser* analysis (on average −29.5% points; Table S3), but as these nodes are not sensitive to the elimination of serine, this loss of support in itself is not informative about the usefulness of coding *Ser1* and *Ser2* separately.

To further demonstrate the utility of distinguishing them, *co-Ser1* and *co-Ser2* were one at a time recoded as a different amino acid – phenylalanine, tryptophan, or tyrosine – after first showing that deletion of any of these three proxy amino acids (*no Phe, no Trp, no Tyr)* was without major effect on resulting bootstrap values (on average 0% points; Table S3). With this recoding (*co-Ser1 to Phe, co-Ser1 to Trp, co-Ser1 to Tyr, co-Ser2 to Phe, co-ser2 to Trp, co-ser2 to Tyr*), amino acid bootstrap values increased on average 41% points (Table 1), oftentimes to levels comparable to that of nucleotides, indicating that the codon distinction and assignment of separate rate matrices to *Ser1* and *Ser2* was indeed beneficial, while the actual substitution rates were less critical. As a negative control, we showed that five other recodings not involving serine (*Asp to Glu, Gln to Asn, Ile to Ala, Phe to Tyr, Val to Ala*) had minimal impact on the selected six nodes (on average −4% points; Table S3).

### Degeneracy coding of nucleotide data matrices

So far in this report, our nonsynonymous nucleotide analyses have focused on *degen1* coding, and we have previously shown that *degen1* yields broadly consistent results with *noLRall1nt2* coding and a codon-model implementation ([5]; Figure 1, Table S1). We now introduce two new nonsynonymous codings, called *degen8* and *degenFS2* (see Materials and Methods and below). Each of the five approaches has advantages and disadvantages. *Degen1* operates on individual sequences and discards (through degeneration) selected nucleotides of individual sequences, but, as a means of dealing with the twofold degeneracy of leucine and arginine codons at the first codon position, it expands their coding capacity to leucine + phenylalanine (YTN) and arginine + *Ser2* (MGN), respectively. The novel *degen8* avoids this *degen1* coding problem by deleting a subset of leucine and arginine codons (TTR and AGR, respectively), rather than merging codons as for *degen1*. The results strongly resemble those of *degen1* in topology as well as support values. As a control of *degen1* and *degen8*, the novel *degenFS2* coding scheme avoids any coding artifacts through elimination of all phenylalanine and *Ser2* codons, yielding results similar to 20-amino-acid model analyses (Table S1). The *noLRall1nt2* coding method takes a more extreme approach than degeneracy coding in that entire characters are deleted. All third-codon-position characters are deleted, as well as all first-codon-position characters that encode one or more leucine or arginine residues, which also have the potential to undergo synonymous change. Finally, implementation of a codon model has the theoretical advantage that it does not delete (or degenerate) any characters, but it is computationally intensive and only indirectly and partially addresses compositional heterogeneity through the “downweighting” of rapidly evolving characters, namely, those that are enriched in synonymous change. The broad consistency of these five independent approaches (*degen1*, *degen8*, *degenFS2*, *noLRall1nt2* and codon model), all with slightly different assumptions, supports the correctness of their shared inferences.

If the codon groups *Ser1* and *Ser2* code for the same amino acid, why should they be distinguished in amino acid analyses?

**Figure 2:**
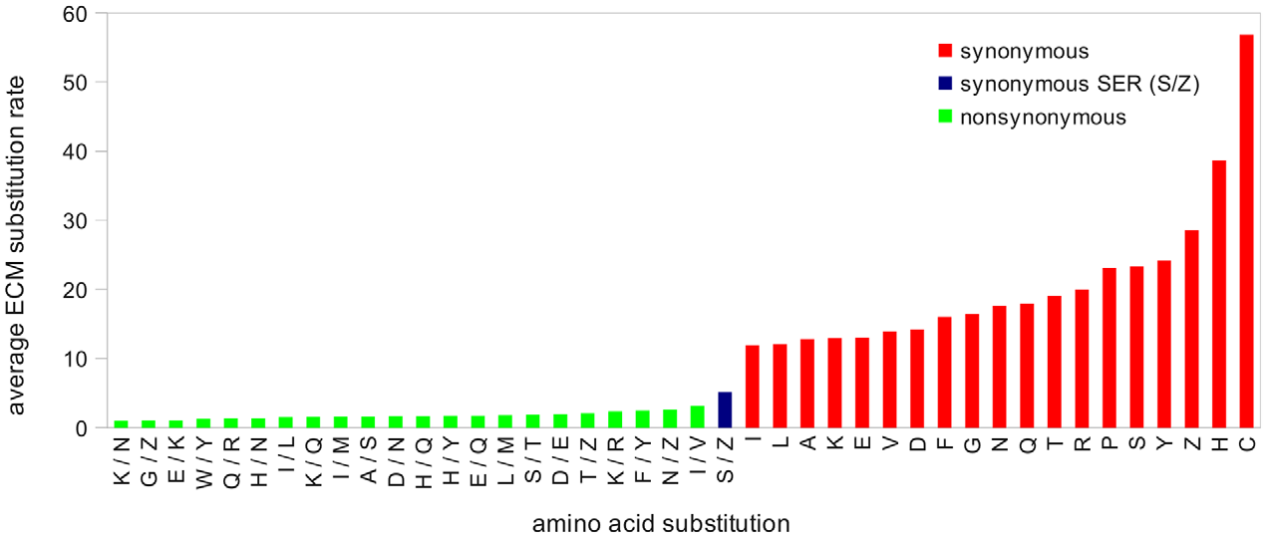
Plot of average ECM codon substitution rates for synonymous, intra-serine (S/Z), and the most frequent nonsynonymous substitutions. Individual codon rates are summarized through averaging for each respective amino acid (synonymous) or change between amino acids (synonymous SER (S/Z), nonsynonymous).

If one assumes that protein-coding sequence evolution occurs through individual nucleotide substitutions, the transformation between *Ser1* and *Ser2* codons must be a two-step process, involving a nonsynonymous intermediate codon. Only if two nucleotides change – at first and second positions of a single codon – in close succession or simultaneously might we expect the rate of such an apparent dinucleotide substitution to approach that of typical synonymous substitutions. Kosiol et al. [16] suggest that some such dinucleotide changes do occur essentially instantaneously on an evolutionary timescale, although the mechanism likely does involve a nonsynonymous change quickly followed by a compensatory change. Regardless, from the Empirical Codon Model ([16]) and the *21AA*-models introduced here (see Table S2), it is clear that the rate of *Ser1*-to-*Ser2* substitution is intermediate between typical synonymous and nonsynonymous changes. Plotting the average of the individual ECM rates, the average synonymous serine substitution rate (S/Z) lies between the highest nonsynonymous (*Ile/Val*) and the lowest synonymous (*Ile/Ile*) amino acid substitution rate (Figure 2). In terms of absolute values, the average rate of S/Z (5.1) is distinctly closer to the nonsynonymous *Ile/Val* (3.1) than to the synonymous *Ile/Ile* (11.9). The near-nonsynonymous rate for the effectively synonymous S/Z change and the unique mutational path between the underlying groups of serine codons argue for separate modeling of the two groups in amino acid analyses.

Likewise, the rates of change between *Ser1* and all other non-*Ser* amino acids are not the same as between *Ser2* and all other non-*Ser* amino acids (Table S2), which also argues that their separate coding is justified. *A fortiori*, it is also observed that, on average, rates of amino acid change from *Ser1* to other amino acids are faster than from *Ser2* to other amino acids when the former is based on a single nucleotide change but the latter on two nucleotide changes, and vice versa. For example, while the rate of *Ser1* (TCN) to *Ala* (GCN), *Phe* (TTY), and *Pro* (CCN) is about five times faster than for *Ser2* (AGY), the rate of *Ser2* to *Asn* (AAY) is about twice as fast as for *Ser1*. This clearly implies a constraint on the free interchange between *Ser1* and *Ser2*, and while not establishing an effect on phylogeny, argues that “synonymizing” *Ser1* and *Ser2* results in a loss of useful information. However, even separate modeling of S and Z at the amino acid level might be suboptimal, as the eight individual codon substitution rates that underly the average S/Z rate of 3.1 differ vastly in absolute values, ranging from 0.08 (AGT/TCC) to 12.17 (AGT/TCT; see Table S4). This is a 152-fold difference in rate for a single, synonymous substitution at the third codon position (TCC/TCT). Such great differences in rate can be accounted for in nucleotide and codon models, but are only averaged out in 20- and 21-amino-acid models. A separate observation arguing for the conservation of *Ser1* and *Ser2*, and thus for their separate modeling, is that there exist *non-co-Ser* sites (i.e, sites containing *Ser1* or *Ser2* but not both) for which the majority of taxa encode *Ser* rather than some other amino acid (Figure S3, Table S5), e.g., there are 76 *non-co-Ser* sites at which 50 or more taxa encode either *Ser1* or *Ser2*, which equates to 17% of all sites that contain *Ser* for that many taxa.

### Does separate coding of *Ser1* and *Ser2* give rise to non-phylogenetic signal in the analysis of this data set?

Substitutions that occur at different rates differ in their suitability for tracking phylogenetic splitting events of different ages. For example, relatively recent events are predominantly preserved in relatively fast evolving synonymous substitutions, where too few nonsynonymous substitutions might have accumulated. In this sense it is possible that intra-serine substitutions (S/Z), which are intermediate in average rate between standard synonymous and nonsynonymous substitutions (Figure 2), are particularly suitable to track the six relatively old and relatively short-branched nodes of interest. Conversely, the S/Z changes might have evolved too fast and become saturated, in which case they are no longer informative for these nodes, as may well be the case in reference [14], in which clade divergence times between dinoflagellates are vastly more ancient than those in the current report. However, lack of information *per se* will not cause incorrect phylogenetic estimates, and likelihood models can reduce the effect of by-chance similarities due to saturation through different site rates.

**Figure 3:**
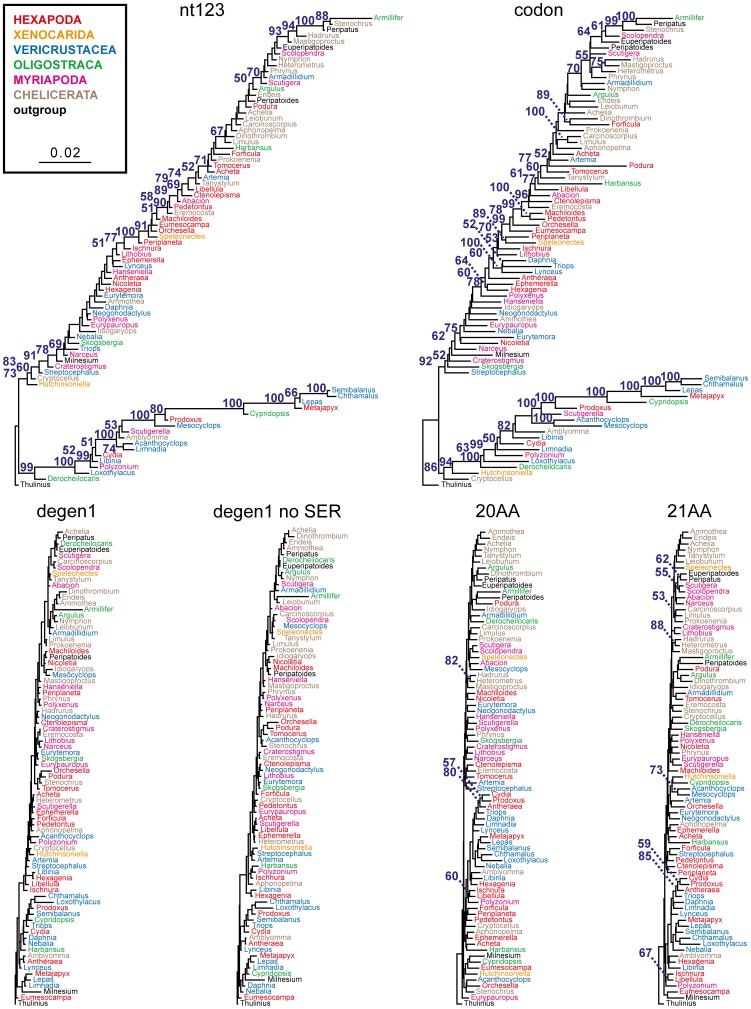
Compositional distance trees (Euclidean distances) for six data sets – nucleotide composition for *nt123* data set, degenerated nucleotide composition for *degen1* data sets with and without serine, codon composition for *codon* data set, and amino acid composition for *20AA* and *21AA* data sets. Bootstrap percentages >50% are displayed and indicate the strength of the compositional signal at particular nodes. The sum of all branch lengths reflects the total amount of compositional heterogeneity in the data set.

However, saturation at all levels (for both synonymous and nonsynonymous changes) is often linked to compositional heterogeneity, which is a far more serious concern for phylogenetic reconstructions than saturation, as all standard models assume compositional homogeneity. Also, *Ser1* and *Ser2* compositions can be differently biased across lineages, i.e., they are heterogeneous, and the magnitude of this heterogeneity might be sufficient to alter the phylogenetic inference from nucleotides, codons, and/or the 21 amino acids. A convenient and direct way to visualize such heterogeneity of any kind is by using composition, instead of primary sequence, as a character and to calculate a matrix of Euclidean distances for tree estimation. Figure 3 illustrates that the overall magnitude of heterogeneity (expressed as the sum of branch lengths) for total nucleotides (*nt123*) and codons (*codon*) is similar to each other but more than six times greater than for *degen1* (with and without *Ser*) and amino acids (*20AA* and *21AA*), which are themselves all quite similar to each other. In particular, the lack of significant differences between *degen1*-encoded data sets with and without serines, and the lack of strong differences between *20AA*- and *21AA*-amino-acid data sets argues directly against the introduction of significant compositional bias with separate coding of *Ser1/Ser2* and S/Z, respectively.

As a further way of illustrating the differences in strength of the non-phylogenetic compositional signal, the original sequence data sets have been bootstrapped and a bootstrap percentage calculated from their compositional distances. Both *nt123* and *codon* have numerous clades with bootstrap percentages above 50% and many of these clearly do not reflect phylogeny (see mixing of well established clades coded by color along with their associated bootstrap values in Figure 3). Conversely, *degen1* (with and without *Ser* nucleotides) have zero such supported groupings, strongly arguing that the contribution of compositional heterogeneity to the *degen1* phylogenetic inference must be relatively small. Interestingly, amino acids (both *20AA* and *21AA*) support a limited number of groups, but unlike *nt123* and *codon*, the groups recovered by amino acids are all likely to be valid phylogenetic groups. This difference in bootstrap values between *degen1* and amino acid compositions is likely to be due to the greater number of character states for amino acids, which results in more differentiated Euclidean distances. In this case (but not necessarily all cases) model violation by the amino acids (in the form of a significant contribution to the topology from composition) turns out to favor the correct phylogeny, which *degen1* supports based on primary sequence alone.

**Figure 4:**
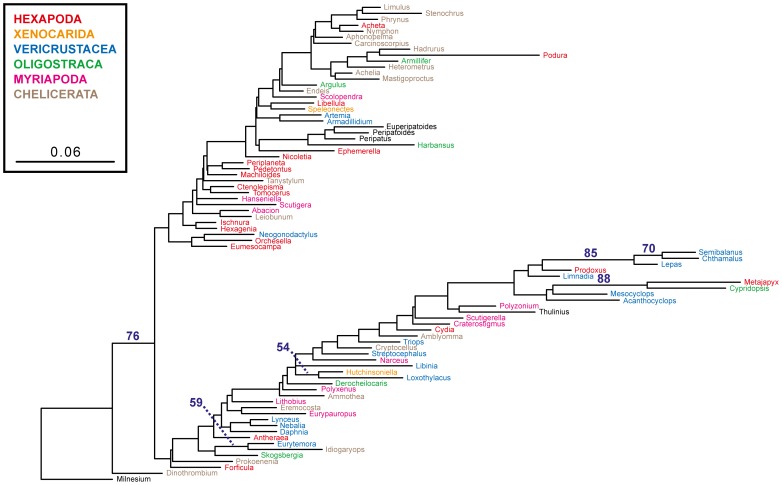
Compositional distance tree (Euclidean distances) based on the codon composition of a data set that is restricted to *co-Ser* codons. Bootstrap percentages >50% are displayed and indicate the strength of the compositional signal at particular nodes. The sum of all branch lengths reflects the total amount of compositional heterogeneity in the data set.

While Euclidean analysis of the total composition does not support the hypothesis of compositional heterogeneity as an obvious source of signal to explain the increased node support in the 21-amino-acid bootstrap analyses (Figure 1), one might still argue that, in focusing on *total* signal, the critical contribution from the *co-Ser* residues has been masked. To explore this possibility, Euclidean compositional distances using *only co-Ser* codons have been estimated (Figure 4). While compositional heterogeneity is apparent, the strength of the signal, as measured by the bootstrap, seems limited and does not group together those nodes of interest. Of the few nodes that are supported, some (e.g., the grouping of three species of Thecostraca) likely reflect common ancestry and are recovered in our phylogenetic analyses, while others (e.g., the grouping of a fourth species of Thecostraca with one from Ostracoda) neither reflect common ancestry nor are recovered in our phylogenetic analyses. The *co-Ser*-only degen1 and 21-amino-acid Euclidean-distance trees tell a similar story (Figures S4, S5). The proportions of *Ser1*:*Ser2* codons and *S*/*Z* residues have also been displayed for each of the 80 taxa (Figure S6). Again, there is substantial variation in codon usage but no striking correlation to taxonomic groups (N.B., the species in Table S6 are clustered by their higher-level classification to facilitate the visualization of relevant patterns). In summary, there is no evidence that the six nodes in question (see filled circles in Figure 1) are significantly influenced by compositional heterogeneity and/or a specific codon usage bias for *co-Ser*.

**Figure 5:**
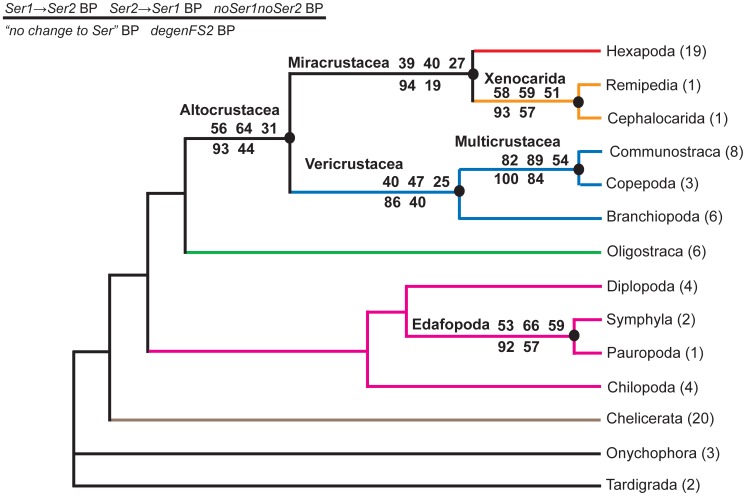
Summary of the six key nodes that are recovered in all maximum likelihood topologies from *degen1* analyses of five nucleotide data sets with and without modifications (including deletions) of serine codons, along with their bootstrap values. The complete topologies are condensed to illustrate that all six higher-level nodes under investigation are recovered by each of five data sets: 1. *Ser1→ Ser2* data set, in which *Ser1* codons (TCN) in the *degen1* data set are artificially changed to *Ser2* (AGY); 2. *Ser2→ Ser1* data set, in which all *Ser2* codons in the *degen1 data set* are artificially changed to *Ser1*; 3. *noSer1noSer2*, in which all *Ser1* and *Ser2* codons in the *degen1* data set are artificially changed to NNN; 4. *no change to Ser*, in which the *degen1* data set is analyzed as is; 5. *degenFS2*, in which all *Phe* (TTY) and *Ser2* (AGY) codons in the *degen1* data set are artificially changed to NNN.

The general argument might still be made that it would be questionable to rely so strongly for older divergences on faster evolving changes in *Ser1* and *Ser2*, whether at the level of amino acids or degenerated nucleotides. Using the *degen1* approach, we show that for the present data set even this concern is unwarranted (Figures 5, S7). In particular, whether TCN (*Ser1*) nucleotides are artificially converted to AGY (*Ser2*) or vice versa, thereby simulating the situation for amino acids, or whether all serines are removed (i.e., converted to NNN), the same six higher-level taxonomic groups are still recovered in their respective maximum likelihood topologies, albeit with greatly reduced bootstrap values. Despite a lack of strong statistical support values, the congruence in topology between those many different approaches demonstrates that the collective changes in the non-serine amino acids (and their encoded nucleotides) optimally support the same six nodes over all other alternatives. In other words, serine does not have an unusual signal – phylogenetic or otherwise – in this data set.

### A comment on the status of Chelicerata

The Chelicerata are traditionally a contentious node [18], [19] that, unlike most other deeper nodes, received little to moderate support in standard analyses using the current data set [5]. Similar to the six nodes of particular interest in this report, Chelicerata is more strongly supported with degeneracy coding (bootstrap, 74%; see Figure 1 in [5]) than with amino acids (bootstrap, 57%). However, unlike those six nodes the Chelicerata did not prove to be particularly sensitive to the analytical treatment of *Ser*. Instead, in that report we showed that, depending on which subsets of characters were selected, strong bootstrap support could be generated for conflicting placements of Pycnogonida, supporting either a monophyletic Chelicerata (up to 96% bootstrap) or a basal Pycnogonida within Arthropoda (up to 91% bootstrap, see Table 3 in [4]). Across the arthropod tree, conflicting signals were identified in only three other regions (1. position of Endeidae within Pycnogonida, 2. position of Diplopoda within Myriapoda, although never affecting the consistent recovery of Edafopoda, and 3. position of Polyzoniida within Diplopoda), but not in any of the six nodes considered in this report. The presence of a few conflicting nodes within an otherwise consistently strongly supported set of relationships is an interesting observation, but one that requires additional observations for a clearer understanding. As a result, we continue to think it unwise to speculate on the monophyly of Chelicerata based on analysis of this data set alone.

### Nomenclatural change

In a previous report [5], we proposed classificatory names for six higher-level taxonomic groupings within Pancrustacea, namely, Xenocarida, Miracrustacea, Altocrustacea, Vericrustacea, Multicrustacea, and Communostraca. However, we declined to name another novel but well supported (by nucleotides only) superclass grouping within Myriapoda, namely, Symphyla + Pauropoda, because of a long history of alternative proposals and a general sense from the literature that morphological evidence supported a closer placement of Pauropoda with Diplopoda ( = Dignatha) than with Symphyla (or Chilopoda) (reviewed in [20]). However, now that amino acids too provide strong support for Symphyla + Pauropoda (up to 89% bootstrap; Tables 1, S1), we hereby propose a name for this soil-dwelling clade, namely, “Edafopoda” (edafos = soil, podia = leg, foot; see also Nomenclatural Acts in the Materials and Methods section). We note that an earlier study of ribosomal genes also recovered Edafopoda [21].

### Conclusions

The different coding schemes, both nucleotide and amino acid, presented herein and previously [5], [6], enable valuable cross-checking of analytical assumptions with real-world data sets. The previously observed disparity [5] in levels of signal provided by nucleotides and amino acids is now resolved as due to the additional signal present as *Ser1* and *Ser2* in nucleotides that is missing in standard 20-amino-acid analyses, rather than a degeneracy coding artifact. The degeneracy coding schemes, in particular, are promising tools for the analysis of very large phylogenomic nucleotide data sets. Their main advantages over the use of codon and amino acid models are compatibility with different nucleotide models, analysis frameworks and software implementations, vastly lower computational requirements, and increased statistical power due to fewer parameters (see also [22], [23]). Complementarily, the novel 21-amino-acid models provide improved alternative methods for data analysis that may benefit from many more character states, albeit with fewer characters. However, the challenge of deep-level phylogeny, particularly when confounded by rapid radiations, is such that the concerted use of a variety of validated approaches should prove most useful.

Given the upswing in phylogenomics, the present analysis will not represent the final effort on inferring higher-level arthropod relationships, but we suggest that our conclusions ought to be taken seriously for the following reasons: Firstly, our taxon sampling, while modest, is extensive, including a number of difficult-to-obtain exemplars, e.g., Cephalocarida and Mystacocarida, not present in many previous studies. Secondly, we analyze a data set that is relatively large by current standards and analytically relatively tractable, since all 62 genes encode proteins made in the nucleus. Thirdly, we have previously shown that, for this data set, synonymous change is largely without phylogenetic content but with significant non-phylogenetic signal due to nucleotide heterogeneity [4], so we consider its removal to be both warranted and highly beneficial. Fourthly, results based on nucleotide (*degen1, degen8, noLRall1nt2*), codon, and amino acid (*21AA*) analyses are in large agreement. Indeed, even the 20-amino-acid ML topologies are in large agreement with the other analyses, albeit with reduced node support for the six higher-level nodes. Fifthly, we have demonstrated that heterogeneity at the levels of degenerated nucleotides does not contribute to the inferred topology based on sequence analysis, nor does the separate coding of *Ser1* and *Ser2* nucleotides introduce a non-phylogenetic signal. Lastly, our conclusions are based on a relatively stringent method for assessing node support, namely, the non-parametric bootstrap within a likelihood framework, which appears to provide a more fine-grained assessment of node support than posterior probabilities within a Bayesian framework [24], [25].

## Materials and Methods

### Taxon and gene sampling

The data set used is identical to one previously published [5]. Genus and species names and higher-level classification of all sampled taxa are shown in Figure S1. The alignment consists of 41,976 bp from 62 single-copy, nuclear protein-coding genes, which equates to 39,261 bp for analysis after exclusion of unalignable sites. See [5] for Genbank accession numbers and the aligned data set.

### Novel 20/21 amino acid models

To distinguish between *Ser1* (TCN) and *Ser2* (AGY), three 21-amino-acid models with corresponding amino acid rate matrices were implemented in GARLI V2.0 (http://code.google.com/p/garli/) [17].

1. *21AA-GTR*. GTR model rates are estimated from the actual data set. In this expanded 21-amino-acid model, amino acid *S* is restricted to the codon group *Ser1* (TCN), while the “novel amino acid” *Z* is encoded by codon group *Ser2* (AGY).
2. *20/21AA-ECM*. Kosiol et al. [16] published an Empirical Codon Model (ECM), which is based on nucleotide sequences in the multiple sequence alignment and phylogenetic tree database PANDIT [26], [27]. By summing up all individual ECM rates of codons that encode for the same amino acid (with *Z* for *Ser2* in the *21AA-ECM*), relative rates (Table S3) were estimated for the two novel amino acid models.
3. *21AA-JTT*. Rates for *Ser1* and *Ser2* were estimated by applying the proportions of *Ser1* and *Ser2* in the above 21AA-ECM rate matrix. Rates between the two serine codon groups and other amino acids were determined by splitting the original JTT rates for *Ser* proportionally to the 21AA-ECM rates for *Ser1* and *Ser2* relative to the total *Ser* rate. The rate between *Ser1* and *Ser2* was estimated by obtaining a rate between *Ser1* to *Ser2* relative to the average of all other rates in the 21AA-ECM matrix (see Table S3).

### Data set translation

Nucleotide data were translated internally by GARLI [17] (for ML analyses) and by the software MacClade [28] (for MP analyses).

### Data set encoding

1. **degen1.** In-frame codons of the same amino acid are fully degenerated with respect to synonymous change, e.g., CAT→CAY. Leu codons (TTR+CTN) are degenerated to Leu+Phe (YTN), and Arg codons (AGR+CGN) are degenerated to Arg+Ser2 (MGN) [5]. Phe and Ser2 are degenerated to TTY and AGY, respectively.
2. **degen8.** As for *degen1* except that a subset of Leu codons (TTR) and Arg codons (AGR) are completely degenerated (NNN). The remainder of the Leu and Arg codons in the data set are now coded as CTN and CGN, respectively.
3. **degenFS2.** As for *degen1* except that Phe codons (TTY) and *Ser2* codons (AGY) are completely degenerated (NNN).
4. **noLRall1nt2.** All third-codon-position characters are deleted, as are those first-codon-position characters that encode any and all Leu or Arg [6].

All scripts are available at http://www.phylotools.com. Complementary versions of degeneracy coding for other genetic codes, e.g., mitochondrial, are also available at this address.

### Data set manipulation

Data sets were selectively manipulated in four general ways and analyzed as *degen1*-encoded nucleotides and as amino acids.

1. The significance of serine codons groups (TCN and AGY) was tested by deleting them (changing to “NNN”). Data sets with Ser deletions come in three flavors: all Ser codons deleted (“*no Ser1, Ser2*”), all *Ser1* or *Ser2* codons deleted at those alignment positions at which they co-occur (“*no co-Ser1, no co-Ser2*”, “*no co-Ser1*”, “*no co-Ser2*”), and all *Ser1* or *Ser2* codons deleted at those alignment positions at which they do not co-occur (“*no non-co-Ser1, no non-co-Ser2*”, “*no non-co-Ser1*”, “*no non-co-Ser2*”).
2. The effect of not distinguishing between the two codon groups of *Ser*, as is standard in the case of 20-amino-acid analyses, was tested in nucleotide (*degen1*) analyses by substituting or over-writing one codon group with the other (“*Ser1 to Ser2*”, “*Ser2 to Ser1*”). Alternatively, an artificial distinction between the two codon groups in amino acid analyses was achieved by substituting a commingling codon group (*co-Ser1*, *co-Ser2*) with the codon of a different amino acid (“*co-Ser1 to Phe*”, “*co-Ser1 to Trp*”, “*co-Ser1 to Tyr*”, and “*co-Ser2 to Phe*”, “*co-Ser2 to Trp*”, “*co-Ser2 to Tyr*”).
3. To test the importance of these amino acid “proxies” (see category 2) on the nodes of interest, the codons of these amino acids were deleted (“*no Phe*”, “*no Trp*”, “*no Tyr*”).
4. To test the influence of codon substitutions on the nodes of interest in general, codons for amino acids of different frequencies and rates were substituted with others (“*Asp to Glu*”, “*Gln to Asn*”, “*Ile to Ala*”, “*Phe to Tyr*” and “*Val to Ala*”).
5. To test the relative importance of *Ser* and *non-Ser* at *co-Ser* sites in the alignment, non-Ser codons were removed at *co-Ser* sites (“*no non-Ser at co-Ser*”) for comparison with removal of *co-Ser* codons at *co-Ser* sites (“*no co-Ser1, no co-Ser2*”).
6. To test whether changes between *Ser* and *non-Ser* at *co-Ser* sites were informative, *co-Ser* sites in the alignment were each split into pairs of sites such that one member contained only *non-Ser* codons and the other, only *Ser* codons (“*split co-Ser: non-Ser/Ser*”).

These manipulations were carried out with perl scripts available at http://www.phylotools.com.

### Assessment of compositional heterogeneity

The compositional heterogeneity of the units (nucleotides, codons or amino acids) of a data set can be quantified through pairwise Euclidean distances of the unit compositions between sequences and visualized as an arbitrarily rooted distance tree. These Euclidean distances are the square-root of the sum of the squared differences between the proportions of the different units of any given sequence pair (see [29] for nucleotides) and, being based on composition alone, do not represent phylogenetic signal. The length of branches is correlated with the amount of compositional heterogeneity, and the longer a compositional distance tree is, the greater is the overall compositional heterogeneity of its underlying data set.

For the present data set, compositional distance matrices were calculated with a Perl script (available at http://www.phylotools.com) for nucleotides, codons, and 20 and 21 amino acids. Based on these matrices, distance trees were calculated in PAUP* [30] with a heuristic search under the minimum evolution criterion. To get a better assessment of distinct compositional similarities between individual taxa beyond subtending branch lengths, bootstrap values were estimated for the compositional distance trees. For bootstrapping with 300 pseudo-replicates, 300 randomly resampled data sets and their respective compositional distance matrices were generated with the Perl script. Bootstrap values are based on the majority rule consensus of the corresponding 300 distance trees.

### Data analysis

Computations took place on computing grid resources at the University of Kansas and through The Lattice Project [31] at the University of Maryland. All maximum-likelihood (ML) analyses were carried out with the software GARLI, versions 1.0 and 2.0 [17]. Amino acid (AA) analyses used the 20AA and 21AA versions of the models GTR, ECM [16] and JTT [12], each with a proportion of invariable sites (+I), and a gamma distribution for among site rate variation (+G). Equilibrium amino acid frequencies were those observed in the data set (+F). Amino-acid GTR rate matrices were estimated in 100 ML searches, with the matrix giving the highest likelihood fixed for corresponding bootstrap analyses. The nucleotide model applied to all data sets was the best-fitting standard GTR+I+G model. Bootstrap analyses consisted of 500 pseudoreplicates with three (for AA) or two heuristic searches beginning from random topologies. Results for *degen1*, *noLRall1nt2* and codon model analyses are only for comparison and are based on more than 1,000 single-search bootstrap pseudo-replicates (105 with the codon model due to computational limitations) [5]. Maximum parsimony (MP) analyses were carried out in PAUP* version 4.0b10 for PPC, using TBR and random step-wise sequence addition for 250 heuristic searches and 1,000 bootstrap pseudoreplicates with 5 searches each [30].

### Nomenclatural acts

The electronic edition of this article conforms to the requirements of the amended International Code of Zoological Nomenclature, and hence the new name contained herein is available under that Code from the electronic edition of this article. This published work and the nomenclatural acts it contains have been registered in ZooBank, the online registration system for the ICZN. The ZooBank LSIDs (Life Science Identifiers) can be resolved and the associated information viewed through any standard web browser by appending the LSID to the prefix “http://zoobank.org/”. The LSID for this publication is: urn:lsid:zoobank.org:pub:77C2B51C-F5DB-4FCA-A98E-9AAF7A70EA94. The electronic edition of this work was published in a journal with an ISSN, and has been archived and is available from the following digital repositories: PubMed Central, LOCKSS.

## Supporting Information

Figure S1 **Arthropod relationships and classification scheme based on** ***degen1*** **analysis of 75 ingroup plus five outgroup species** [**5**] **.**
(PDF)

Figure S2 **Strict consensus of four maximum parsimony trees for** ***21AA*** **data set plus bootstrap values (above branches) from** ***20AA*** **(left) and** ***21AA*** **(right) analyses.**
(PDF)

Figure S3 **Number of** ***Ser*** **-containing alignment sites in relation to the number of taxa that encode** ***Ser*** **at those sites.**
(PDF)

Figure S4 **Compositional distance tree (Euclidean distances) based on the nucleotide composition of a degen1-encoded data set that is restricted to** ***co-Ser*** **residues.** Bootstrap percentages >50% are displayed and indicate the strength of the compositional signal at particular nodes. The sum of all branch lengths reflects the total amount of compositional heterogeneity in the data set.
(PDF)

Figure S5 **Compositional distance tree (Euclidean distances) based on the amino acid composition of a 21-amino-acid data set that is restricted to** ***co-Ser*** **(** ***S/Z*** **) residues.** Bootstrap percentages >50% are displayed and indicate the strength of the compositional signal at particular nodes. The sum of all branch lengths reflects the total amount of compositional heterogeneity in the data set.
(PDF)

Figure S6 **Proportions of the six distinct** ***Ser*** **codons for each of the 80 taxa in this study.** Taxa are clustered by their higher-level classification to demonstrate that, in general, there is substantial variation in codon usage within higher-level groups, as well as across them.
(PDF)

Figure S7 **Maximum Likelihood tree based on a nucleotide model analysis (GTR+G+I) of a** ***degen1*** **-encoded data set that lacks all serine-coding nucleotides/codons.** The six nodes of particular interest (Xenocarida, Multicrustacea, Altocrustacea, Vericrustacea, Miracrustacea, Edafopoda) are recovered, indicating that the strong signal provided by serine codons is congruent with the signal provided by codons of all other amino acids combined.
(PDF)

Table S1 **Bootstrap percentages for 80-taxon likelihood analyses. Bootstrap percentages for the 68 taxonomic groups (out of 78 total) that receive at least 80% values for one of four core analyses (** ***degen1*** **,** ***noLRall1nt2*** **, codon,** ***20AA*** **; see** **Figure 1** **in** [**5**] **).**
(PDF)

Table S2 **Amino acid rate matrices for** ***20/21AA-JTT*** **and** ***20/21AA-ECM*** **as implemented in GARLI V2.0.**
(PDF)

Table S3 **Effect of data set manipulations on bootstrap percentages of 68 taxonomic groups. Changes in bootstrap proportions are relative to the standard** ***degen1*** **and** ***20AA-JTT*** **values (Table S1).**
(PDF)

Table S4 **Individual substitution rates of the ECM model categorized by their effect on amino acids: synonymous, synonymous SER (S/Z) and nonsynonymous.**
(PDF)

Table S5 **Number of** ***Ser*** **-containing alignment sites in relation to the number of taxa that encode** ***Ser*** **at those sites.**
(PDF)
