# Supplementary material for: Resolving Discrepancy between Nucleotides and Amino Acids in Deep-Level Arthropod Phylogenomics: Differentiating Serine Codons in 21-Amino-Acid Models
Source: PLoS One. 2012 Nov 20;7(11):e47450. doi: 10.1371/journal.pone.0047450 (PMC3502419; doi:10.1371/journal.pone.0047450)

Figure S3: Number of SER-containing alignment sites in relation to the number of taxa that encode SER at those sites. Both non-co-SER sites (encoding exclusively S or Z) and co-SER sites (encoding S and Z) have the number of taxa that encode S and/or Z, respectively, ranging from 2 to 80. The existence of non-co-SER sites with many taxa indicates that S and Z are not entirely freely interchangeable, i.e., that the different codon groups *Ser1* and *Ser2* are not truly synonymous. There is a total of 1,642 non-co- and 1,531 co-SER sites in the alignment, with about half of those non-co-SER sites (819) comprising only a single taxon that encodes S or Z (not shown in plot because of scaling).

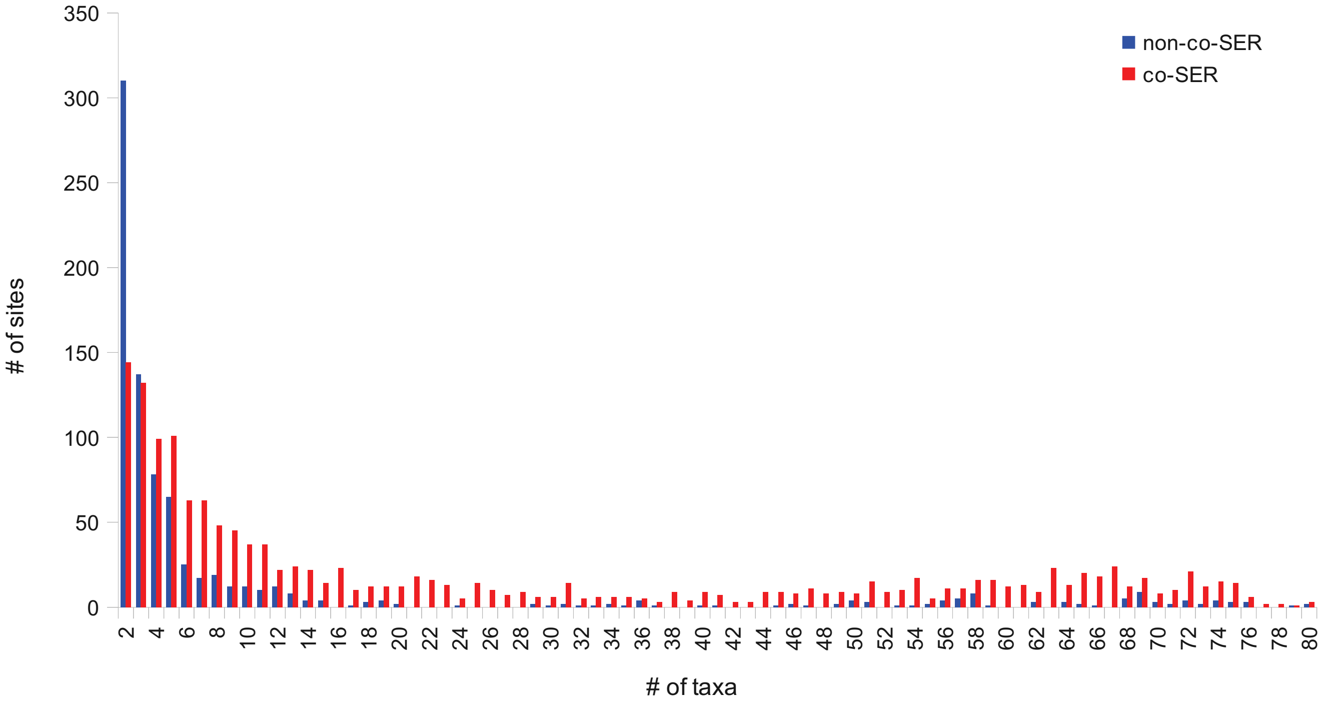

Supplement: Figure S3 — Number of Ser -containing alignment sites in relation to the number of taxa that encode Ser at those sites. (PDF) [file pone.0047450.s003.pdf]
