# Supplementary material for: Resolving Discrepancy between Nucleotides and Amino Acids in Deep-Level Arthropod Phylogenomics: Differentiating Serine Codons in 21-Amino-Acid Models
Source: PLoS One. 2012 Nov 20;7(11):e47450. doi: 10.1371/journal.pone.0047450 (PMC3502419; doi:10.1371/journal.pone.0047450)

Figure S4. Compositional distance tree (Euclidean distances) based on the nucleotide composition of a degen1-encoded data set that is restricted to *co-Ser* codons. Bootstrap percentages >50% are displayed and indicate the strength of the compositional signal at particular nodes. The sum of all branch lengths reflects the total amount of compositional heterogeneity in the data set.

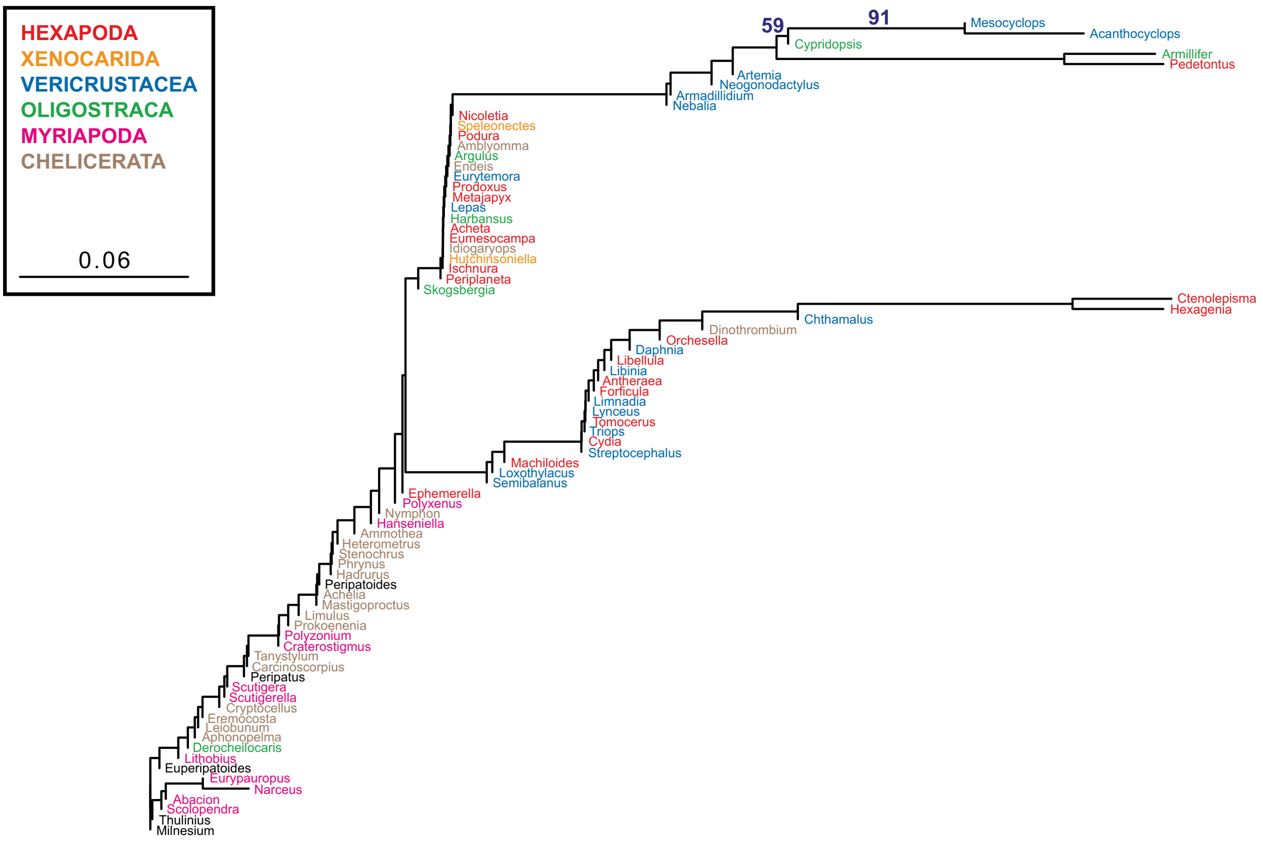

Supplement: Figure S4 — Compositional distance tree (Euclidean distances) based on the nucleotide composition of a degen1-encoded data set that is restricted to co-Ser residues. Bootstrap percentages >50% are displayed and indicate the strength of the compositional signal at particular nodes. The sum of all branch lengths reflects the total amount of compositional heterogeneity in the data set. (PDF) [file pone.0047450.s004.pdf]
