# Supplementary material for: Resolving Discrepancy between Nucleotides and Amino Acids in Deep-Level Arthropod Phylogenomics: Differentiating Serine Codons in 21-Amino-Acid Models
Source: PLoS One. 2012 Nov 20;7(11):e47450. doi: 10.1371/journal.pone.0047450 (PMC3502419; doi:10.1371/journal.pone.0047450)

Figure S5. Compositional distance tree (Euclidean distances) based on the amino acid composition of a 21-amino acid data set that is restricted to *co-SER* (S/Z) residues. Bootstrap percentages >50% are displayed and indicate the strength of the compositional signal at particular nodes. The sum of all branch lengths reflects the total amount of compositional heterogeneity in the data set.

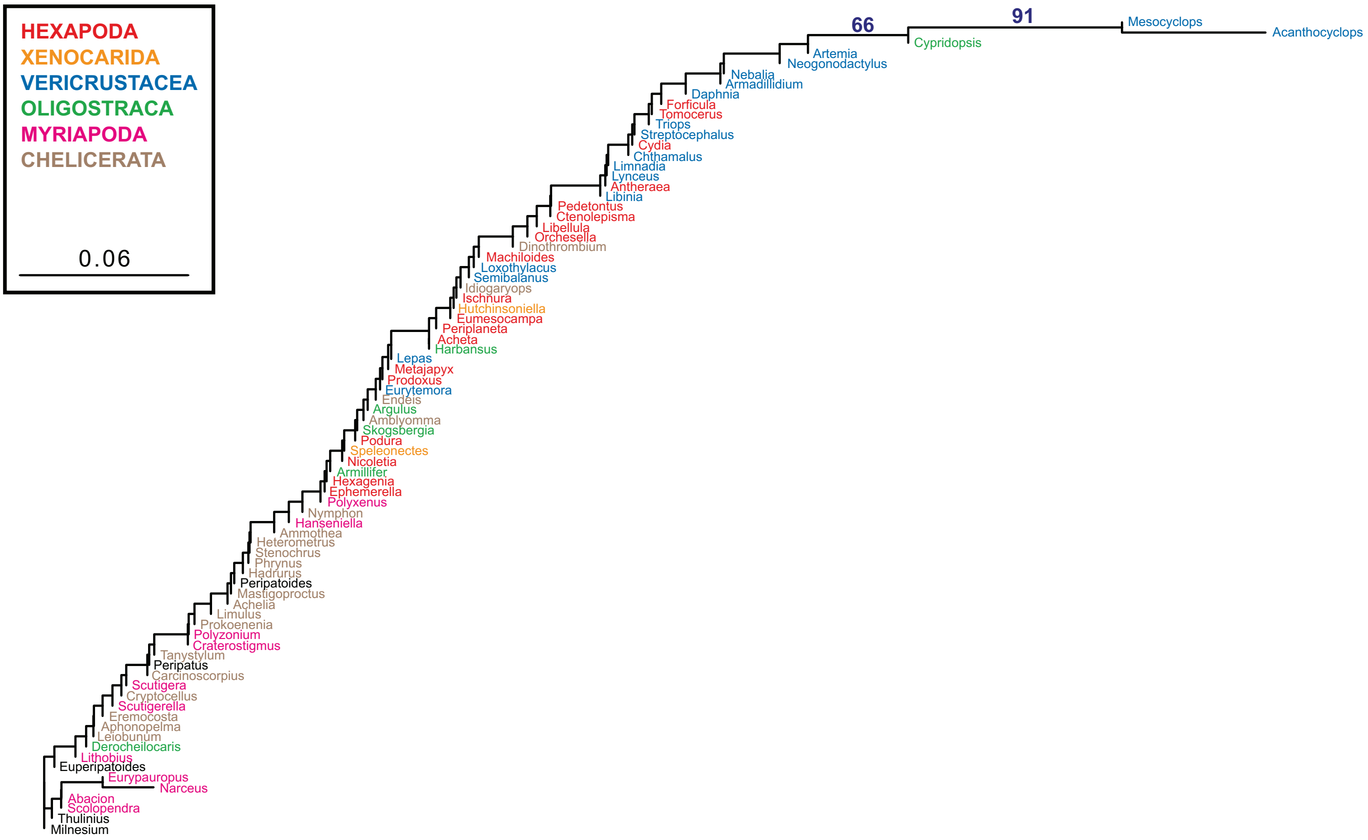

Supplement: Figure S5 — Compositional distance tree (Euclidean distances) based on the amino acid composition of a 21-amino-acid data set that is restricted to co-Ser ( S/Z ) residues. Bootstrap percentages >50% are displayed and indicate the strength of the compositional signal at particular nodes. The sum of all branch lengths reflects the total amount of compositional heterogeneity in the data set. (PDF) [file pone.0047450.s005.pdf]
