# Supplementary material for: Resolving Discrepancy between Nucleotides and Amino Acids in Deep-Level Arthropod Phylogenomics: Differentiating Serine Codons in 21-Amino-Acid Models
Source: PLoS One. 2012 Nov 20;7(11):e47450. doi: 10.1371/journal.pone.0047450 (PMC3502419; doi:10.1371/journal.pone.0047450)

Figure S6. Proportions of the six distinct *Ser* codons for each of the 80 taxa in this study. Taxa are clustered by their higher-level classification to demonstrate that, in general, there is substantial variation in codon usage within higher-levl groups, as well as across them.

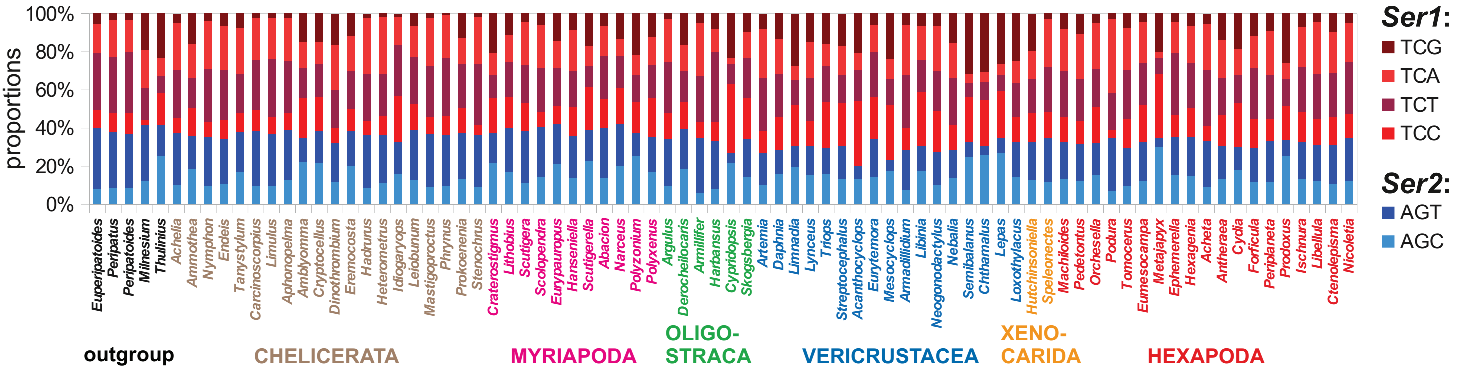

Supplement: Figure S6 — Proportions of the six distinct Ser codons for each of the 80 taxa in this study. Taxa are clustered by their higher-level classification to demonstrate that, in general, there is substantial variation in codon usage within higher-level groups, as well as across them. (PDF) [file pone.0047450.s006.pdf]
