# Supplementary material for: Resolving Discrepancy between Nucleotides and Amino Acids in Deep-Level Arthropod Phylogenomics: Differentiating Serine Codons in 21-Amino-Acid Models
Source: PLoS One. 2012 Nov 20;7(11):e47450. doi: 10.1371/journal.pone.0047450 (PMC3502419; doi:10.1371/journal.pone.0047450)

Figure S7. Maximum Likelihood tree based on a nucleotide model analysis (GTR+G+I) of a Degen1-encoded data set that lacks all serine-coding nucleotides / codons (*no-Ser1Ser2*). The six nodes of particular interest (Xenocarida, Multicrustacea, Vericrustacea, Altocrustacea, Miracrustacea, Edafopoda) are recovered, indicating that the strong signal provided by serine codons is congruent to the signal provided by codons of all other amino acids.

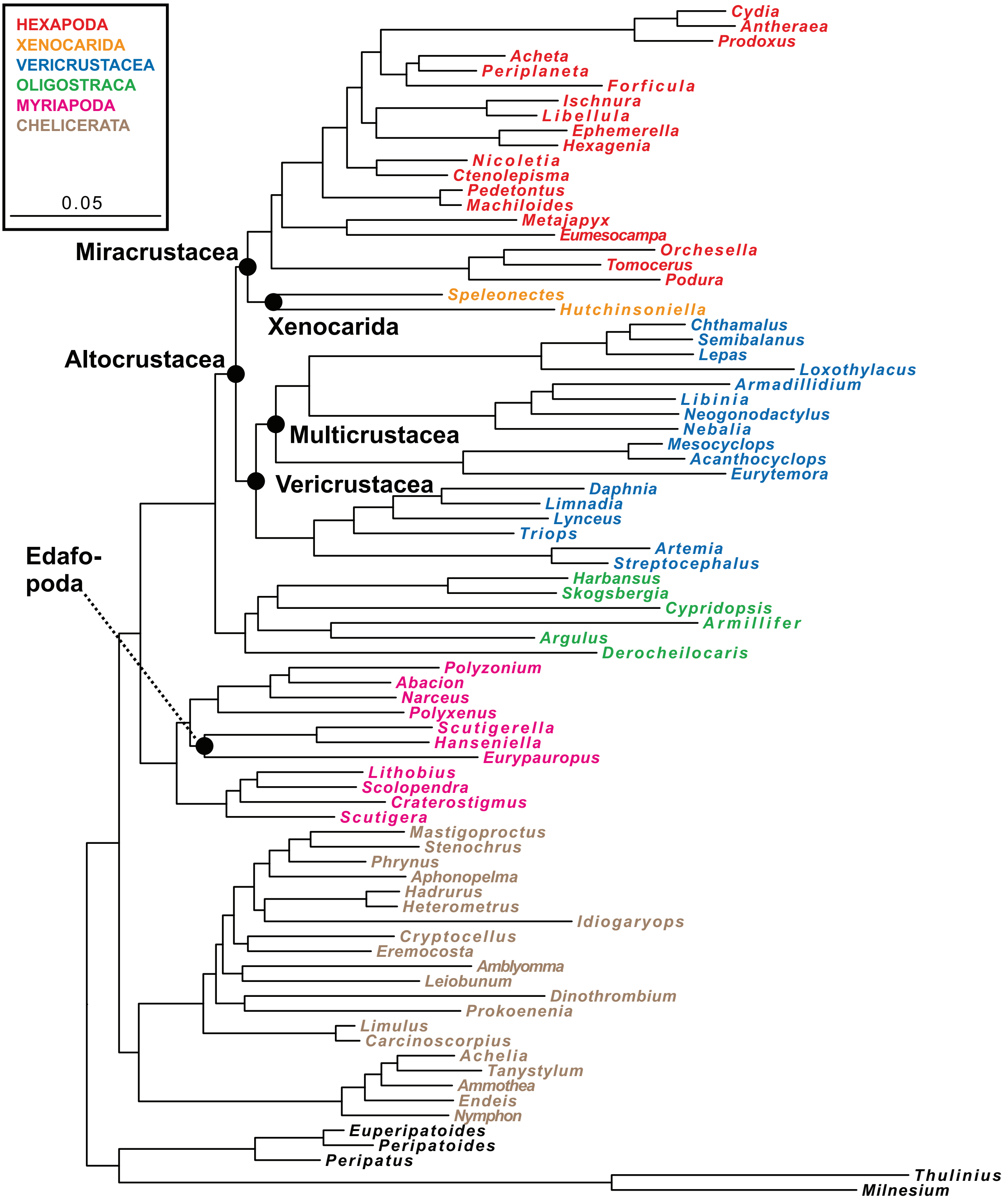

Supplement: Figure S7 — Maximum Likelihood tree based on a nucleotide model analysis (GTR+G+I) of a degen1 -encoded data set that lacks all serine-coding nucleotides/codons. The six nodes of particular interest (Xenocarida, Multicrustacea, Altocrustacea, Vericrustacea, Miracrustacea, Edafopoda) are recovered, indicating that the strong signal provided by serine codons is congruent with the signal provided by codons of all other amino acids combined. (PDF) [file pone.0047450.s007.pdf]
