# Supplementary material for: Resolving Discrepancy between Nucleotides and Amino Acids in Deep-Level Arthropod Phylogenomics: Differentiating Serine Codons in 21-Amino-Acid Models
Source: PLoS One. 2012 Nov 20;7(11):e47450. doi: 10.1371/journal.pone.0047450 (PMC3502419; doi:10.1371/journal.pone.0047450)
Supplement: Table S1 — Bootstrap percentages for 80-taxon likelihood analyses. Bootstrap percentages for the 68 taxonomic groups (out of 78 total) that receive at least 80% values for one of four core analyses ( degen1 , noLRall1nt2 , codon, 20AA ; see Figure 1 in [5] ). (PDF) [file pone.0047450.s008.pdf]

Table S1. Bootstrap percentages for 80-taxon likelihood analyses.

node index #	taxonomic group	standard [* = Regier *et al.* 2010]					*GTR*			*ECM*			*JTT*		
		degen1*	degenFS2	degen8	noLRall1 +nt2*	codon*	20AA	21AA	Δ (21AA - 20AA)	20AA	21AA	Δ (21AA - 20AA)	20AA	21AA	Δ (21AA - 20AA)
32	Edafopoda (=Symphyla + Pauropoda)	92	57	88	92	97	59	80	**21**	70	87	**17**	64	89	**25**
40	Altocrustacea	93	25	90	89	81	16	58	**41**	6	52	**46**	20	66	**46**
41	Vericrustacea	86	25	84	88	80	13	40	**27**	15	54	**39**	24	64	**40**
47	Multicrustacea	100	84	100	100	98	37	74	**37**	43	86	**43**	52	90	**38**
57	Miracrustacea	94	25	92	98	79	10	45	**35**	8	42	**34**	16	66	**50**
58	Xenocarida	93	57	90	100	89	39	70	**31**	27	64	**36**	55	87	**32**
1	Onychophora	100	100	100	100	100	100	100	0	100	100	0	100	100	0
2	Peripatopsidae	100	100	100	100	100	100	100	0	100	100	0	100	100	0
3	Tardigrada	100	100	100	100	100	100	100	0	100	100	0	100	100	0
4	Arthropoda	100	100	100	100	100	100	100	0	100	100	0	100	100	0
5	Pycnogonida	100	100	100	100	100	100	100	0	100	100	0	100	100	0
6	Ammotheidae + Endeididae	93	93	95	76	100	88	90	1	95	97	2	93	96	4
8	Ammotheidae	99	98	97	99	100	88	94	7	90	95	5	89	96	7
9	Tanystylum + Achelia	98	92	99	99	97	99	98	0	96	97	2	97	98	1
12	Euchelicerata	100	100	100	100	100	100	100	0	100	100	0	100	100	0
13	Xiphosura	100	100	100	100	100	100	100	0	100	100	0	100	100	0
14	Arachnida	68	76	57	80	39	17	22	4	24	15	-9	17	13	-4
15	Pulmonata	65	63	89	75	94	75	80	5	83	88	5	71	77	6
16	Scorpiones	100	100	100	100	100	100	100	0	100	100	0	100	100	0
17	Tetrapulmonata	99	97	99	97	97	97	99	2	98	100	2	98	99	1
18	Pedipalpi	100	100	100	85	100	94	95	1	97	95	-2	98	96	-2
19	Uropygi	100	100	100	98	100	100	100	0	99	100	1	99	100	1
20	Mandibulata	99	98	99	99	99	97	98	1	99	99	0	99	99	0
21	Myriapoda	100	100	100	100	100	100	100	0	100	100	0	100	100	0
22	Chilopoda	100	100	100	100	100	100	100	0	100	100	0	100	100	0
23	Pleurostigmophora	93	97	78	59	77	91	88	-3	89	84	-5	92	82	-10
24	Scolopendromorpha + Lithobiomorpha	99	99	95	99	100	99	99	0	99	99	0	97	99	2
27	Diplopoda	99	99	100	95	97	98	96	-2	98	97	0	96	98	1
28	Chilognatha	100	100	100	100	100	100	100	0	100	100	0	100	100	0
32	Edafopoda (=Symphyla + Pauropoda)	92	57	88	92	97	59	80	**21**	70	87	**17**	64	89	**25**
33	Symphyla	100	100	100	100	100	100	100	0	100	100	0	100	100	0
34	Pancrustacea	100	100	100	99	100	100	100	0	100	100	0	100	100	0
35	Oligostraca	100	99	99	95	92	99	100	0	93	99	5	98	100	2
37	Myodocopa	100	100	100	100	100	100	100	0	100	100	0	100	100	0
39	Ichthyostraca	100	100	100	100	100	100	100	0	100	100	0	100	100	0
40	Altocrustacea	93	25	90	89	81	16	58	**41**	6	52	**46**	20	66	**46**
41	Vericrustacea	86	25	84	88	80	13	40	**27**	15	54	**39**	24	64	**40**
42	Branchiopoda	100	100	100	100	100	100	100	0	100	100	0	100	100	0
43	Anostraca	100	100	100	100	100	100	100	0	100	100	0	100	100	0
44	Phyllopoda	100	100	100	100	100	100	100	0	100	100	0	100	100	0
45	Diplostraca	100	100	100	100	100	100	100	0	99	99	0	100	100	0
46	Cladocera + Spinicaudata	100	100	100	100	100	100	100	0	100	100	0	100	100	0
47	Multicrustacea	100	84	100	100	98	37	74	**37**	43	86	**43**	52	90	**38**
48	Copepoda	100	100	100	100	100	100	100	0	100	100	0	100	100	0
49	Cyclopoida	100	100	100	100	100	100	100	0	100	100	0	100	100	0
50	Communostraca	84	99	78	57	86	100	99	-1	97	93	-4	99	95	-3
51	Malacostraca	100	100	100	100	100	100	100	0	100	100	0	100	100	0
52	Eumalacostraca	100	100	100	100	100	100	100	0	100	100	0	100	100	0
53	Eucarida + Peracarida	87	84	68	51	58	90	92	2	81	84	3	87	89	2
54	Thecostraca	100	100	100	100	100	100	100	0	100	100	0	100	100	0
55	Thoracica	100	100	100	100	100	100	100	0	100	100	0	100	100	0
56	Sessilia	97	83	94	99	97	94	98	3	96	98	1	97	97	0
57	Miracrustacea	94	25	92	98	79	10	45	**35**	8	42	**34**	16	66	**50**
58	Xenocarida	93	57	90	100	89	39	70	**31**	27	64	**36**	55	87	**32**
59	Hexapoda	100	100	99	99	97	100	100	0	99	100	1	100	100	0
60	Entognatha	86	51	80	89	89	88	86	-2	96	97	0	85	91	6
61	Diplura	100	100	100	100	100	100	100	0	100	100	0	100	100	0
62	Collembola	100	100	100	100	100	100	100	0	100	100	0	100	100	0
63	Entomobryomorpha	98	87	99	95	79	96	98	2	98	98	0	97	96	-1
64	Insecta	100	100	100	100	100	100	100	0	100	100	0	100	100	0
65	Archaeognatha	100	100	100	100	100	100	100	0	100	100	0	100	100	0
66	Dicondylia	100	100	100	99	100	100	100	0	100	100	0	100	100	0
67	Zygentoma	100	100	100	100	100	100	100	0	100	100	0	100	100	0
68	Pterygota	99	93	99	99	92	100	100	0	100	99	-1	100	99	0
69	Paleoptera	69	84	76	59	76	91	89	-1	93	95	2	88	90	2
70	Ephemeroptera	100	100	100	100	100	100	100	0	100	100	0	100	100	0
71	Odonata	100	100	100	100	100	100	100	0	100	100	0	100	100	0
72	Neoptera	97	98	97	96	81	93	94	1	96	97	1	96	98	2
73	Polyneoptera	99	100	100	94	97	100	99	-1	100	97	-3	100	99	-1
74	Blattodea + Orthoptera	94	93	95	99	89	93	97	3	95	99	4	95	98	3
75	Lepidoptera	100	100	100	100	100	100	100	0	100	100	0	100	100	0
76	Ditrysia	100	100	100	100	100	100	100	0	100	100	0	100	100	0
