# Supplementary material for: Resolving Discrepancy between Nucleotides and Amino Acids in Deep-Level Arthropod Phylogenomics: Differentiating Serine Codons in 21-Amino-Acid Models
Source: PLoS One. 2012 Nov 20;7(11):e47450. doi: 10.1371/journal.pone.0047450 (PMC3502419; doi:10.1371/journal.pone.0047450)
Supplement: Table S2 — Amino acid rate matrices for 20/21AA-JTT and 20/21AA-ECM as implemented in GARLI V2.0. (PDF) [file pone.0047450.s009.pdf]

Table S2. Amino acid rate matrices for 20/21AA-JTT and 20/21AA-ECM as implemented in GARLI V2.0

21AA-ECM rate matrix (in the 20AA matrix, *S* rates are the sum of 21AA-ECM *S* and *Z* rates)

	A	C	D	E	F	G	H	I	K	L	M	N	P
**A**		5.144764	2.195588	4.229953	1.203338	10.943117	1.431657	2.066347	2.551994	5.777557	1.975596	1.408829	8.236222
**C**			0.171732	0.060174	1.418070	1.331899	1.118779	0.823508	0.150118	2.851527	0.622704	0.686527	0.473549
**D**				7.918377	0.119763	3.011624	1.870186	0.100626	1.272808	0.382254	0.070444	6.797966	2.466156
**E**					0.136811	2.187637	1.283762	0.444563	4.290266	0.999391	0.296459	1.691423	2.506945
**F**						0.520807	1.402246	3.381605	0.210995	11.691042	1.479972	0.407368	0.725283
**G**							1.196159	0.320999	1.710345	1.192392	0.494005	4.119096	2.723789
**H**								0.362077	1.938735	2.340967	0.486994	5.544322	2.452656
**I**									1.126065	27.775065	4.814082	0.731755	1.070870
**K**										1.643642	0.772948	4.052440	2.400410
**L**											11.007710	0.929799	4.139546
**M**												0.428094	0.325551
**N**													1.588971
**P**													
**Q**													
**R**													
**S**													
**T**													
**V**													
**W**													
**Y**													
**Z**													

									RESCALE FACTORS	
	Q	R	S	T	V	W	Y	Z	S'	Z
**A**	3.789726	6.454848	26.021204	12.810670	14.884027	0.557519	1.096362	4.939981	0.840446	0.159554
**C**	0.358913	2.308456	5.127768	2.602089	4.376476	0.474671	1.574639	3.810018	0.573718	0.426282
**D**	1.887363	1.302153	3.291162	2.177254	0.421621	0.094633	0.433956	3.721020	0.469349	0.530651
**E**	6.998262	5.080131	3.039828	3.371424	1.553576	0.147600	0.480090	2.092705	0.592267	0.407733
**F**	0.259926	0.712359	2.079796	1.004076	2.955317	1.970208	9.785921	0.392400	0.841275	0.158725
**G**	1.587673	5.688727	6.142395	2.094325	1.413316	0.599971	0.392917	8.467722	0.420421	0.579579
**H**	6.834790	10.538877	2.732229	2.153889	0.928450	0.564346	6.888784	2.581263	0.514206	0.485794
**I**	0.589348	1.823729	0.693063	5.974187	37.318690	0.385202	1.207469	0.938984	0.424659	0.575341
**K**	6.247636	28.489097	3.040150	5.005667	1.352499	0.156231	0.723809	2.945964	0.507867	0.492133
**L**	3.985715	6.692981	4.582890	4.988749	17.725189	1.549814	2.797498	0.679785	0.870829	0.129171
**M**	1.278593	1.694322	0.908872	3.084096	2.749346	0.321642	0.594722	0.610956	0.598010	0.401990
**N**	3.329175	3.906960	4.722081	6.317646	0.559798	0.128767	1.325032	10.329447	0.313728	0.686272
**P**	3.162432	6.287485	13.191683	5.919012	2.849121	0.380493	0.748452	2.506169	0.840350	0.159650
**Q**		16.439866	4.651786	4.519492	1.319533	0.302653	0.989983	2.912063	0.615002	0.384998
**R**			5.622100	8.803644	2.922289	2.120534	2.276895	9.583104	0.369748	0.630252
**S**				30.098825	2.234643	0.756949	2.013691	40.754475		
**T**					12.929044	0.496863	1.475472	16.815460	0.641571	0.358429
**V**						0.466495	1.577329	0.895539	0.713902	0.286098
**W**							2.562047	0.306184	0.711998	0.288002
**Y**								0.853656	0.702284	0.297716
**Z**										

**AVERAGE** (excl. Z/S):	3.641275
**Absolute Z/S:**	40.754475
**Z/S relative to average:**	**11.192365**

The above relative rates are the sum of all individual ECM rates (Kosiol *et al*. 2007) of codons that encode for the same amino acid (with *S* (*Ser1*) and *Z* (*Ser2)* for the *21AA-ECM*). Rescale factors and averages see below (*21AA-JTT*).

20AA-JTT relative rate matrix as implemented in GARLI V2.0

	A	C	D	E	F	G	H	I	K	L	M	N	P
A		0.056	0.081	0.105	0.015	0.179	0.027	0.036	0.035	0.030	0.054	0.054	0.194
C			0.010	0.005	0.078	0.059	0.069	0.017	0.007	0.023	0.031	0.034	0.014
D				0.767	0.004	0.130	0.112	0.011	0.026	0.007	0.015	0.528	0.015
E					0.005	0.119	0.026	0.012	0.181	0.009	0.018	0.058	0.018
F						0.005	0.040	0.089	0.004	0.248	0.043	0.010	0.017
G							0.023	0.006	0.027	0.006	0.014	0.081	0.024
H								0.016	0.045	0.056	0.033	0.391	0.115
I									0.021	0.229	0.479	0.047	0.010
K										0.014	0.065	0.263	0.021
L											0.388	0.012	0.102
M												0.030	0.016
N													0.015
P													
Q													
R													
S													
T													
V													
W													
Y													
Z													

	Q	R	S	T	V	W	Y
A	0.057	0.058	0.378	0.475	0.298	0.009	0.011
C	0.009	0.113	0.223	0.042	0.062	0.115	0.209
D	0.049	0.016	0.059	0.038	0.031	0.004	0.046
E	0.323	0.029	0.030	0.032	0.045	0.010	0.007
F	0.004	0.005	0.092	0.012	0.062	0.053	0.536
G	0.026	0.137	0.201	0.033	0.047	0.055	0.008
H	0.597	0.328	0.073	0.046	0.011	0.008	0.573
I	0.009	0.022	0.040	0.245	0.961	0.009	0.032
K	0.292	0.646	0.047	0.103	0.014	0.010	0.008
L	0.072	0.038	0.059	0.025	0.180	0.052	0.024
M	0.043	0.044	0.029	0.226	0.323	0.024	0.018
N	0.086	0.045	0.503	0.232	0.016	0.008	0.070
P	0.164	0.074	0.285	0.118	0.023	0.006	0.010
Q		0.310	0.053	0.051	0.020	0.018	0.024
R			0.101	0.064	0.017	0.126	0.020
S				0.477	0.038	0.035	0.063
T					0.112	0.012	0.021
V						0.025	0.016
W							0.071
Y							
Z							

21AA-JTT matrix based on the above 20AA-JTT matrix, modified by ECM derived rescale factors with relative S rates multiplied by S' factors, and relative Z rates being relative S rates multiplied by Z factors; S/Z rate is relative to the average rate of all other amino acids

	A	C	D	E	F	G	H	I	K	L	M	N	P
**A**		0.056	0.081	0.105	0.015	0.179	0.027	0.036	0.035	0.030	0.054	0.054	0.194
**C**			0.010	0.005	0.078	0.059	0.069	0.017	0.007	0.023	0.031	0.034	0.014
**D**				0.767	0.004	0.130	0.112	0.011	0.026	0.007	0.015	0.528	0.015
**E**					0.005	0.119	0.026	0.012	0.181	0.009	0.018	0.058	0.018
**F**						0.005	0.040	0.089	0.004	0.248	0.043	0.010	0.017
**G**							0.023	0.006	0.027	0.006	0.014	0.081	0.024
**H**								0.016	0.045	0.056	0.033	0.391	0.115
**I**									0.021	0.229	0.479	0.047	0.010
**K**										0.014	0.065	0.263	0.021
**L**											0.388	0.012	0.102
**M**												0.030	0.016
**N**													0.015
**P**													
**Q**													
**R**													
**S**													
**T**													
**V**													
**W**													
**Y**													
**Z**													

	Q	R	S	T	V	W	Y	Z
**A**	0.057	0.058	0.318	0.475	0.298	0.009	0.011	0.060
**C**	0.009	0.113	0.128	0.042	0.062	0.115	0.209	0.095
**D**	0.049	0.016	0.028	0.038	0.031	0.004	0.046	0.031
**E**	0.323	0.029	0.018	0.032	0.045	0.010	0.007	0.012
**F**	0.004	0.005	0.077	0.012	0.062	0.053	0.536	0.015
**G**	0.026	0.137	0.085	0.033	0.047	0.055	0.008	0.116
**H**	0.597	0.328	0.038	0.046	0.011	0.008	0.573	0.035
**I**	0.009	0.022	0.017	0.245	0.961	0.009	0.032	0.023
**K**	0.292	0.646	0.024	0.103	0.014	0.010	0.008	0.023
**L**	0.072	0.038	0.051	0.025	0.180	0.052	0.024	0.008
**M**	0.043	0.044	0.017	0.226	0.323	0.024	0.018	0.012
**N**	0.086	0.045	0.158	0.232	0.016	0.008	0.070	0.345
**P**	0.164	0.074	0.239	0.118	0.023	0.006	0.010	0.046
**Q**		0.310	0.033	0.051	0.020	0.018	0.024	0.020
**R**			0.037	0.064	0.017	0.126	0.020	0.064
**S**				0.477	0.038	0.035	0.063	**1.022**
**T**					0.112	0.012	0.021	0.171
**V**						0.025	0.016	0.011
**W**							0.071	0.010
**Y**								0.019
**Z**								

**AVERAGE** (excl. Z/S):	0.091
**Z/S** (= average * relative ECM Z/S):	**1.022**

Rates for *S* and *Z* were estimated by applying the proportions of *S* and *Z* in the above *21AA-ECM* rate matrix. Rates between the two serine codon groups and other amino acids were determined by splitting the original JTT rates for serine proportionally to the *21AA-ECM* rates for *S* and *Z* relative to the total serine rate ("Rescale factors"). For example, in the case of alanine (A), the *21AA-ECM* rate for *S* to A is 26.02 and for *Z* to A 4.94. Hence, the proportion of *S* and *Z* for a total serine rate is 0.84 and 0.16, respectively. In the *20AA-JTT* model implemented in GARLI, the serine to alanine rate is 0.38, hence, multiplying this rate with the *S* and *Z* proportions results in rates of 0.32 and 0.06 for *21AA-JTT S* and *Z*, respectively. This approach can't be used to determine the rate between *S* and *Z*, as it would be a rate relative to itself (the synonymous substitution rate of serine). Hence, this factor was estimated by obtaining a rate between *S* to *Z* relative to the average of all other rates in the *21AA-ECM* matrix. Multiplying the average rate of the *20AA-JTT* matrix with this factor provides the *S* to *Z* rate in the *21AA-JTT* matrix. In the *21AA-ECM* matrix, the absolute *S* to *Z* rate is 40.75 and the average of all other rates 3.64, resulting in a *S* to *Z* rate of 11.19 relative to the average. The average rate in the *20AA-JTT* model implemented in GARLI is 0.09, which results in an absolute rate of 1.02 for the *21AA-JTT S* to *Z* rate when applying the relative factor of 11.19.
