# Supplementary material for: Resolving Discrepancy between Nucleotides and Amino Acids in Deep-Level Arthropod Phylogenomics: Differentiating Serine Codons in 21-Amino-Acid Models
Source: PLoS One. 2012 Nov 20;7(11):e47450. doi: 10.1371/journal.pone.0047450 (PMC3502419; doi:10.1371/journal.pone.0047450)
Supplement: Table S3 — Effect of data set manipulations on bootstrap percentages of 68 taxonomic groups. Changes in bootstrap proportions are relative to the standard degen1 and 20AA-JTT values (Table S1). (PDF) [file pone.0047450.s010.pdf]

Table S3. Effect of data set manipulations on bootstrap percentages of all taxonomic groups.
Changes in bootstrap proportions are relative to the standard degen1 and 20AA-JTT values (Supplementary Table 1).

node index #	taxonomic group	*no Ser1, no Ser2*				*no co-Ser1, no co-Ser2*				*no co-Ser1*				*no co-Ser2*			
		degen 1	Δ (no Ser1, no Ser2 - standard)	20AA	Δ (no Ser1, no Ser2 - standard)	degen 1	Δ (no co-Ser1, no co-Ser2 - standard)	20AA	Δ (no co-Ser1, no co-Ser2 - standard)	degen 1	Δ (no co-Ser1 - standard)	20AA	Δ (no co-Ser1 - standard)	degen 1	Δ (no co-Ser2 - standard)	20AA	Δ (no co-Ser2 - standard)
32	Edafopoda (=Symphyla + Pauropoda)	59	**-33**	66	2	52	**-40**	73	9	60	**-32**	68	4	59	**-33**	71	7
40	Altocrustacea	32	**-61**	7	-13	51	**-42**	8	-12	55	**-38**	8	-12	52	**-41**	13	-7
41	Vericrustacea	26	**-60**	13	-11	47	**-39**	11	-13	41	**-45**	8	**-16**	53	**-33**	16	-7
47	Multicrustacea	53	**-47**	29	**-23**	75	**-25**	34	**-18**	75	**-25**	40	-12	92	-8	47	-5
57	Miracrustacea	27	**-67**	13	-3	38	**-56**	20	4	42	**-52**	15	-1	33	**-61**	4	-12
58	Xenocarida	52	**-41**	44	-11	68	**-25**	60	5	69	**-24**	73	**18**	61	**-32**	55	0
1	Onychophora	100	0	100	0	100	0	100	0	100	0	100	0	100	0	100	0
2	Peripatopsidae	100	0	100	0	100	0	100	0	100	0	100	0	100	0	100	0
3	Tardigrada	100	0	100	0	100	0	100	0	100	0	100	0	100	0	100	0
4	Arthropoda	100	0	100	0	100	0	100	0	100	0	100	0	100	0	100	0
5	Pycnogonida	100	0	100	0	100	0	100	0	100	0	100	0	100	0	100	0
6	Ammotheidae + Endeididae	86	-7	91	-1	90	-3	95	3	92	-1	96	3	91	-2	93	0
8	Ammotheidae	96	-3	87	-2	93	-6	83	-6	98	-1	92	3	98	-1	86	-3
9	Tanystylum + Achelia	93	-5	94	-3	90	-8	95	-2	93	-5	97	1	96	-2	97	0
12	Euchelicerata	100	0	100	0	100	0	100	0	100	0	100	0	100	0	100	0
13	Xiphosura	100	0	100	0	100	0	100	0	100	0	100	0	100	0	100	0
14	Arachnida	74	6	42	**25**	62	-6	33	**16**	60	-8	25	8	68	0	31	14
15	Pulmonata	33	**-32**	57	-15	42	**-23**	76	5	53	-12	74	3	62	-3	78	7
16	Scorpiones	100	0	100	0	100	0	100	0	100	0	100	0	100	0	100	0
17	Tetrapulmonata	97	-2	96	-1	100	1	99	2	99	0	99	2	99	0	99	1
18	Pedipalpi	100	0	97	-1	100	0	92	-6	100	0	95	-3	100	0	98	0
19	Uropygi	100	0	99	0	100	0	100	0	99	-1	99	0	100	0	100	0
20	Mandibulata	98	-1	97	-1	100	1	98	0	100	1	99	0	100	1	97	-1
21	Myriapoda	100	0	100	0	100	0	100	0	100	0	100	0	100	0	100	0
22	Chilopoda	100	0	100	0	100	0	100	0	100	0	100	0	100	0	100	0
23	Pleurostigmophora	97	4	95	3	98	5	97	5	97	4	94	2	96	3	96	4
24	Scolopendromorpha + Lithobiomorpha	87	-12	63	**-34**	96	-3	86	-11	99	0	96	-1	98	-1	91	-6
27	Diplopoda	100	1	95	-1	98	-1	94	-3	99	0	95	-2	99	0	97	1
28	Chilognatha	100	0	100	0	100	0	100	0	100	0	100	0	100	0	100	0
32	Edafopoda (=Symphyla + Pauropoda)	59	**-33**	66	2	52	**-40**	73	9	60	**-32**	68	4	59	**-33**	71	7
33	Symphyla	100	0	100	0	100	0	100	0	100	0	100	0	100	0	100	0
34	Pancrustacea	100	0	100	0	100	0	100	0	100	0	100	0	100	0	100	0
35	Oligostraca	98	-2	95	-3	98	-2	98	0	99	-1	97	-1	99	-1	98	0
37	Myodocopa	100	0	100	0	100	0	100	0	100	0	100	0	100	0	100	0
39	Ichthyostraca	100	0	100	0	100	0	100	0	100	0	100	0	100	0	100	0
40	Altocrustacea	32	**-61**	7	-13	51	**-42**	8	-12	55	**-38**	8	-12	52	**-41**	13	-7
41	Vericrustacea	26	**-60**	13	-11	47	**-39**	11	-13	41	**-45**	8	**-16**	53	**-33**	16	-7
42	Branchiopoda	100	0	100	0	100	0	100	0	100	0	100	0	100	0	100	0
43	Anostraca	100	0	100	0	100	0	100	0	100	0	100	0	100	0	100	0
44	Phyllopoda	100	0	100	0	100	0	100	0	100	0	100	0	100	0	100	0
45	Diplostraca	100	0	100	0	100	0	100	0	100	0	100	0	100	0	100	0
46	Cladocera + Spinicaudata	100	0	100	0	100	0	100	0	100	0	100	0	100	0	100	0
47	Multicrustacea	53	**-47**	29	**-23**	75	**-25**	34	**-18**	75	**-25**	40	-12	92	-8	47	-5
48	Copepoda	100	0	100	0	100	0	100	0	100	0	100	0	100	0	100	0
49	Cyclopoida	100	0	100	0	100	0	100	0	100	0	100	0	100	0	100	0
50	Communostraca	96	12	99	0	95	11	97	-1	90	6	97	-1	97	13	98	-1
51	Malacostraca	100	0	100	0	100	0	100	0	100	0	100	0	100	0	100	0
52	Eumalacostraca	100	0	100	0	100	0	100	0	100	0	100	0	100	0	100	0
53	Eucarida + Peracarida	80	-7	86	-2	79	-8	84	-3	81	-6	85	-2	85	-2	79	-8
54	Thecostraca	100	0	100	0	100	0	100	0	100	0	100	0	100	0	100	0
55	Thoracica	100	0	100	0	100	0	100	0	100	0	100	0	100	0	100	0
56	Sessilia	96	-1	98	1	96	-1	96	0	95	-2	98	2	93	-4	94	-3
57	Miracrustacea	27	**-67**	13	-3	38	**-56**	20	4	42	**-52**	15	-1	33	**-61**	4	-12
58	Xenocarida	52	**-41**	44	-11	68	**-25**	60	5	69	**-24**	73	**18**	61	**-32**	55	0
59	Hexapoda	100	0	100	0	100	0	100	0	100	0	100	0	100	0	100	0
60	Entognatha	34	**-52**	34	**-51**	40	**-46**	53	**-32**	71	**-15**	62	**-23**	50	**-36**	60	**-26**
61	Diplura	100	0	100	0	100	0	100	0	100	0	100	0	100	0	100	0
62	Collembola	100	0	100	0	100	0	100	0	100	0	100	0	100	0	100	0
63	Entomobryomorpha	99	1	98	1	100	2	98	2	100	2	99	2	99	1	97	0
64	Insecta	100	0	100	0	100	0	100	0	100	0	100	0	100	0	100	0
65	Archaeognatha	100	0	100	0	100	0	100	0	100	0	100	0	100	0	100	0
66	Dicondylia	100	0	100	0	100	0	100	0	100	0	100	0	100	0	100	0
67	Zygentoma	100	0	100	0	100	0	100	0	100	0	100	0	100	0	100	0
68	Pterygota	95	-4	97	-3	96	-3	97	-2	99	0	98	-2	95	-4	99	0
69	Paleoptera	65	-4	89	1	74	5	93	5	61	-8	87	-1	81	12	89	1
70	Ephemeroptera	100	0	100	0	100	0	100	0	100	0	100	0	100	0	100	0
71	Odonata	100	0	100	0	100	0	100	0	100	0	100	0	100	0	100	0
72	Neoptera	92	-5	94	-2	94	-3	95	-1	90	-7	92	-4	99	2	98	2
73	Polyneoptera	100	1	100	0	100	1	100	0	100	1	100	0	100	1	100	0
74	Blattodea + Orthoptera	93	-1	92	-3	89	-5	86	-9	82	-12	91	-4	94	0	97	2
75	Lepidoptera	100	0	100	0	100	0	100	0	100	0	100	0	100	0	100	0
76	Ditrysia	100	0	100	0	100	0	100	0	100	0	100	0	100	0	100	0

node index #	no non-co-Ser1, no non-co-Ser2				no non-co-Ser1				no non-co-Ser2				no non-Ser at co-Ser		split co-Ser: non-Ser/Ser	
	degen 1	Δ (no non-co-Ser1, no non-co-Ser2 - standard)	20AA	Δ (no non-co-Ser1, no non-co-Ser2 - standard)	degen 1	Δ (no non-co-Ser1 - standard)	20AA	Δ (no non-co-Ser1 - standard)	degen 1	Δ (no non-co-Ser2 - standard)	20AA	Δ (no non-co-Ser2 - standard)	degen 1	Δ (co-Ser1-Ser2-only - standard)	degen1	Δ (split no non-Ser at co-Ser / co-Ser - standard)
32	94	2	67	2	94	2	61	-3	92	0	64	0	88	-4	90	-2
40	88	-5	16	-4	90	-3	11	-9	90	-3	19	-1	93	0	95	2
41	78	-8	21	-3	82	-4	17	-7	84	-2	21	-2	85	-1	96	10
47	100	0	47	-5	100	0	43	-9	100	0	52	0	99	-1	100	0
57	91	-3	11	-5	93	-1	9	-7	93	-1	15	-1	84	-10	97	3
58	91	-2	46	-9	92	-1	41	-14	92	-1	57	2	65	**-28**	94	1
1	100	0	100	0	100	0	100	0	100	0	100	0	100	0	100	0
2	100	0	100	0	100	0	100	0	100	0	100	0	100	0	100	0
3	100	0	100	0	100	0	100	0	100	0	100	0	100	0	100	0
4	100	0	100	0	100	0	100	0	100	0	100	0	100	0	100	0
5	100	0	100	0	100	0	100	0	100	0	100	0	100	0	100	0
6	92	-1	93	1	93	0	93	1	93	0	93	1	85	-8	87	-6
8	99	0	93	4	99	0	91	1	99	0	93	4	91	-8	98	-1
9	99	1	97	1	100	2	97	0	98	0	98	1	94	-4	97	-1
12	100	0	100	0	100	0	100	0	100	0	100	0	100	0	100	0
13	100	0	100	0	100	0	100	0	100	0	100	0	100	0	100	0
14	72	4	26	9	65	-3	17	0	70	2	19	2	35	**-33**	65	-3
15	59	-6	59	-13	55	-10	59	-12	66	1	71	0	67	2	67	2
16	100	0	100	0	100	0	100	0	100	0	100	0	100	0	100	0
17	98	-1	93	-4	99	0	94	-3	98	-1	96	-1	100	1	100	1
18	100	0	98	0	100	0	99	1	100	0	98	0	100	0	100	0
19	100	0	100	0	100	0	100	0	100	0	100	0	95	-5	99	-1
20	99	0	98	0	100	1	98	0	99	0	98	-1	100	1	99	0
21	100	0	100	0	100	0	100	0	100	0	100	0	100	0	100	0
22	100	0	100	0	100	0	100	0	100	0	100	0	100	0	100	0
23	83	-10	86	-6	86	-7	86	-6	92	-1	93	1	98	5	98	5
24	98	-1	90	-7	97	-2	91	-6	100	1	98	1	86	-13	95	-4
27	100	1	99	2	99	0	98	2	99	0	99	2	94	-5	98	-1
28	100	0	100	0	100	0	100	0	100	0	100	0	100	0	100	0
32	94	2	67	2	94	2	61	-3	92	0	64	0	88	-4	90	-2
33	100	0	100	0	100	0	100	0	100	0	100	0	100	0	100	0
34	100	0	100	0	100	0	100	0	100	0	100	0	100	0	100	0
35	100	0	99	1	99	-1	97	-1	99	-1	98	0	94	-6	99	-1
37	100	0	100	0	100	0	100	0	100	0	100	0	100	0	100	0
39	100	0	100	0	100	0	100	0	100	0	100	0	100	0	100	0
40	88	-5	16	-4	90	-3	11	-9	90	-3	19	-1	93	0	95	2
41	78	-8	21	-3	82	-4	17	-7	84	-2	21	-2	85	-1	96	10
42	100	0	100	0	100	0	100	0	100	0	100	0	100	0	100	0
43	100	0	100	0	100	0	100	0	100	0	100	0	100	0	100	0
44	100	0	100	0	100	0	100	0	100	0	100	0	100	0	100	0
45	100	0	100	0	100	0	100	0	100	0	99	-1	100	0	100	0
46	100	0	100	0	100	0	100	0	100	0	100	0	100	0	100	0
47	100	0	47	-5	100	0	43	-9	100	0	52	0	99	-1	100	0
48	100	0	100	0	100	0	100	0	100	0	100	0	100	0	100	0
49	100	0	100	0	100	0	100	0	100	0	100	0	100	0	100	0
50	83	-1	97	-2	82	-2	98	0	85	1	97	-1	44	**-40**	76	-8
51	100	0	100	0	100	0	100	0	100	0	100	0	100	0	100	0
52	100	0	100	0	100	0	100	0	100	0	100	0	100	0	100	0
53	81	-6	87	0	82	-5	81	-6	86	-1	92	5	76	-11	85	-2
54	100	0	100	0	100	0	100	0	100	0	100	0	100	0	100	0
55	100	0	100	0	100	0	100	0	100	0	100	0	100	0	100	0
56	97	0	95	-2	94	-3	94	-3	98	1	95	-2	97	0	98	1
57	91	-3	11	-5	93	-1	9	-7	93	-1	15	-1	84	-10	97	3
58	91	-2	46	-9	92	-1	41	-14	92	-1	57	2	65	**-28**	94	1
59	100	0	100	0	100	0	100	0	100	0	100	0	100	0	100	0
60	80	-6	85	0	80	-6	86	1	83	-3	89	4	85	-1	66	**-20**
61	100	0	100	0	100	0	100	0	100	0	100	0	100	0	100	0
62	100	0	100	0	100	0	100	0	100	0	100	0	100	0	100	0
63	90	-8	93	-4	93	-5	97	0	94	-4	93	-4	99	1	99	1
64	100	0	100	0	100	0	100	0	100	0	100	0	100	0	100	0
65	100	0	100	0	100	0	100	0	100	0	100	0	100	0	100	0
66	100	0	100	0	100	0	100	0	100	0	100	0	100	0	100	0
67	100	0	100	0	100	0	100	0	100	0	100	0	100	0	100	0
68	100	1	99	0	100	1	98	-1	99	0	98	-1	98	-1	97	-2
69	61	-8	86	-2	58	-11	85	-3	69	0	89	1	53	**-16**	72	3
70	100	0	100	0	100	0	100	0	100	0	100	0	100	0	100	0
71	100	0	100	0	100	0	100	0	100	0	100	0	100	0	100	0
72	97	0	95	0	97	0	94	-1	97	0	94	-1	96	-1	91	-6
73	99	0	100	0	100	1	100	0	99	0	100	0	98	-1	100	1
74	95	1	97	2	94	0	97	2	96	2	92	-3	93	-1	92	-2
75	100	0	100	0	100	0	100	0	100	0	100	0	100	0	100	0
76	100	0	100	0	100	0	100	0	100	0	100	0	100	0	100	0

node index #	Ser1 to Ser2				Ser2 to Ser1				co-Ser1 to Phe				co-Ser1 to Trp				co-Ser1 to Tyr			
	degen 1	Δ (Ser1 to Ser2 - standard)	20AA	Δ (Ser1 to Ser2 - standard)	degen 1	Δ (Ser2 to Ser1 - standard)	20AA	Δ (Ser2 to Ser1 - standard)	degen 1	Δ (co-Ser1 to Phe - standard)	20AA	Δ (co-Ser1 to Phe - standard)	degen 1	Δ (co-Ser1 to Trp - standard)	20AA	Δ (co-Ser1 to Trp - standard)	degen 1	Δ (co-Ser1 to Tyr - standard)	20AA	Δ (co-Ser1 to Tyr - standard)
32	54	-38	72	8	66	-26	67	3	96	4	94	29	93	1	95	30	95	3	95	31
40	57	-36	20	1	63	-30	18	-1	91	-2	59	39	95	2	69	49	91	-2	65	45
41	40	-46	25	1	47	-39	21	-3	86	0	59	35	92	6	66	42	87	1	70	46
47	84	-16	56	4	90	-10	53	1	100	0	89	37	100	0	97	45	100	0	95	43
57	42	-52	15	-1	41	-53	13	-3	88	-6	65	49	91	-3	64	48	87	-7	67	51
58	59	-34	53	-2	58	-35	56	1	86	-7	84	29	90	-3	84	29	87	-6	83	28
1	100	0	100	0	100	0	100	0	100	0	100	0	100	0	100	0	100	0	100	0
2	100	0	100	0	100	0	100	0	100	0	100	0	100	0	100	0	100	0	100	0
3	100	0	100	0	100	0	100	0	100	0	100	0	100	0	100	0	100	0	100	0
4	100	0	100	0	100	0	100	0	100	0	100	0	100	0	100	0	100	0	100	0
5	100	0	100	0	100	0	100	0	100	0	100	0	100	0	100	0	100	0	100	0
6	82	-11	95	2	89	-4	97	4	89	-4	94	2	88	-5	95	2	88	-5	94	1
8	96	-3	91	2	99	0	91	2	99	0	92	2	99	0	92	2	98	-1	90	1
9	98	0	99	2	98	0	95	-2	98	0	98	1	98	0	96	-1	98	0	98	1
12	100	0	100	0	100	0	100	0	100	0	100	0	100	0	100	0	100	0	100	0
13	100	0	100	0	100	0	100	0	100	0	100	0	100	0	100	0	100	0	100	0
14	50	-18	16	-1	63	-5	16	-1	62	-6	14	-3	66	-2	25	8	60	-8	11	-6
15	82	17	73	2	67	2	73	1	67	2	77	6	66	1	84	12	61	-4	82	11
16	100	0	100	0	100	0	100	0	100	0	100	0	100	0	100	0	100	0	100	0
17	93	-6	97	-1	99	0	98	0	99	0	99	1	97	-2	95	-2	98	-1	94	-3
18	100	0	97	-1	100	0	97	-1	100	0	98	0	100	0	99	1	100	0	98	0
19	100	0	99	0	99	-1	100	0	100	0	100	0	100	0	100	0	100	0	100	0
20	99	0	99	0	100	1	99	0	98	-1	96	-2	97	-2	92	-7	96	-3	94	-5
21	100	0	100	0	100	0	100	0	100	0	100	0	100	0	99	-1	100	0	100	0
22	100	0	100	0	100	0	100	0	100	0	100	0	100	0	100	0	100	0	100	0
23	96	3	90	-2	95	2	91	-1	96	3	77	-15	95	2	73	-19	96	3	78	-14
24	100	1	97	0	100	1	98	1	98	-1	92	-5	100	1	89	-8	99	0	95	-2
27	99	0	97	1	100	1	97	1	99	0	99	3	100	1	97	1	99	0	97	1
28	100	0	100	0	100	0	100	0	100	0	100	0	100	0	100	0	100	0	100	0
32	54	-38	72	8	66	-26	67	3	96	4	94	29	93	1	95	30	95	3	95	31
33	100	0	100	0	100	0	100	0	100	0	100	0	100	0	100	0	100	0	100	0
34	100	0	100	0	100	0	100	0	100	0	100	0	100	0	100	0	100	0	100	0
35	99	-1	99	1	100	0	99	1	99	-1	99	1	99	-1	98	0	100	0	97	-1
37	100	0	100	0	100	0	100	0	100	0	100	0	100	0	100	0	100	0	100	0
39	100	0	100	0	100	0	100	0	100	0	100	0	100	0	100	0	100	0	100	0
40	57	-36	20	1	63	-30	18	-1	91	-2	59	39	95	2	69	49	91	-2	65	45
41	40	-46	25	1	47	-39	21	-3	86	0	59	35	92	6	66	42	87	1	70	46
42	100	0	100	0	100	0	100	0	100	0	100	0	100	0	100	0	100	0	100	0
43	100	0	100	0	100	0	100	0	100	0	100	0	100	0	100	0	100	0	100	0
44	100	0	100	0	100	0	100	0	100	0	100	0	100	0	100	0	100	0	100	0
45	100	0	100	0	100	0	100	0	100	0	100	0	100	0	99	-1	100	0	100	0
46	100	0	100	0	100	0	100	0	100	0	100	0	100	0	100	0	100	0	100	0
47	84	-16	56	4	90	-10	53	1	100	0	89	37	100	0	97	45	100	0	95	43
48	100	0	100	0	100	0	100	0	100	0	100	0	100	0	100	0	100	0	100	0
49	100	0	100	0	100	0	100	0	100	0	100	0	100	0	100	0	100	0	100	0
50	86	2	98	-1	98	14	98	-1	81	-3	86	-13	86	2	72	-27	77	-7	87	-11
51	100	0	100	0	100	0	100	0	100	0	100	0	100	0	100	0	100	0	100	0
52	100	0	100	0	100	0	100	0	100	0	100	0	100	0	100	0	100	0	100	0
53	91	4	89	1	79	-8	86	-1	70	-17	71	-16	77	-10	57	-30	76	-11	73	-14
54	100	0	100	0	100	0	100	0	100	0	100	0	100	0	100	0	100	0	100	0
55	100	0	100	0	100	0	100	0	100	0	100	0	100	0	100	0	100	0	100	0
56	96	-1	97	0	94	-3	97	1	90	-7	89	-7	90	-7	87	-10	90	-7	87	-9
57	42	-52	15	-1	41	-53	13	-3	88	-6	65	49	91	-3	64	48	87	-7	67	51
58	59	-34	53	-2	58	-35	56	1	86	-7	84	29	90	-3	84	29	87	-6	83	28
59	100	0	100	0	100	0	100	0	100	0	99	-1	100	0	95	-5	100	0	99	0
60	95	9	87	2	80	-6	86	1	88	2	79	-7	97	11	69	-16	89	3	87	2
61	100	0	100	0	100	0	100	0	100	0	100	0	100	0	100	0	100	0	100	0
62	100	0	100	0	100	0	100	0	100	0	100	0	100	0	100	0	100	0	100	0
63	99	1	97	0	100	2	98	1	99	1	94	-3	98	0	92	-5	98	0	92	-5
64	100	0	100	0	100	0	100	0	100	0	100	0	100	0	100	0	100	0	100	0
65	100	0	100	0	100	0	100	0	100	0	100	0	100	0	100	0	100	0	100	0
66	100	0	100	0	100	0	100	0	100	0	100	0	100	0	100	0	100	0	100	0
67	100	0	100	0	100	0	100	0	100	0	100	0	100	0	100	0	100	0	100	0
68	99	0	99	-1	97	-2	99	-1	98	-1	99	0	99	0	99	-1	99	0	98	-1
69	68	-1	89	0	84	15	91	3	54	-15	80	-8	58	-11	76	-12	61	-8	79	-9
70	100	0	100	0	100	0	100	0	100	0	100	0	100	0	100	0	100	0	100	0
71	100	0	100	0	100	0	100	0	100	0	100	0	100	0	100	0	100	0	100	0
72	97	0	94	-1	99	2	96	1	97	0	94	-1	98	1	92	-3	96	-1	93	-3
73	100	1	100	0	100	1	100	0	100	1	87	-12	100	1	69	-31	100	1	85	-15
74	95	1	95	0	97	3	94	-1	99	5	98	3	98	4	97	2	96	2	96	1
75	100	0	100	0	100	0	100	0	100	0	100	0	100	0	100	0	100	0	100	0
76	100	0	100	0	100	0	100	0	100	0	100	0	100	0	100	0	100	0	100	0

node index #	*co-Ser2 to Phe*				*co-Ser2 to Trp*				*co-Ser2 to Tyr*			
	degen 1	Δ (co-Ser2 to Phe - standard)	20AA	Δ (co-Ser2 to Phe - standard)	degen 1	Δ (co-Ser2 to Trp - standard)	20AA	Δ (co-Ser2 to Trp - standard)	degen 1	Δ (co-Ser2 to Tyr - standard)	20AA	Δ (co-Ser2 to Tyr - standard)
32	78	-14	94	30	76	-16	95	31	73	-19	93	29
40	88	-5	52	33	87	-6	67	47	86	-7	62	42
41	73	-13	56	32	86	0	69	46	77	-9	62	38
47	100	0	97	45	100	0	98	46	99	-1	97	45
57	82	-12	78	62	89	-5	80	64	78	-16	73	57
58	84	-9	91	36	88	-5	92	37	85	-8	90	35
1	100	0	100	0	100	0	100	0	100	0	100	0
2	100	0	100	0	100	0	100	0	100	0	100	0
3	100	0	100	0	100	0	100	0	100	0	100	0
4	100	0	99	-1	100	0	99	-1	100	0	99	-1
5	100	0	100	0	100	0	100	0	100	0	100	0
6	94	1	97	4	91	-2	96	3	96	3	98	5
8	99	0	95	6	100	1	96	6	99	0	97	7
9	96	-2	98	1	97	-1	95	-2	96	-2	96	-1
12	100	0	100	0	100	0	100	0	100	0	100	0
13	100	0	100	0	100	0	100	0	100	0	100	0
14	69	1	18	1	59	-9	24	7	64	-4	26	9
15	70	5	88	17	62	-3	82	11	66	1	86	15
16	100	0	100	0	100	0	100	0	100	0	100	0
17	98	-1	99	1	98	-1	99	2	98	-1	99	2
18	100	0	90	-8	100	0	87	-11	100	0	89	-9
19	100	0	100	0	100	0	100	0	100	0	100	0
20	99	0	98	0	100	1	99	0	100	1	97	-2
21	100	0	100	0	100	0	99	-1	100	0	100	0
22	100	0	100	0	100	0	100	0	100	0	100	0
23	93	0	82	-10	90	-3	81	-11	90	-3	85	-7
24	100	1	99	2	100	1	99	2	99	0	98	1
27	100	1	97	0	100	1	96	0	99	0	95	-1
28	100	0	100	0	100	0	100	0	100	0	100	0
32	78	-14	94	30	76	-16	95	31	73	-19	93	29
33	100	0	100	0	100	0	100	0	100	0	100	0
34	100	0	100	0	100	0	100	0	100	0	100	0
35	100	0	100	2	100	0	100	2	100	0	99	1
37	100	0	100	0	100	0	100	0	100	0	100	0
39	100	0	100	0	100	0	100	0	100	0	100	0
40	88	-5	52	33	87	-6	67	47	86	-7	62	42
41	73	-13	56	32	86	0	69	46	77	-9	62	38
42	100	0	100	0	100	0	100	0	100	0	100	0
43	100	0	100	0	100	0	100	0	100	0	100	0
44	100	0	100	0	100	0	100	0	100	0	100	0
45	100	0	100	0	100	0	100	0	100	0	100	0
46	100	0	100	0	100	0	100	0	100	0	100	0
47	100	0	97	45	100	0	98	46	99	-1	97	45
48	100	0	100	0	100	0	100	0	100	0	100	0
49	100	0	100	0	100	0	100	0	100	0	100	0
50	98	14	93	-5	94	10	83	-16	95	11	86	-12
51	100	0	100	0	100	0	100	0	100	0	100	0
52	100	0	100	0	100	0	100	0	100	0	100	0
53	85	-2	88	1	84	-3	90	3	79	-8	89	2
54	100	0	100	0	100	0	100	0	100	0	100	0
55	100	0	100	0	100	0	100	0	100	0	100	0
56	97	0	99	2	98	1	100	3	99	2	99	3
57	82	-12	78	62	89	-5	80	64	78	-16	73	57
58	84	-9	91	36	88	-5	92	37	85	-8	90	35
59	100	0	100	0	100	0	98	-2	100	0	100	0
60	68	-18	86	1	87	1	87	2	74	-12	86	1
61	100	0	100	0	100	0	100	0	100	0	100	0
62	100	0	100	0	100	0	100	0	100	0	100	0
63	95	-3	90	-6	96	-2	90	-6	99	1	92	-5
64	100	0	100	0	100	0	100	0	100	0	100	0
65	100	0	100	0	100	0	100	0	100	0	100	0
66	100	0	100	0	100	0	100	0	100	0	100	0
67	100	0	100	0	100	0	100	0	100	0	100	0
68	94	-5	96	-3	94	-5	98	-1	95	-4	97	-2
69	70	1	84	-5	73	4	81	-7	71	2	84	-4
70	100	0	100	0	100	0	100	0	100	0	100	0
71	100	0	100	0	100	0	100	0	100	0	100	0
72	98	1	93	-2	98	1	88	-8	99	2	93	-3
73	100	1	91	-8	100	1	89	-11	100	1	93	-6
74	95	1	97	2	95	1	96	1	95	1	96	1
75	100	0	100	0	100	0	100	0	100	0	100	0
76	100	0	100	0	100	0	100	0	100	0	100	0

node index #	Asp to Glu				Gln to Asn				Ile to Ala				Phe to Tyr				Val to Ala			
	degen 1	Δ (Asp to Glu - standard)	20AA	Δ (Asp to Glu - standard)	degen 1	Δ (Gln to Asn - standard)	20AA	Δ (Gln to Asn - standard)	degen 1	Δ (Ile to Ala - standard)	20AA	Δ (Ile to Ala - standard)	degen 1	Δ (Phe to Tyr - standard)	20AA	Δ (Phe to Tyr - standard)	degen 1	Δ (Val to Ala - standard)	20AA	Δ (Val to Ala - standard)
32	90	-2	62	-2	95	3	68	4	97	5	69	5	90	-2	58	-6	96	4	53	-12
40	88	-5	18	-2	95	2	19	-1	90	-3	20	1	95	2	17	-2	92	-1	20	0
41	74	-12	17	-7	92	6	19	-4	80	-6	17	-6	85	-1	18	-5	71	-15	31	7
47	100	0	59	7	100	0	35	**-17**	100	0	41	-11	100	0	47	-5	100	0	50	-2
57	97	3	13	-3	91	-3	11	-5	94	0	10	-6	92	-2	12	-4	95	1	26	10
58	96	3	52	-3	92	-1	47	-8	95	2	49	-6	91	-2	45	-10	90	-3	37	**-18**
1	100	0	100	0	100	0	100	0	100	0	100	0	100	0	100	0	100	0	100	0
2	100	0	100	0	100	0	100	0	100	0	100	0	100	0	100	0	100	0	100	0
3	100	0	100	0	100	0	100	0	100	0	100	0	100	0	100	0	100	0	100	0
4	100	0	100	0	100	0	100	0	100	0	100	0	100	0	100	0	100	0	100	0
5	100	0	100	0	100	0	100	0	100	0	100	0	100	0	100	0	100	0	100	0
6	94	1	96	4	95	2	97	5	96	3	96	4	95	2	94	2	95	2	94	1
8	99	0	88	-1	99	0	92	3	99	0	85	-5	100	1	93	3	95	-4	64	**-25**
9	99	1	98	1	98	0	96	0	100	2	96	-1	98	0	97	0	95	-3	88	-9
12	100	0	100	0	100	0	100	0	100	0	100	0	100	0	100	0	100	0	100	0
13	100	0	100	0	100	0	100	0	100	0	100	0	100	0	100	0	100	0	100	0
14	61	-7	10	-7	65	-3	29	12	84	**16**	25	8	53	-15	11	-6	82	14	28	11
15	64	-1	77	6	71	6	64	-7	55	-10	58	-13	64	-1	79	8	59	-6	81	10
16	100	0	100	0	100	0	100	0	100	0	100	0	100	0	100	0	100	0	100	0
17	99	0	96	-2	99	0	98	0	99	0	97	-1	99	0	98	1	99	0	93	-4
18	100	0	96	-2	100	0	96	-2	100	0	99	1	100	0	96	-2	100	0	97	-1
19	100	0	100	1	100	0	100	0	100	0	99	0	100	0	100	1	99	-1	99	-1
20	99	0	98	0	98	-1	97	-2	100	1	99	1	100	1	99	1	99	0	99	0
21	100	0	100	0	100	0	100	0	100	0	100	0	100	0	100	0	100	0	100	0
22	100	0	100	0	100	0	100	0	100	0	100	0	100	0	100	0	100	0	100	0
23	93	0	92	0	89	-4	88	-4	99	6	84	-8	92	-1	90	-2	93	0	88	-4
24	100	1	96	-1	99	0	99	2	99	0	98	1	100	1	98	1	100	1	98	1
27	99	0	95	-1	99	0	99	3	100	1	99	3	100	1	96	0	100	1	97	1
28	100	0	100	0	100	0	100	0	100	0	100	0	100	0	100	0	100	0	100	0
32	90	-2	62	-2	95	3	68	4	97	5	69	5	90	-2	58	-6	96	4	53	-12
33	100	0	100	0	100	0	100	0	100	0	100	0	100	0	100	0	100	0	100	0
34	100	0	100	0	100	0	100	0	100	0	100	0	100	0	100	0	100	0	100	0
35	98	-2	98	0	98	-2	99	1	100	0	98	0	99	-1	99	1	100	0	95	-3
37	100	0	100	0	100	0	100	0	100	0	100	0	100	0	100	0	100	0	100	0
39	100	0	100	0	100	0	100	0	100	0	100	0	100	0	100	0	100	0	100	0
40	88	-5	18	-2	95	2	19	-1	90	-3	20	1	95	2	17	-2	92	-1	20	0
41	74	-12	17	-7	92	6	19	-4	80	-6	17	-6	85	-1	18	-5	71	-15	31	7
42	100	0	100	0	100	0	100	0	100	0	100	0	100	0	100	0	100	0	100	0
43	100	0	100	0	100	0	100	0	100	0	100	0	100	0	100	0	100	0	100	0
44	100	0	100	0	100	0	100	0	100	0	100	0	100	0	100	0	100	0	100	0
45	100	0	100	0	100	0	100	0	100	0	100	0	100	0	100	0	100	0	98	-2
46	100	0	100	0	100	0	100	0	100	0	100	0	100	0	100	0	100	0	100	0
47	100	0	59	7	100	0	35	**-17**	100	0	41	-11	100	0	47	-5	100	0	50	-2
48	100	0	100	0	100	0	100	0	100	0	100	0	100	0	100	0	100	0	100	0
49	100	0	100	0	100	0	100	0	100	0	100	0	100	0	100	0	100	0	100	0
50	88	4	96	-3	89	5	98	-1	88	4	97	-1	77	-7	97	-2	78	-6	97	-2
51	100	0	100	0	100	0	100	0	100	0	100	0	100	0	100	0	100	0	100	0
52	100	0	100	0	100	0	100	0	100	0	100	0	100	0	100	0	100	0	100	0
53	77	-10	63	**-24**	90	3	86	-1	85	-2	85	-2	81	-6	81	-6	92	5	92	5
54	100	0	100	0	100	0	100	0	100	0	100	0	100	0	100	0	100	0	100	0
55	100	0	100	0	100	0	100	0	100	0	100	0	100	0	100	0	100	0	100	0
56	97	0	96	0	97	0	97	0	99	2	96	0	95	-2	93	-4	95	-2	91	-5
57	97	3	13	-3	91	-3	11	-5	94	0	10	-6	92	-2	12	-4	95	1	26	10
58	96	3	52	-3	92	-1	47	-8	95	2	49	-6	91	-2	45	-10	90	-3	37	**-18**
59	100	0	100	0	100	0	99	0	99	-1	100	0	100	0	100	0	99	-1	99	-1
60	78	-8	86	1	64	**-22**	83	-2	79	-7	82	-3	77	-9	85	0	69	**-17**	81	-4
61	100	0	100	0	100	0	100	0	100	0	100	0	100	0	100	0	100	0	100	0
62	100	0	100	0	100	0	100	0	100	0	100	0	100	0	100	0	100	0	100	0
63	98	0	97	1	95	-3	96	-1	98	0	95	-2	90	-8	88	-9	99	1	97	0
64	100	0	100	0	100	0	100	0	100	0	100	0	100	0	100	0	100	0	100	0
65	100	0	100	0	100	0	100	0	100	0	100	0	100	0	100	0	100	0	100	0
66	100	0	100	0	100	0	100	0	100	0	100	0	100	0	100	0	100	0	100	0
67	100	0	100	0	100	0	100	0	100	0	100	0	100	0	100	0	100	0	100	0
68	99	0	99	0	99	0	98	-1	91	-8	95	-5	98	-1	99	-1	95	-4	97	-3
69	71	2	89	0	60	-9	87	-1	78	9	84	-4	73	4	90	1	75	6	86	-2
70	100	0	100	0	100	0	100	0	100	0	100	0	100	0	100	0	100	0	100	0
71	100	0	100	0	100	0	100	0	100	0	100	0	100	0	100	0	100	0	100	0
72	96	-1	94	-2	95	-2	97	2	91	-6	87	-9	94	-3	93	-3	94	-3	94	-2
73	99	0	100	0	99	0	100	0	100	1	100	0	99	0	99	-1	100	1	100	0
74	96	2	98	3	98	4	95	0	98	4	93	-2	92	-2	91	-4	98	4	97	2
75	100	0	100	0	100	0	100	0	100	0	100	0	100	0	100	0	100	0	100	0
76	100	0	100	0	100	0	100	0	100	0	100	0	100	0	100	0	100	0	100	0

node index #	no Phe				no Trp				no Tyr			
	degen 1	Δ (no Phe - standard)	20AA	Δ (no Phe - standard)	degen 1	Δ (no Trp - standard)	20AA	Δ (no Trp - standard)	degen 1	Δ (no Tyr - standard)	20AA	Δ (no Tyr - standard)
32	92	0	60	-4	90	-2	66	2	90	-2	60	-4
40	89	-4	19	0	94	1	26	6	92	-1	17	-3
41	83	-3	20	-4	89	3	31	7	86	0	18	-6
47	100	0	56	4	100	0	63	11	100	0	47	-5
57	94	0	11	-5	95	1	18	2	95	1	10	-6
58	92	-1	53	-2	93	0	56	1	92	-1	58	3
1	100	0	100	0	100	0	100	0	100	0	100	0
2	100	0	100	0	100	0	100	0	100	0	100	0
3	100	0	100	0	100	0	100	0	100	0	100	0
4	100	0	100	0	100	0	100	0	100	0	100	0
5	100	0	100	0	100	0	100	0	100	0	100	0
6	92	-1	93	1	94	1	95	2	84	-9	89	-4
8	100	1	96	7	99	0	92	2	99	0	88	-2
9	97	-1	96	-1	98	0	98	1	98	0	95	-1
12	100	0	100	0	100	0	100	0	100	0	100	0
13	100	0	100	0	100	0	100	0	100	0	100	0
14	61	-7	17	0	70	2	17	0	52	**-16**	12	-5
15	68	3	74	3	67	2	75	4	68	3	79	8
16	100	0	100	0	100	0	100	0	100	0	100	0
17	99	0	98	0	100	1	98	1	99	0	98	0
18	100	0	96	-2	100	0	97	-1	100	0	98	0
19	100	0	98	-1	100	0	100	0	100	0	99	0
20	98	-1	98	-1	99	0	98	0	97	-2	97	-1
21	100	0	100	0	100	0	100	0	100	0	100	0
22	100	0	100	0	100	0	100	0	100	0	100	0
23	93	0	90	-2	92	-1	92	0	93	0	91	-1
24	100	1	97	0	100	1	98	1	100	1	97	0
27	98	-1	94	-3	99	0	97	1	98	-1	96	-1
28	100	0	100	0	100	0	100	0	100	0	100	0
32	92	0	60	-4	90	-2	66	2	90	-2	60	-4
33	100	0	100	0	100	0	100	0	100	0	100	0
34	100	0	100	0	100	0	100	0	100	0	100	0
35	98	-2	98	0	99	-1	99	1	99	-1	98	0
37	100	0	100	0	100	0	100	0	100	0	100	0
39	100	0	100	0	100	0	100	0	100	0	100	0
40	89	-4	19	0	94	1	26	6	92	-1	17	-3
41	83	-3	20	-4	89	3	31	7	86	0	18	-6
42	100	0	100	0	100	0	100	0	100	0	100	0
43	100	0	100	0	100	0	100	0	100	0	100	0
44	100	0	100	0	100	0	100	0	100	0	100	0
45	100	0	100	0	100	0	100	0	100	0	100	0
46	100	0	100	0	100	0	100	0	100	0	100	0
47	100	0	56	4	100	0	63	11	100	0	47	-5
48	100	0	100	0	100	0	100	0	100	0	100	0
49	100	0	100	0	100	0	100	0	100	0	100	0
50	73	-11	96	-3	80	-4	98	-1	72	-12	96	-3
51	100	0	100	0	100	0	100	0	100	0	100	0
52	100	0	100	0	100	0	100	0	100	0	100	0
53	83	-4	81	-7	83	-4	84	-4	86	-1	84	-3
54	100	0	100	0	100	0	100	0	100	0	100	0
55	100	0	100	0	100	0	100	0	100	0	100	0
56	91	-6	88	-9	95	-2	94	-2	95	-2	93	-3
57	94	0	11	-5	95	1	18	2	95	1	10	-6
58	92	-1	53	-2	93	0	56	1	92	-1	58	3
59	100	0	100	0	100	0	100	0	100	0	100	0
60	86	0	91	6	85	-1	87	2	87	1	93	8
61	100	0	100	0	100	0	100	0	100	0	100	0
62	100	0	100	0	100	0	100	0	100	0	100	0
63	90	-8	90	-6	97	-1	94	-2	94	-4	85	-11
64	100	0	100	0	100	0	100	0	100	0	100	0
65	100	0	100	0	100	0	100	0	100	0	100	0
66	100	0	100	0	100	0	100	0	100	0	100	0
67	100	0	100	0	100	0	100	0	100	0	100	0
68	97	-2	98	-2	98	-1	99	0	99	0	99	-1
69	69	0	90	2	71	2	88	-1	62	-7	87	-1
70	100	0	100	0	100	0	100	0	100	0	100	0
71	100	0	100	0	100	0	100	0	100	0	100	0
72	96	-1	95	0	97	0	95	-1	94	-3	95	0
73	99	0	99	0	99	0	100	0	98	-1	99	-1
74	91	-3	93	-2	94	0	95	0	86	-8	88	-7
75	100	0	100	0	100	0	100	0	100	0	100	0
76	100	0	100	0	100	0	100	0	100	0	100	0
