# Supplementary material for: Resolving Discrepancy between Nucleotides and Amino Acids in Deep-Level Arthropod Phylogenomics: Differentiating Serine Codons in 21-Amino-Acid Models
Source: PLoS One. 2012 Nov 20;7(11):e47450. doi: 10.1371/journal.pone.0047450 (PMC3502419; doi:10.1371/journal.pone.0047450)
Supplement: Table S4 — Individual substitution rates of the ECM model categorized by their effect on amino acids: synonymous, synonymous SER (S/Z) and nonsynonymous. (PDF) [file pone.0047450.s011.pdf]

Table S4: Individual substitution rates of the ECM model categorized by their effect on amino acids: synonymous, synonymous SER (S/Z) and nonsynonymous.

codon (AA) substitution	ECM rate		
	synonymous	synonymous SER (S/Z)	nonsynonymous
**AVERAGE**	**17.8281698734**	**5.094309375**	**0.480768556**
GGG (G) / CTA (L)			0.000000
AAA (K) / CTG (L)			0.000035
GCC (A) / ATT (I)			0.000040
GAT (D) / ATC (I)			0.000042
GCC (A) / AAT (N)			0.000045
AGT (Z) / TTC (F)			0.000047
ATA (I) / CAG (Q)			0.000048
GGC (G) / TAT (Y)			0.000058
GGA (G) / CTC (L)			0.000060
GAC (D) / TTA (L)			0.000062
CGC (R) / TTG (L)			0.000064
GGG (G) / CTT (L)			0.000066
GCA (A) / CGC (R)			0.000068
GGA (G) / CTG (L)			0.000070
GGG (G) / ATA (I)			0.000078
GGG (G) / ATT (I)			0.000081
GTA (V) / CCG (P)			0.000082
GGA (G) / ATC (I)			0.000084
GGC (G) / TCT (S)			0.000085
GGC (G) / CCA (P)			0.000090
GGT (G) / CAC (H)			0.000091
GCC (A) / TTT (F)			0.000093
AAT (N) / TTC (F)			0.000094
GGC (G) / CCT (P)			0.000096
GTC (V) / AGT (Z)			0.000097
GTG (V) / AGA (R)			0.000103
AGT (Z) / CTG (L)			0.000105
GGA (G) / ACC (T)			0.000112
GTA (V) / TCG (S)			0.000115
GTC (V) / TAT (Y)			0.000116
ATC (I) / CGT (R)			0.000118
GGG (G) / CTC (L)			0.000119
GGT (G) / TAC (Y)			0.000120
GCA (A) / CGG (R)			0.000123
GCC (A) / CTT (L)			0.000132
GAT (D) / TGC (C)			0.000135
GAG (E) / CTA (L)			0.000136
GAA (E) / CTG (L)			0.000140
GGT (G) / TTC (F)			0.000141
GTA (V) / CAG (Q)			0.000145
CAC (H) / TGT (C)			0.000155
GGT (G) / ATA (I)			0.000156

codon (AA) substitution	ECM rate synonymous	synonymous SER (S/Z)	nonsynonymous
GAC (D) / ACT (T)			0.000158
GGA (G) / CCG (P)			0.000164
GGT (G) / CTG (L)			0.000175
AGT (Z) / TAC (Y)			0.000180
GTT (V) / TCC (S)			0.000186
GGA (G) / ACG (T)			0.000187
GGC (G) / TTG (L)			0.000191
GTA (V) / CCC (P)			0.000192
GTG (V) / AAA (K)			0.000193
GTC (V) / AGA (R)			0.000205
GTT (V) / AGC (Z)			0.000219
GCT (A) / TTC (F)			0.000234
GAC (D) / CGT (R)			0.000236
GTT (V) / CCG (P)			0.000246
AGG (R) / TTA (L)			0.000247
GCT (A) / ATC (I)			0.000253
GAC (D) / TCT (S)			0.000254
ACG (T) / CTT (L)			0.000264
CCT (P) / TGC (C)			0.000270
GGT (G) / CTA (L)			0.000272
GTA (V) / TCC (S)			0.000279
GGA (G) / ATT (I)			0.000283
GGT (G) / CCC (P)			0.000288
GGA (G) / CAG (Q)			0.000289
GGG (G) / ATC (I)			0.000295
CTT (L) / TAC (Y)			0.000299
ATC (I) / TGT (C)			0.000311
GGA (G) / CTT (L)			0.000330
ATC (I) / TCG (S)			0.000344
GCA (A) / CTG (L)			0.000351
GTA (V) / CGT (R)			0.000353
GCA (A) / CTC (L)			0.000357
GTT (V) / TAC (Y)			0.000359
GAT (D) / CGG (R)			0.000368
GGT (G) / ACG (T)			0.000374
GTC (V) / CAT (H)			0.000375
CCC (P) / TTG (L)			0.000382
GGA (G) / CCC (P)			0.000384
ATT (I) / TCA (S)			0.000386
GAC (D) / AGT (Z)			0.000388
ACC (T) / CTT (L)			0.000396
ATT (I) / TGC (C)			0.000405
ACG (T) / CTA (L)			0.000408
GAG (E) / AGA (R)			0.000410
GCC (A) / CGA (R)			0.000416
GCT (A) / CTC (L)			0.000417
GCT (A) / TAC (Y)			0.000419
ATA (I) / TCT (S)			0.000424

codon (AA) substitution	ECM rate synonymous	synonymous SER (S/Z)	nonsynonymous
AGC (Z) / TTA (L)			0.000432
GGG (G) / ACC (T)			0.000447
ATC (I) / CCA (P)			0.000448
AGT (Z) / CAC (H)			0.000453
AAA (K) / TCG (S)			0.000459
GCC (A) / TGT (C)			0.000466
CAC (H) / TTT (F)			0.000467
ATC (I) / CAT (H)			0.000469
CCT (P) / TAC (Y)			0.000479
GCG (A) / AAA (K)			0.000482
GCC (A) / AGT (Z)			0.000485
GAT (D) / CTG (L)			0.000491
GTA (V) / AAG (K)			0.000524
GGT (G) / ACA (T)			0.000534
GTC (V) / CCA (P)			0.000537
GGG (G) / ACT (T)			0.000553
GAT (D) / ACC (T)			0.000559
GCC (A) / CAT (H)			0.000562
CGT (R) / TCA (S)			0.000579
GCA (A) / ATC (I)			0.000589
CAC (H) / TCT (S)			0.000593
ACG (T) / CTC (L)			0.000595
ACT (T) / TAC (Y)			0.000599
GGG (G) / ACA (T)			0.000610
GCG (A) / CGA (R)			0.000624
GTG (V) / AAT (N)			0.000628
GTA (V) / AGC (Z)			0.000657
CTT (L) / TGC (C)			0.000674
CTA (L) / TCT (S)			0.000678
AAG (K) / CTA (L)			0.000679
GTG (V) / CCA (P)			0.000717
GAC (D) / AGA (R)			0.000718
GGT (G) / TTA (L)			0.000741
GGA (G) / CGC (R)			0.000753
GAT (D) / CCC (P)			0.000769
GCT (A) / CTG (L)			0.000772
ACC (T) / TGT (C)			0.000776
GGC (G) / AGA (R)			0.000821
GTG (V) / CGA (R)			0.000832
GAG (E) / ACA (T)			0.000838
GGC (G) / CAT (H)			0.000843
ACA (T) / CTT (L)			0.000857
GAA (E) / CGG (R)			0.000858
GCG (A) / CTC (L)			0.000893
AAT (N) / TCC (S)			0.000929
AAC (N) / TGT (C)			0.000932
CGG (R) / TTT (F)			0.000934
AGG (R) / CTA (L)			0.000951

codon (AA) substitution	ECM rate synonymous	synonymous SER (S/Z)	nonsynonymous
AGG (R) / CTC (L)			0.000952
AAC (N) / CCT (P)			0.000959
AAT (N) / CGG (R)			0.000981
CTG (L) / TCC (S)			0.001022
GTC (V) / ACT (T)			0.001027
GAG (E) / CGA (R)			0.001040
ACC (T) / CGT (R)			0.001060
GAG (E) / TCA (S)			0.001061
AGT (Z) / CCG (P)			0.001066
GGA (G) / TTG (L)			0.001082
GTC (V) / TGT (C)			0.001087
GGC (G) / AAT (N)			0.001122
ACC (T) / CAT (H)			0.001125
GTA (V) / CCT (P)			0.001151
GGG (G) / AAA (K)			0.001158
GCG (A) / CAA (Q)			0.001165
GAT (D) / ATA (I)			0.001171
CGT (R) / TCC (S)			0.001208
AGC (Z) / CCT (P)			0.001247
ATC (I) / CGA (R)			0.001248
GGG (G) / CCA (P)			0.001254
GTC (V) / TCG (S)			0.001261
ACA (T) / CAG (Q)			0.001302
GGT (G) / CCG (P)			0.001312
GCT (A) / AAC (N)			0.001329
GTA (V) / TCT (S)			0.001356
GTG (V) / AGT (Z)			0.001358
GAC (D) / CTA (L)			0.001359
CGT (R) / TAC (Y)			0.001377
GTG (V) / TCC (S)			0.001394
GTG (V) / CGT (R)			0.001413
GGG (G) / CCC (P)			0.001441
ACT (T) / TGC (C)			0.001483
CGA (R) / TTG (L)			0.001527
GCG (A) / CGT (R)			0.001531
GTG (V) / CCC (P)			0.001537
GGC (G) / GTA (V)			0.001557
GCC (A) / TTG (L)			0.001654
CGC (R) / CCA (P)			0.001702
AGA (R) / TTG (L)			0.001718
GGA (G) / TCG (S)			0.001720
GGA (G) / AAG (K)			0.001760
GCC (A) / AGG (R)			0.001768
GAG (E) / CCA (P)			0.001791
GCT (A) / ATA (I)			0.001795
GCT (A) / CGA (R)			0.001871
GGG (G) / CCT (P)			0.001918
GTC (V) / AAA (K)			0.001930

codon (AA) substitution	ECM rate		
	synonymous	synonymous SER (S/Z)	nonsynonymous
GGA (G) / TCC (S)			0.001951
GTA (V) / ACC (T)			0.001957
ATA (I) / CCT (P)			0.002014
AGA (R) / TCC (S)			0.002044
GGC (G) / GTT (V)			0.002050
GTA (V) / AGG (R)			0.002062
ACC (T) / CGA (R)			0.002079
CAG (Q) / TCA (S)			0.002122
CAT (H) / TCC (S)			0.002137
AAA (K) / CTC (L)			0.002143
CAC (H) / TTG (L)			0.002163
GAA (E) / TCG (S)			0.002179
GCA (A) / CAG (Q)			0.002219
GCA (A) / ATT (I)			0.002262
GTA (V) / AAC (N)			0.002362
GCA (A) / CTT (L)			0.002374
CGC (R) / CCT (P)			0.002398
GGA (G) / ACT (T)			0.002449
GGT (G) / CCA (P)			0.002508
GTC (V) / ACA (T)			0.002515
GTA (V) / ACG (T)			0.002528
ATT (I) / CCA (P)			0.002597
CTG (L) / TGT (C)			0.002639
GAA (E) / CTC (L)			0.002679
AAT (N) / CCG (P)			0.002705
AGC (Z) / CTA (L)			0.002718
CCA (P) / TGT (C)			0.002795
GGG (G) / TCT (S)			0.002797
GTA (V) / CAC (H)			0.002808
AAC (N) / TTA (L)			0.002840
ACG (T) / CAA (Q)			0.002854
GGC (G) / AAA (K)			0.002863
ACG (T) / CGA (R)			0.002911
GGG (G) / TCC (S)			0.002974
ACC (T) / TTG (L)			0.002990
GTT (V) / ACC (T)			0.003020
GGG (G) / CAA (Q)			0.003029
CGT (R) / TTG (L)			0.003054
ATC (I) / CGG (R)			0.003064
AAC (N) / CGT (R)			0.003180
CGA (R) / TCG (S)			0.003211
GTG (V) / CGC (R)			0.003287
GAG (E) / ATT (I)			0.003312
GAC (D) / ACA (T)			0.003354
GGG (G) / TCA (S)			0.003376
GGT (G) / CAG (Q)			0.003377
AAC (N) / TCT (S)			0.003390
GAA (E) / ACG (T)			0.003464

codon (AA) substitution	ECM rate		
	synonymous	synonymous SER (S/Z)	nonsynonymous
CCT (P) / TTG (L)			0.003499
GGA (G) / CCT (P)			0.003549
GTG (V) / ACT (T)			0.003635
GAA (E) / TGC (C)			0.003641
GAC (D) / CCA (P)			0.003672
CGG (R) / TAT (Y)			0.003713
GAT (D) / TCC (S)			0.003717
GTT (V) / CGA (R)			0.003743
GCG (A) / AAT (N)			0.003771
AGG (R) / CAA (Q)			0.003786
ATT (I) / CAG (Q)			0.003859
GGT (G) / TTG (L)			0.003881
CCG (P) / TTT (F)			0.004063
GCT (A) / CTA (L)			0.004077
CGA (R) / TCC (S)			0.004089
GAC (D) / GTT (V)			0.004101
GTG (V) / AGC (Z)			0.004160
CCG (P) / TAT (Y)			0.004177
GAG (E) / CTT (L)			0.004220
GTT (V) / TGC (C)			0.004316
ACT (T) / CTA (L)			0.004348
GCA (A) / AAG (K)			0.004382
GAA (E) / AGG (R)			0.004420
AAG (K) / TCA (S)			0.004438
CCG (P) / TGC (C)			0.004585
ACG (T) / CGT (R)			0.004593
GCG (A) / ATC (I)			0.004629
GGT (G) / CGC (R)			0.004656
AGA (R) / CTT (L)			0.004747
CTG (L) / TAT (Y)			0.004815
CGG (R) / TCC (S)			0.004832
GTT (V) / ACG (T)			0.004868
AAA (K) / TTG (L)			0.004963
GGA (G) / CAC (H)			0.004982
GTC (V) / CGG (R)			0.005026
AGC (Z) / CAT (H)			0.005060
AGC (Z) / CCA (P)			0.005195
GCT (A) / AGC (Z)			0.005328
GAA (E) / CCG (P)			0.005410
CGC (R) / CTA (L)			0.005436
GAA (E) / TTG (L)			0.005472
GCC (A) / AAA (K)			0.005500
CCA (P) / TTG (L)			0.005535
AGC (Z) / TTG (L)			0.005790
GCG (A) / TTT (F)			0.005884
GGT (G) / AAC (N)			0.005905
GTC (V) / TTA (L)			0.005990
AAA (K) / TTC (F)			0.005996

codon (AA) substitution	ECM rate synonymous	synonymous SER (S/Z)	nonsynonymous
CGA (R) / TCT (S)			0.006103
GTA (V) / AGT (Z)			0.006305
GCG (A) / AGT (Z)			0.006402
GGC (G) / ACG (T)			0.006459
GAA (E) / TTC (F)			0.006464
CCA (P) / TTC (F)			0.006558
GTC (V) / CAA (Q)			0.006757
GAC (D) / GTA (V)			0.006776
GAC (D) / TTG (L)			0.006808
GAT (D) / CGA (R)			0.007070
GGT (G) / TCG (S)			0.007109
GAT (D) / GTC (V)			0.007112
GAC (D) / TCA (S)			0.007428
CAA (Q) / TTG (L)			0.007508
GGC (G) / TGT (C)			0.007608
GAG (E) / CGT (R)			0.007891
GGG (G) / TAT (Y)			0.007948
CCG (P) / CTT (L)			0.007978
ACG (T) / TTT (F)			0.008032
ATG (M) / CCC (P)			0.008455
GTA (V) / AAT (N)			0.008844
GCT (A) / TTA (L)			0.008892
GGC (G) / CGA (R)			0.008942
GAC (D) / AGG (R)			0.008986
GTA (V) / ACT (T)			0.009008
GAA (E) / ATC (I)			0.009174
GTT (V) / CCA (P)			0.009225
AGG (R) / TCA (S)			0.009261
AGT (Z) / TTG (L)			0.009289
AGT (Z) / CTA (L)			0.009512
AGG (R) / CTT (L)			0.009560
GTT (V) / CAG (Q)			0.009600
GTG (V) / TAT (Y)			0.009630
CAG (Q) / TTT (F)			0.009760
GGA (G) / GTC (V)			0.009773
CGG (R) / CCT (P)			0.009880
GAA (E) / TGG (W)			0.009966
GAC (D) / CGA (R)			0.009981
GAC (D) / TAT (Y)			0.010037
GAG (E) / CTC (L)			0.010061
CTT (L) / TGG (W)			0.010122
TCG (S) / TTA (L)			0.010188
CCA (P) / TGC (C)			0.010250
GTC (V) / ACG (T)			0.010298
CGG (R) / CCA (P)			0.010300
GGA (G) / CGG (R)			0.010419
ACA (T) / TTC (F)			0.010539
ATC (I) / CAA (Q)			0.010543

codon (AA) substitution	ECM rate		
	synonymous	synonymous SER (S/Z)	nonsynonymous
AGT (Z) / TTA (L)			0.010929
GAG (E) / TTT (F)			0.010974
AAA (K) / CGG (R)			0.011032
ACA (T) / TTG (L)			0.011134
GAC (D) / ATG (M)			0.011379
GTG (V) / ACA (T)			0.011433
ACT (T) / CCG (P)			0.011476
AAG (K) / TGT (C)			0.011489
GAT (D) / GTG (V)			0.011491
AGA (R) / CCT (P)			0.011511
GTT (V) / CTG (L)			0.011580
TCC (S) / TTA (L)			0.011609
GGT (G) / TCA (S)			0.011673
GCT (A) / TGC (C)			0.011733
GGT (G) / CGG (R)			0.011768
CTA (L) / TAC (Y)			0.011978
AAG (K) / CCA (P)			0.012002
GGA (G) / TCT (S)			0.012121
ATA (I) / TGC (C)			0.012138
GGC (G) / AGG (R)			0.012227
AGA (R) / ATC (I)			0.012331
GAT (D) / CTA (L)			0.012366
AAA (K) / CCC (P)			0.012394
ACT (T) / TTA (L)			0.012473
GAT (D) / AGC (Z)			0.012481
AGA (R) / TTC (F)			0.012600
CGC (R) / TGT (C)			0.012886
GCC (A) / CCA (P)			0.013077
AAG (K) / TTT (F)			0.013122
GAG (E) / TGC (C)			0.013216
GCT (A) / CCG (P)			0.013526
GTG (V) / AAC (N)			0.013582
GCT (A) / AGA (R)			0.013852
GCG (A) / CCT (P)			0.014101
ACG (T) / CGC (R)			0.014243
GGG (G) / TAC (Y)			0.014314
AAT (N) / ATC (I)			0.014351
CCT (P) / CTG (L)			0.014388
GAG (E) / TGT (C)			0.014439
CAC (H) / TCA (S)			0.014470
AAC (N) / CTA (L)			0.014540
ACA (T) / CCG (P)			0.015001
CGT (R) / CTG (L)			0.015019
GAT (D) / GCC (A)			0.015140
GGA (G) / CGT (R)			0.015310
GAT (D) / TAC (Y)			0.015452
ATG (M) / CGA (R)			0.015596
ACT (T) / TTG (L)			0.015652

codon (AA) substitution	ECM rate synonymous	synonymous SER (S/Z)	nonsynonymous
GAC (D) / CTG (L)			0.015686
GAT (D) / AGG (R)			0.015763
CGA (R) / TTC (F)			0.016020
CGC (R) / CTT (L)			0.016022
GGC (G) / CAA (Q)			0.016193
CCG (P) / CTA (L)			0.016442
GCA (A) / TTC (F)			0.016535
CCA (P) / CTC (L)			0.016609
CAA (Q) / TTC (F)			0.016628
AAC (N) / CGA (R)			0.016636
CTA (L) / TAT (Y)			0.016650
ACC (T) / ATA (I)			0.016701
GGT (G) / GTC (V)			0.016776
GGA (G) / GTG (V)			0.016839
CTA (L) / TGG (W)			0.017106
AGC (Z) / CTG (L)			0.017300
ACC (T) / CGG (R)			0.017774
GCG (A) / CCA (P)			0.017824
AGT (Z) / CGC (R)			0.018146
GGA (G) / TTC (F)			0.018315
GGC (G) / ATG (M)			0.018603
GAC (D) / GCT (A)			0.018617
AGT (Z) / ATC (I)			0.018727
GGG (G) / TTT (F)			0.018773
GCC (A) / CAA (Q)			0.018814
GCA (A) / TTG (L)			0.019406
AAT (N) / CTA (L)			0.019568
GTA (V) / CAT (H)			0.019586
GCC (A) / ACA (T)			0.019665
CGT (R) / TGG (W)			0.019696
GCC (A) / CGG (R)			0.020471
GTA (V) / CTG (L)			0.020529
CTA (L) / TGT (C)			0.020649
AAC (N) / ATA (I)			0.020837
AAA (K) / TCC (S)			0.020909
AGT (Z) / CCA (P)			0.021048
GGC (G) / CGT (R)			0.021435
CCG (P) / TCT (S)			0.021444
AGC (Z) / ATA (I)			0.021539
CTA (L) / TGC (C)			0.021578
GGT (G) / AAG (K)			0.021649
AGA (R) / ACC (T)			0.021698
ATC (I) / CCG (P)			0.021723
ACA (T) / CAC (H)			0.021920
GCC (A) / CCT (P)			0.022062
GGC (G) / GCT (A)			0.022214
AAG (K) / CTC (L)			0.022384
AAA (K) / ATC (I)			0.023020

codon (AA) substitution	ECM rate		
	synonymous	synonymous SER (S/Z)	nonsynonymous
GCG (A) / ACT (T)			0.023862
CGG (R) / TGT (C)			0.023909
GTG (V) / TTA (L)			0.024699
GGT (G) / GTG (V)			0.024883
AGT (Z) / CGG (R)			0.025006
CCA (P) / TTT (F)			0.025217
AAC (N) / ATT (I)			0.025405
ATA (I) / TAC (Y)			0.025454
AGA (R) / CAC (H)			0.025544
GAA (E) / CGC (R)			0.025678
CCA (P) / TAC (Y)			0.025873
ACA (T) / CCC (P)			0.026230
GTC (V) / TTT (F)			0.026338
CAT (H) / TTA (L)			0.026551
CCC (P) / CTT (L)			0.026571
GCG (A) / TAT (Y)			0.027035
ACC (T) / CCA (P)			0.027138
CGG (R) / TTC (F)			0.027355
GGT (G) / ATG (M)			0.027479
GAC (D) / GCA (A)			0.027912
GGC (G) / GCA (A)			0.027973
GAT (D) / TGG (W)			0.028249
GCG (A) / TGT (C)			0.028567
GCC (A) / GTA (V)			0.028569
AGG (R) / TCC (S)			0.028715
ATG (M) / CGT (R)			0.028736
GAA (E) / TGT (C)			0.028878
ACT (T) / CAG (Q)			0.029088
ATG (M) / CGC (R)			0.029786
GGA (G) / TAC (Y)			0.029826
TCC (S) / TTT (F)			0.030074
CGA (R) / TTT (F)			0.030121
GGT (G) / GAG (E)			0.030223
GAT (D) / AGA (R)			0.030474
GGC (G) / GAA (E)			0.030585
AGA (R) / TGC (C)			0.030883
AGC (Z) / ATT (I)			0.031140
GCA (A) / TGG (W)			0.031466
GAT (D) / CCG (P)			0.031560
GTC (V) / CCG (P)			0.031642
GTA (V) / CTC (L)			0.031730
GAG (E) / ATC (I)			0.031984
GGG (G) / TTC (F)			0.032039
CGC (R) / TCG (S)			0.032106
GTT (V) / AGA (R)			0.032116
CCA (P) / TAT (Y)			0.032140
GGG (G) / GTT (V)			0.032177
CAA (Q) / TGC (C)			0.032232

codon (AA) substitution	ECM rate synonymous	synonymous SER (S/Z)	nonsynonymous
GTG (V) / CAT (H)			0.032237
GAC (D) / ATC (I)			0.032447
CCC (P) / TGG (W)			0.032564
AGG (R) / CCC (P)			0.033244
CAA (Q) / CTG (L)			0.033372
TAC (Y) / TTA (L)			0.033529
GAG (E) / ACT (T)			0.033581
GAA (E) / ATG (M)			0.033994
TCG (S) / TTT (F)			0.034137
GAG (E) / TTC (F)			0.034428
GAG (E) / CCT (P)			0.034437
AAG (K) / CGA (R)			0.034519
ACC (T) / ATT (I)			0.034573
GCA (A) / TAC (Y)			0.034737
AAG (K) / TGC (C)			0.034794
AAC (N) / CTG (L)			0.034916
GAA (E) / CGT (R)			0.034978
GGT (G) / GAC (D)			0.035120
GCT (A) / TTG (L)			0.035630
GCT (A) / TGG (W)			0.035860
ACT (T) / TGG (W)			0.035939
GGA (G) / GCC (A)			0.036022
CCT (P) / CTC (L)			0.036254
ACG (T) / TTC (F)			0.036489
CCG (P) / TAC (Y)			0.037372
GGC (G) / GAT (D)			0.037461
GGT (G) / AGA (R)			0.037657
GGA (G) / ATG (M)			0.037724
ACC (T) / TGG (W)			0.037822
GAT (D) / TTG (L)			0.038557
GCC (A) / ACT (T)			0.038559
GAA (E) / ATT (I)			0.038612
GTT (V) / TGG (W)			0.038763
GCA (A) / CAT (H)			0.039265
GGT (G) / TGG (W)			0.039313
AAG (K) / CTT (L)			0.039362
CGG (R) / CTT (L)			0.039428
GAA (E) / CTT (L)			0.039560
TGC (C) / TTA (L)			0.040012
CCC (P) / CTA (L)			0.040087
ATA (I) / CAT (H)			0.040109
CAT (H) / TTG (L)			0.040275
AGG (R) / CTG (L)			0.040320
AAT (N) / TCG (S)			0.040706
CGG (R) / CTA (L)			0.040902
GCT (A) / CAG (Q)			0.040907
CCA (P) / CTG (L)			0.040917
GAC (D) / GTG (V)			0.041280

codon (AA) substitution	ECM rate		
	synonymous	synonymous SER (S/Z)	nonsynonymous
GCA (A) / AAC (N)			0.041435
AAG (K) / ATT (I)			0.041440
CGA (R) / CTG (L)			0.041969
GGT (G) / CGA (R)			0.042005
ATG (M) / CCA (P)			0.042006
GAT (D) / TTA (L)			0.042112
ACG (T) / TAT (Y)			0.042119
ATG (M) / TCC (S)			0.042282
GAG (E) / TCT (S)			0.042380
GAA (E) / GTC (V)			0.042565
GAT (D) / ACG (T)			0.042595
AAT (N) / TAC (Y)			0.042762
GGT (G) / CAA (Q)			0.042812
GAG (E) / TAT (Y)			0.042815
AAA (K) / TAC (Y)			0.042942
GAT (D) / GCG (A)			0.043198
GGG (G) / CGT (R)			0.043222
AAG (K) / TTC (F)			0.043609
GAA (E) / TAC (Y)			0.043721
GTC (V) / CTA (L)			0.043756
CGA (R) / TAT (Y)			0.043917
GTG (V) / CAC (H)			0.044112
ACC (T) / CCT (P)			0.044125
GCC (A) / TGG (W)			0.044178
CGT (R) / CCG (P)			0.044676
CAT (H) / CTG (L)			0.044742
GGT (G) / TGC (C)			0.045044
GCT (A) / AGG (R)			0.045374
TGC (C) / TTT (F)			0.045951
GCC (A) / GTT (V)			0.046583
AAC (N) / TAT (Y)			0.046586
GGA (G) / GCG (A)			0.046588
GAT (D) / CAC (H)			0.046739
TAT (Y) / TCC (S)			0.047115
GGC (G) / CTG (L)			0.047268
GTA (V) / TTC (F)			0.047403
GGT (G) / AGG (R)			0.047437
AGA (R) / TCT (S)			0.047890
GGT (G) / AAA (K)			0.048730
ATA (I) / CTG (L)			0.048882
ACA (T) / ATC (I)			0.048902
CAG (Q) / TCT (S)			0.049246
GAG (E) / GTT (V)			0.049842
GCG (A) / ACA (T)			0.049848
AAA (K) / TGG (W)			0.051005
GGC (G) / GTG (V)			0.051048
CGA (R) / TAC (Y)			0.051686
GTT (V) / AGG (R)			0.051709

codon (AA) substitution	ECM rate synonymous	synonymous SER (S/Z)	nonsynonymous
TCT (S) / TTC (F)			0.052602
GAG (E) / GTA (V)			0.053017
GAC (D) / AAA (K)			0.053168
ACA (T) / TAC (Y)			0.053363
GGG (G) / CAC (H)			0.053714
GGA (G) / GAG (E)			0.053748
ACT (T) / CCC (P)			0.053805
ACG (T) / TGT (C)			0.053874
AAC (N) / TGG (W)			0.053908
CAT (H) / CCG (P)			0.054021
GGC (G) / AGT (Z)			0.054025
GCG (A) / GTT (V)			0.054049
TGT (C) / TAC (Y)			0.054860
GAA (E) / GTG (V)			0.055025
CAA (Q) / TGG (W)			0.055163
GAC (D) / TTC (F)			0.055366
GGC (G) / ATC (I)			0.055425
ATC (I) / TTT (F)			0.056038
GTC (V) / AAG (K)			0.056145
GTT (V) / ACA (T)			0.056250
AAG (K) / ATA (I)			0.056267
TCA (S) / TTC (F)			0.056677
AGG (R) / TTC (F)			0.056771
CAT (H) / CTC (L)			0.057328
CAG (Q) / TGT (C)			0.057910
TAT (Y) / TCG (S)			0.058136
CAC (H) / CTT (L)			0.058614
GTA (V) / TGG (W)			0.058616
GAT (D) / ATG (M)			0.059065
CAC (H) / CTA (L)			0.059927
ATT (I) / TTC (F)			0.059956
ACA (T) / TGG (W)			0.060421
GTA (V) / TAC (Y)			0.060430
AGG (R) / CCT (P)			0.060432
AAC (N) / TTG (L)			0.060699
AGA (R) / TAC (Y)			0.060909
AAA (K) / CTT (L)			0.060922
ACA (T) / TGC (C)			0.061227
GGA (G) / TGC (C)			0.061497
CAC (H) / CCT (P)			0.061583
CCT (P) / TGG (W)			0.061833
AGG (R) / ATC (I)			0.062369
GCA (A) / AGC (Z)			0.063353
CAA (Q) / CCG (P)			0.063366
CGT (R) / CCA (P)			0.063413
AGG (R) / TCT (S)			0.063994
GGG (G) / GTC (V)			0.064227
GAT (D) / TTT (F)			0.064397

codon (AA) substitution	ECM rate synonymous	synonymous SER (S/Z)	nonsynonymous
CCG (P) / TCA (S)			0.064924
ATA (I) / TTC (F)			0.064968
AAC (N) / CGG (R)			0.065212
CCA (P) / CTT (L)			0.065669
GTT (V) / TTC (F)			0.065905
GAC (D) / TGG (W)			0.066384
ACC (T) / CAA (Q)			0.066519
GAT (D) / ATT (I)			0.066966
GGA (G) / GAC (D)			0.067431
AAT (N) / ACC (T)			0.067554
TGC (C) / TAT (Y)			0.068342
GCG (A) / AAC (N)			0.068501
CGT (R) / CTC (L)			0.068758
GTG (V) / TTT (F)			0.068927
GTC (V) / CAG (Q)			0.069320
ATA (I) / TGT (C)			0.069555
GGG (G) / AGA (R)			0.069567
TAC (Y) / TCT (S)			0.069588
GCA (A) / CCC (P)			0.069754
TGG (W) / TCT (S)			0.069758
GGT (G) / ATT (I)			0.069995
CCA (P) / TGG (W)			0.070308
AGG (R) / TGC (C)			0.070533
TAC (Y) / TCA (S)			0.070809
GAC (D) / TGC (C)			0.071612
CAA (Q) / CTC (L)			0.071794
GTT (V) / AAG (K)			0.072138
CCC (P) / TCA (S)			0.072352
GGT (G) / GTA (V)			0.072521
GTT (V) / CTC (L)			0.072866
AAG (K) / TAT (Y)			0.072867
GCT (A) / CCC (P)			0.073021
CTG (L) / TTT (F)			0.073550
ACC (T) / CTG (L)			0.073657
GGA (G) / TGG (W)			0.073682
CAT (H) / TCG (S)			0.073730
AAC (N) / CCA (P)			0.074161
GGG (G) / GTA (V)			0.074536
CAA (Q) / CCC (P)			0.074846
GCG (A) / TTC (F)			0.074851
AAT (N) / TGG (W)			0.074859
ACT (T) / ATC (I)			0.075878
ATT (I) / CGT (R)			0.076434
TGT (C) / TTC (F)			0.076912
ACG (T) / CAT (H)			0.076938
GCA (A) / ACC (T)			0.077341
CGT (R) / TGC (C)			0.077815
AAT (N) / TTG (L)			0.077878

codon (AA) substitution	ECM rate		
	synonymous	synonymous SER (S/Z)	nonsynonymous
ATT (I) / TGG (W)			0.078312
GAT (D) / GTA (V)			0.078381
CCT (P) / TCG (S)			0.078776
ATT (I) / CAA (Q)			0.078926
CAG (Q) / CTT (L)			0.079119
CCG (P) / TTC (F)			0.079161
CAA (Q) / TAC (Y)			0.079236
ACG (T) / CCT (P)			0.079712
ACA (T) / TTT (F)			0.079948
GCG (A) / CAT (H)			0.080124
GGA (G) / GTT (V)			0.080337
CAA (Q) / TCC (S)			0.080383
TGG (W) / TCC (S)			0.081312
ATG (M) / CCT (P)			0.081631
GCG (A) / GTA (V)			0.081861
CGG (R) / TAC (Y)			0.081931
GGA (G) / TAT (Y)			0.082323
ACA (T) / CAT (H)			0.082373
AGT (Z) / CAG (Q)			0.083261
AGG (R) / TTT (F)			0.083310
ACG (T) / CCA (P)			0.083475
GGT (G) / GCG (A)			0.083552
AGT (Z) / TCC (S)		0.083636	
CTC (L) / TTT (F)			0.083684
GCG (A) / CGC (R)			0.083744
GGG (G) / CAT (H)			0.084060
GCC (A) / TCA (S)			0.084218
GCA (A) / AAT (N)			0.084620
GAA (E) / TTT (F)			0.084945
GAA (E) / TCC (S)			0.085680
GGT (G) / GCC (A)			0.086923
GTA (V) / TGC (C)			0.086986
GCA (A) / CCG (P)			0.087384
AGA (R) / ACG (T)			0.087905
GGT (G) / GAA (E)			0.089148
ATT (I) / CTG (L)			0.089308
AAA (K) / ACC (T)			0.089476
GTG (V) / ACC (T)			0.089643
CAC (H) / CCA (P)			0.089835
ACG (T) / ATT (I)			0.090674
CGA (R) / CCG (P)			0.091073
CAT (H) / CCC (P)			0.091276
GTG (V) / CTT (L)			0.091844
GTC (V) / TGG (W)			0.092044
CAG (Q) / TAT (Y)			0.092070
GCA (A) / GTC (V)			0.092405
GAT (D) / ACA (T)			0.092988
ATG (M) / TCT (S)			0.093491

codon (AA) substitution	ECM rate		
	synonymous	synonymous SER (S/Z)	nonsynonymous
AGG (R) / ATT (I)			0.093542
AGC (Z) / TCT (S)		0.094762	
GAC (D) / ACG (T)			0.095207
AAC (N) / CCG (P)			0.095418
AAT (N) / CCA (P)			0.096104
GGG (G) / CGC (R)			0.097165
GGA (G) / CAT (H)			0.097554
AAA (K) / TGT (C)			0.099519
GAT (D) / TGT (C)			0.099985
GAC (D) / CAT (H)			0.100928
CCC (P) / TCT (S)			0.101204
ACG (T) / CAC (H)			0.103080
GCG (A) / TCT (S)			0.103323
GCC (A) / CTG (L)			0.103766
AGC (Z) / CCG (P)			0.104681
GAA (E) / CCC (P)			0.105112
AAG (K) / TGG (W)			0.105226
GGC (G) / TGG (W)			0.105305
GTC (V) / CTT (L)			0.105361
CAG (Q) / CTA (L)			0.105449
CAG (Q) / TGC (C)			0.105597
GGG (G) / TGT (C)			0.105884
ATC (I) / CAG (Q)			0.105885
GAA (E) / CAC (H)			0.106069
GCG (A) / TAC (Y)			0.106187
GAG (E) / GCT (A)			0.106257
CAG (Q) / TTC (F)			0.107218
CAG (Q) / CCT (P)			0.107434
GCT (A) / ACG (T)			0.108501
GAA (E) / ACC (T)			0.109664
GCA (A) / TTT (F)			0.109975
GCC (A) / TCT (S)			0.111968
CGA (R) / CTC (L)			0.112395
CCG (P) / TTG (L)			0.112999
CTC (L) / TGG (W)			0.113701
GGA (G) / TTT (F)			0.113991
CAC (H) / TCG (S)			0.114551
ATC (I) / TCC (S)			0.115975
AGA (R) / ATT (I)			0.116039
GTG (V) / TAC (Y)			0.116309
AAG (K) / ATC (I)			0.116405
GAT (D) / TCG (S)			0.117533
CCG (P) / CTC (L)			0.118229
GAC (D) / GTC (V)			0.119062
GCT (A) / ACC (T)			0.120121
GAC (D) / CAA (Q)			0.121389
ATA (I) / CGA (R)			0.121855
TGC (C) / TCA (S)			0.121937

codon (AA) substitution	ECM rate		
	synonymous	synonymous SER (S/Z)	nonsynonymous
AGG (R) / TGT (C)			0.122186
CGT (R) / CCC (P)			0.124232
CAA (Q) / TTT (F)			0.126320
GCA (A) / AGT (Z)			0.126382
GTG (V) / TGT (C)			0.126844
GGC (G) / TAC (Y)			0.127388
CGG (R) / TTG (L)			0.127696
ATC (I) / CAC (H)			0.127899
CAT (H) / TCA (S)			0.128111
GAG (E) / AGT (Z)			0.129098
GAT (D) / CTT (L)			0.129492
GAT (D) / CAG (Q)			0.130873
GGT (G) / TAT (Y)			0.130940
GCT (A) / GTC (V)			0.131929
AAG (K) / CTG (L)			0.133032
CGT (R) / CTA (L)			0.134394
GAC (D) / CTC (L)			0.135255
ATA (I) / CTC (L)			0.136148
GAG (E) / TAC (Y)			0.136432
CGA (R) / TTA (L)			0.136647
ATC (I) / TGG (W)			0.136849
GGG (G) / GAA (E)			0.136937
GAG (E) / TGG (W)			0.137634
TGC (C) / TCT (S)			0.138244
CCA (P) / TCC (S)			0.138742
AGT (Z) / TGG (W)			0.139046
AGA (R) / TTT (F)			0.140516
GAA (E) / GCC (A)			0.140546
AGG (R) / ACC (T)			0.140701
AAG (K) / TAC (Y)			0.140864
GTC (V) / TTG (L)			0.140867
AGG (R) / CAT (H)			0.140943
TGT (C) / TCG (S)			0.143447
AAA (K) / CAC (H)			0.143479
GGG (G) / CGA (R)			0.143481
AAC (N) / TTC (F)			0.143614
CGT (R) / TTT (F)			0.143832
AGA (R) / TGT (C)			0.144388
AGA (R) / TGG (W)			0.145245
CGA (R) / TGG (W)			0.146501
GAT (D) / CCA (P)			0.147873
AAA (K) / TTT (F)			0.148268
CGC (R) / CAA (Q)			0.148940
GAG (E) / AAT (N)			0.149578
CCC (P) / CTG (L)			0.149771
ACC (T) / TCA (S)			0.150107
GAC (D) / CGG (R)			0.150527
GCG (A) / TCA (S)			0.150589

codon (AA) substitution	ECM rate synonymous	synonymous SER (S/Z)	nonsynonymous
GTT (V) / CAA (Q)			0.150804
CCA (P) / TCG (S)			0.151589
TTA (L) / TTC (F)			0.151858
GCT (A) / GTG (V)			0.152215
GAT (D) / GTT (V)			0.153418
CGA (R) / CCC (P)			0.154017
GTC (V) / ATA (I)			0.154207
ACG (T) / ATA (I)			0.154441
AAG (K) / TCT (S)			0.156468
GAG (E) / GCA (A)			0.157605
TCC (S) / TTG (L)			0.158873
GAG (E) / CAT (H)			0.159217
GAG (E) / AAA (K)			0.159248
GAG (E) / TTG (L)			0.159955
GAT (D) / CGT (R)			0.162525
CAA (Q) / TGT (C)			0.163174
ATG (M) / CAC (H)			0.163406
GGT (G) / GTT (V)			0.164143
GGT (G) / TTT (F)			0.164659
GGA (G) / AGG (R)			0.165587
GGA (G) / CTA (L)			0.165784
CGA (R) / TGC (C)			0.166150
AGC (Z) / TTC (F)			0.166706
GAC (D) / AAT (N)			0.167041
AGC (Z) / TGG (W)			0.167138
AGG (R) / CAC (H)			0.169022
GCC (A) / ATG (M)			0.169264
GGT (G) / CTT (L)			0.169777
ATA (I) / TGG (W)			0.170041
CGC (R) / CAT (H)			0.170088
ATT (I) / TCT (S)			0.172403
GGC (G) / TTC (F)			0.172889
GTG (V) / CTA (L)			0.173122
ACG (T) / TGC (C)			0.173298
AAC (N) / ATC (I)			0.175491
GGG (G) / TTG (L)			0.175861
GGC (G) / AAG (K)			0.176224
GAG (E) / CTG (L)			0.176300
CGC (R) / TGG (W)			0.176476
GCA (A) / TAT (Y)			0.176829
ACG (T) / TAC (Y)			0.177757
TAC (Y) / TTG (L)			0.177833
CCT (P) / CTA (L)			0.181411
AGT (Z) / TGC (C)			0.181794
CGG (R) / TGC (C)			0.183143
GGG (G) / TGC (C)			0.183952
ATT (I) / CTC (L)			0.184010
CAG (Q) / CCA (P)			0.184327

codon (AA) substitution	ECM rate		
	synonymous	synonymous SER (S/Z)	nonsynonymous
GAC (D) / TAC (Y)			0.184704
GAA (E) / CCT (P)			0.184749
GGG (G) / CTG (L)			0.185038
AAC (N) / TCA (S)			0.187826
GCG (A) / AGG (R)			0.187832
AAC (N) / CTC (L)			0.187880
GAT (D) / AAG (K)			0.188097
AAT (N) / CAC (H)			0.188587
GAT (D) / GCA (A)			0.189090
GCG (A) / CAC (H)			0.190037
ATT (I) / CAT (H)			0.193328
ATG (M) / CCG (P)			0.193459
GAA (E) / AAC (N)			0.193544
GGA (G) / ATA (I)			0.194319
GGG (G) / AAT (N)			0.194964
CAT (H) / TGG (W)			0.195073
TGG (W) / TCA (S)			0.195833
CCT (P) / TCC (S)			0.196358
ACC (T) / TCT (S)			0.196983
GGA (G) / TGT (C)			0.197641
TCG (S) / TTC (F)			0.198277
CTA (L) / TTC (F)			0.198558
AAA (K) / ACG (T)			0.199589
ACT (T) / TCG (S)			0.199748
GCG (A) / AGC (Z)			0.200205
AGG (R) / TAT (Y)			0.200499
CGC (R) / TTC (F)			0.200806
GGA (G) / TTA (L)			0.201112
GGC (G) / GTC (V)			0.201477
GTT (V) / AAA (K)			0.201480
ATC (I) / CTA (L)			0.203017
AAT (N) / CAG (Q)			0.206851
CTT (L) / TTC (F)			0.207692
ACA (T) / TAT (Y)			0.208041
AAT (N) / ATG (M)			0.208924
TGG (W) / TGC (C)			0.210115
TAC (Y) / TCG (S)			0.213967
GTC (V) / AAC (N)			0.215492
CCC (P) / TGC (C)			0.215779
CCG (P) / TGG (W)			0.215788
GCT (A) / AAA (K)			0.216244
AAC (N) / ATG (M)			0.219170
AAG (K) / CCT (P)			0.220816
GCG (A) / TTG (L)			0.220908
AAA (K) / ATT (I)			0.221536
GAT (D) / TAT (Y)			0.223763
AAT (N) / ACG (T)			0.224397
AGT (Z) / TTT (F)			0.225554

codon (AA) substitution	ECM rate synonymous	ECM rate synonymous SER (S/Z)	ECM rate nonsynonymous
GAA (E) / GCG (A)			0.227527
AGC (Z) / CAA (Q)			0.232701
CGA (R) / TGT (C)			0.232728
GGG (G) / GAT (D)			0.235747
GTA (V) / TAT (Y)			0.236179
AGC (Z) / ATC (I)			0.238533
ATC (I) / CGC (R)			0.238839
GGG (G) / GCT (A)			0.238907
AAT (N) / ATT (I)			0.239388
CCT (P) / TGT (C)			0.239715
GGC (G) / GAG (E)			0.239725
GGC (G) / CTC (L)			0.240505
TGC (C) / TTG (L)			0.240632
GGT (G) / CGT (R)			0.241197
GAG (E) / GTC (V)			0.245019
ATG (M) / TCA (S)			0.246094
AAA (K) / CCT (P)			0.247195
GGA (G) / CGA (R)			0.247245
CAG (Q) / TGG (W)			0.247490
AGC (Z) / TGT (C)			0.248099
TCT (S) / TTA (L)			0.249707
GCT (A) / TCG (S)			0.250545
CAG (Q) / TTG (L)			0.250748
GAA (E) / ACT (T)			0.251423
AAG (K) / TCC (S)			0.251650
GTA (V) / CGA (R)			0.255147
ATC (I) / TAC (Y)			0.255975
AAG (K) / ACA (T)			0.256174
AAT (N) / ATA (I)			0.256283
GAA (E) / TAT (Y)			0.257122
AAT (N) / CTT (L)			0.259380
AAC (N) / ACA (T)			0.259909
CCA (P) / TTA (L)			0.261069
GAG (E) / ATG (M)			0.262465
AAT (N) / TTT (F)			0.263567
TGG (W) / TGT (C)			0.264556
CAC (H) / TAT (Y)			0.265766
CTG (L) / TGC (C)			0.265948
CTG (L) / TCG (S)			0.266943
GCT (A) / AAG (K)			0.268662
AGG (R) / TAC (Y)			0.269510
AGT (Z) / ATA (I)			0.269862
ATC (I) / CCC (P)			0.270274
TGG (W) / TTA (L)			0.271072
AAT (N) / TTA (L)			0.271628
GCT (A) / CAA (Q)			0.272950
AGA (R) / AAC (N)			0.273855
AGG (R) / CCG (P)			0.273876

codon (AA) substitution	ECM rate		
	synonymous	synonymous SER (S/Z)	nonsynonymous
TCT (S) / TTG (L)			0.274990
AAA (K) / ATG (M)			0.275355
AGT (Z) / ACC (T)			0.275865
GGG (G) / GTG (V)			0.276276
GAA (E) / GTT (V)			0.276499
GTG (V) / TGG (W)			0.277072
CGT (R) / CAC (H)			0.277265
CTT (L) / TCT (S)			0.277929
TCA (S) / TTT (F)			0.278090
GGA (G) / AAC (N)			0.280400
GAG (E) / TCC (S)			0.283432
AAA (K) / CAG (Q)			0.285384
CCC (P) / TTC (F)			0.285635
CAT (H) / CCA (P)			0.286252
GCA (A) / CGA (R)			0.286547
GAA (E) / TTA (L)			0.287373
AAG (K) / ACT (T)			0.291009
ATT (I) / CCT (P)			0.292855
GGG (G) / AAC (N)			0.293884
GAG (E) / CCC (P)			0.294294
ATG (M) / TAT (Y)			0.294368
ACA (T) / ATT (I)			0.296014
AAC (N) / ACT (T)			0.296066
AAA (K) / CGC (R)			0.296358
GCA (A) / TGC (C)			0.297371
ATG (M) / TGT (C)			0.298091
GGA (G) / AGC (Z)			0.298738
CGG (R) / CAA (Q)			0.299978
ATG (M) / TAC (Y)			0.300354
AGC (Z) / CTC (L)			0.300513
AAG (K) / CCC (P)			0.303613
AGC (Z) / ATG (M)			0.304581
AGA (R) / ATG (M)			0.305714
AGT (Z) / ATG (M)			0.306375
GAA (E) / AGC (Z)			0.308372
GAA (E) / AAG (K)			0.308851
CTG (L) / TAC (Y)			0.308859
GGC (G) / TGC (C)			0.309374
ATC (I) / TGC (C)			0.309643
AGG (R) / ACT (T)			0.309656
CCC (P) / TAC (Y)			0.310416
ACG (T) / ATC (I)			0.311759
GTG (V) / AAG (K)			0.311885
GAA (E) / CTA (L)			0.313495
GTA (V) / ATC (I)			0.313779
GTG (V) / AGG (R)			0.314379
CGG (R) / CAT (H)			0.316372
AGG (R) / ATA (I)			0.317466

codon (AA) substitution	ECM rate synonymous	synonymous SER (S/Z)	nonsynonymous
GCA (A) / ACG (T)			0.318574
GTT (V) / AAT (N)			0.318595
ATG (M) / TGG (W)			0.321642
AGT (Z) / CTT (L)			0.322807
ATG (M) / CAT (H)			0.323588
GCT (A) / ACA (T)			0.324162
GCT (A) / CGT (R)			0.324227
CCT (P) / TTT (F)			0.324368
AAG (K) / CAT (H)			0.324525
ATG (M) / TGC (C)			0.324613
GGA (G) / GTA (V)			0.324696
AAC (N) / TGC (C)			0.325422
GGT (G) / AGC (Z)			0.326035
CAC (H) / CTG (L)			0.330036
GAA (E) / TCT (S)			0.330481
CAG (Q) / TAC (Y)			0.332396
AAG (K) / TTG (L)			0.333780
AGC (Z) / AAA (K)			0.334224
GCA (A) / CCT (P)			0.335924
GGC (G) / CAG (Q)			0.336277
GTT (V) / CGT (R)			0.336828
TAT (Y) / TTG (L)			0.337279
CCT (P) / TAT (Y)			0.337879
GTT (V) / CAT (H)			0.338207
CAT (H) / TAC (Y)			0.340541
CGT (R) / CAG (Q)			0.340811
AAA (K) / TCT (S)			0.340991
TAT (Y) / TCA (S)			0.341791
CTC (L) / TCC (S)			0.342813
GAG (E) / CAA (Q)			0.343314
TGT (C) / TCC (S)			0.343463
ACT (T) / CGT (R)			0.344601
ACT (T) / CCA (P)			0.345545
AGC (Z) / TAC (Y)			0.346890
GCT (A) / CCA (P)			0.346978
GAT (D) / TCA (S)			0.348448
ACA (T) / TCG (S)			0.349846
GTC (V) / ATG (M)			0.350473
ACT (T) / ATA (I)			0.351882
ATT (I) / TAT (Y)			0.352731
CGA (R) / CCT (P)			0.352807
AGC (Z) / CGA (R)			0.354545
GGC (G) / TCG (S)			0.356038
AGT (Z) / ATT (I)			0.359183
AAT (N) / TGT (C)			0.360038
GAG (E) / ACC (T)			0.360196
ACA (T) / CGA (R)			0.360575
ACG (T) / TCT (S)			0.361164

codon (AA) substitution	ECM rate synonymous	synonymous SER (S/Z)	nonsynonymous
GAA (E) / ATA (I)			0.361403
GAG (E) / GTG (V)			0.362328
ACG (T) / TGG (W)			0.362681
CAC (H) / CCG (P)			0.364129
ACA (T) / CCT (P)			0.368538
CAC (H) / TGG (W)			0.369273
GCT (A) / TAT (Y)			0.370309
CCG (P) / TCC (S)			0.371063
GTG (V) / TTC (F)			0.371072
ACC (T) / TTC (F)			0.371541
GAT (D) / AAC (N)			0.371685
GAG (E) / AAC (N)			0.372719
GCC (A) / AAG (K)			0.373948
ACT (T) / CAA (Q)			0.374419
GGA (G) / GAT (D)			0.375097
AAC (N) / CAA (Q)			0.377447
GGT (G) / ACT (T)			0.380374
GCC (A) / TAC (Y)			0.380788
GTC (V) / AGC (Z)			0.381287
GGG (G) / TGG (W)			0.381671
GCA (A) / ACT (T)			0.381796
AGC (Z) / CGT (R)			0.387941
GGT (G) / CAT (H)			0.388156
AGT (Z) / AAG (K)			0.389004
ATA (I) / CAA (Q)			0.390087
ATC (I) / CTT (L)			0.390190
ACC (T) / TAC (Y)			0.393545
GGC (G) / CCG (P)			0.395771
TGT (C) / TTG (L)			0.395942
GAT (D) / GCT (A)			0.396521
GTC (V) / TCC (S)			0.396897
ATA (I) / TCA (S)			0.403435
GGG (G) / GAC (D)			0.403521
AGA (R) / AGC (Z)			0.403913
ATG (M) / CGG (R)			0.408680
AGA (R) / CTA (L)			0.409432
ACA (T) / TCC (S)			0.409721
TGG (W) / TCG (S)			0.410046
GGG (G) / ATG (M)			0.410199
AGA (R) / CAT (H)			0.411115
AAA (K) / AAC (N)			0.413957
GCA (A) / GTG (V)			0.415718
ACA (T) / TGT (C)			0.415775
GCG (A) / CCC (P)			0.417565
GGT (G) / CCT (P)			0.418802
ACT (T) / ATG (M)			0.419831
GCT (A) / CAT (H)			0.420580
GGT (G) / TGT (C)			0.420899

codon (AA) substitution	ECM rate synonymous	synonymous SER (S/Z)	nonsynonymous
CAG (Q) / CTC (L)			0.422671
CAG (Q) / TCC (S)			0.423382
AGA (R) / TTA (L)			0.423774
AGA (R) / TAT (Y)			0.424321
CGA (R) / CTT (L)			0.424607
GAC (D) / AAG (K)			0.426684
GTT (V) / ATG (M)			0.430124
ATT (I) / TGT (C)			0.431456
CCC (P) / TCG (S)			0.432520
AGA (R) / ACT (T)			0.432918
AAA (K) / CTA (L)			0.433620
ACC (T) / ATG (M)			0.433759
CAA (Q) / CCT (P)			0.435109
ACG (T) / CCC (P)			0.435243
CAT (H) / CTA (L)			0.438648
GAA (E) / CGA (R)			0.440217
AGC (Z) / ACA (T)			0.441084
ATG (M) / CAA (Q)			0.443617
GCG (A) / TGG (W)			0.446015
GCT (A) / ATG (M)			0.451134
GTG (V) / TCG (S)			0.451899
GAA (E) / CAT (H)			0.452442
AGG (R) / AAT (N)			0.454347
GTC (V) / ATT (I)			0.458622
AAC (N) / CAT (H)			0.460970
AAG (K) / CAA (Q)			0.461383
GCA (A) / AGA (R)			0.461729
GGA (G) / ACA (T)			0.466770
AAA (K) / TAT (Y)			0.467136
GAA (E) / GTA (V)			0.469281
GTG (V) / CAG (Q)			0.475496
GTG (V) / CTC (L)			0.477439
ATC (I) / TTG (L)			0.478019
ATA (I) / CCA (P)			0.480521
TAC (Y) / TTT (F)			0.481042
CAC (H) / TGC (C)			0.483076
GCT (A) / TTT (F)			0.483563
CTT (L) / TAT (Y)			0.485469
CAA (Q) / TAT (Y)			0.486281
ACT (T) / TCC (S)			0.487317
CCA (P) / TCT (S)			0.487542
AAC (N) / TAC (Y)			0.487814
GTC (V) / CAC (H)			0.491034
TGC (C) / TCG (S)			0.493179
AGA (R) / CCA (P)			0.493508
CCG (P) / CTG (L)			0.495176
CTA (L) / TCA (S)			0.496046
GGG (G) / GCC (A)			0.496552

codon (AA) substitution	ECM rate synonymous	synonymous SER (S/Z)	nonsynonymous
AGG (R) / TGG (W)			0.496706
ACT (T) / TTT (F)			0.497293
AAG (K) / ATG (M)			0.497593
GCC (A) / ACG (T)			0.498504
CGT (R) / CCT (P)			0.500433
GTT (V) / AGT (Z)			0.501456
GAA (E) / GCT (A)			0.502355
TAT (Y) / TTC (F)			0.503397
CTG (L) / TGG (W)			0.504866
GCA (A) / TCG (S)			0.505677
AGT (Z) / TAT (Y)			0.506470
AGG (R) / TTG (L)			0.506841
CAA (Q) / TCT (S)			0.508308
GTA (V) / CTT (L)			0.509595
AGG (R) / ACA (T)			0.511968
GCC (A) / TTC (F)			0.512203
AGG (R) / TCG (S)			0.514392
ACG (T) / TCA (S)			0.516594
CGC (R) / TGC (C)			0.516927
GCA (A) / ATG (M)			0.517896
GGC (G) / ACC (T)			0.518903
TAC (Y) / TCC (S)			0.524116
AAG (K) / AAT (N)			0.524647
CGA (R) / CAC (H)			0.526904
ATG (M) / TCG (S)			0.527005
ACC (T) / CCG (P)			0.527094
GAG (E) / CGC (R)			0.528692
CAA (Q) / CTT (L)			0.529045
CAG (Q) / CCC (P)			0.529594
GTT (V) / TTG (L)			0.529619
GTG (V) / TGC (C)			0.530278
AGT (Z) / CGA (R)			0.530881
AAC (N) / CCC (P)			0.532092
CGT (R) / TAT (Y)			0.534375
GCT (A) / ATT (I)			0.536332
GCC (A) / ATC (I)			0.536362
GAA (E) / CAG (Q)			0.536567
CTT (L) / TGT (C)			0.543240
CCT (P) / TCA (S)			0.544474
CGC (R) / CTC (L)			0.544828
GGA (G) / CAA (Q)			0.551493
GTT (V) / CTA (L)			0.555104
GGA (G) / CCA (P)			0.556204
CGA (R) / TCA (S)			0.557015
GCA (A) / TCC (S)			0.558500
GTA (V) / AGA (R)			0.560642
AAG (K) / CGT (R)			0.560831
AAG (K) / CCG (P)			0.561523

codon (AA) substitution	ECM rate		
	synonymous	synonymous SER (S/Z)	nonsynonymous
TGC (C) / TTC (F)			0.561620
AGT (Z) / ACG (T)			0.563566
CGC (R) / CCC (P)			0.563799
CGC (R) / CTG (L)			0.563956
GTT (V) / TCT (S)			0.563995
GTA (V) / TTT (F)			0.565566
GCC (A) / AAC (N)			0.565968
GAG (E) / CAC (H)			0.566034
GGC (G) / CAC (H)			0.566759
GTA (V) / ATG (M)			0.569076
AAG (K) / CAC (H)			0.571469
GTT (V) / TAT (Y)			0.571505
ATA (I) / TAT (Y)			0.573013
GTT (V) / ATC (I)			0.575921
CAA (Q) / TTA (L)			0.576476
GTC (V) / TAC (Y)			0.582801
GGT (G) / GCA (A)			0.585071
GCG (A) / TGC (C)			0.586111
AGT (Z) / AAC (N)			0.586685
ACG (T) / TTG (L)			0.586818
GGG (G) / CGG (R)			0.590833
ACT (T) / TAT (Y)			0.599932
AGG (R) / AAC (N)			0.600415
GTG (V) / CCG (P)			0.602428
CGC (R) / TAC (Y)			0.604541
GAT (D) / AAA (K)			0.604859
GTA (V) / CAA (Q)			0.606945
TCA (S) / TTG (L)			0.611887
AAA (K) / TTA (L)			0.612660
CGA (R) / CAG (Q)			0.617320
ACC (T) / CAG (Q)			0.617464
CAC (H) / CTC (L)			0.617694
CTC (L) / TAC (Y)			0.622089
AGC (Z) / ACT (T)			0.622868
AAG (K) / ACC (T)			0.623366
CGA (R) / CCA (P)			0.626603
TGC (C) / TAC (Y)			0.628438
GGT (G) / GAT (D)			0.629243
TGG (W) / TTG (L)			0.632947
CAT (H) / TGT (C)			0.634065
GGG (G) / CAG (Q)			0.634203
GCC (A) / CAG (Q)			0.634927
GGC (G) / CCC (P)			0.636916
CAT (H) / TTT (F)			0.638696
GGA (G) / GAA (E)			0.639125
CGG (R) / CTC (L)			0.640495
GCT (A) / AAT (N)			0.643160
AGC (Z) / AAT (N)			0.643789

codon (AA) substitution	ECM rate		
	synonymous	synonymous SER (S/Z)	nonsynonymous
GAC (D) / CCG (P)			0.644235
TGT (C) / TTA (L)			0.645571
GTC (V) / CGC (R)			0.648523
CCC (P) / CTC (L)			0.648769
TCC (S) / TTC (F)			0.656004
GGA (G) / GCT (A)			0.658541
CGT (R) / TCT (S)			0.660622
GAT (D) / CCT (P)			0.661776
GCT (A) / GTA (V)			0.662578
CGT (R) / CTT (L)			0.663877
CAT (H) / CTT (L)			0.664866
AAA (K) / ATA (I)			0.667397
GAA (E) / AGA (R)			0.668173
GAC (D) / TCG (S)			0.670108
GGG (G) / GCA (A)			0.672077
CTC (L) / TGC (C)			0.674176
TTG (L) / TTC (F)			0.675537
GGC (G) / CGC (R)			0.676049
ATG (M) / TTT (F)			0.679370
AGT (Z) / TCG (S)		0.681575	
AAT (N) / CGT (R)			0.682254
GCC (A) / GTG (V)			0.682579
GGG (G) / AGT (Z)			0.684968
CGC (R) / TCC (S)			0.685812
TAT (Y) / TCT (S)			0.688169
TCG (S) / TTG (L)			0.694091
GCC (A) / CAC (H)			0.696016
GTG (V) / CGG (R)			0.701399
GAC (D) / GCG (A)			0.702594
GGG (G) / CCG (P)			0.703092
GGC (G) / CGG (R)			0.703728
CAG (Q) / CTG (L)			0.703795
GAT (D) / CAA (Q)			0.706782
GTA (V) / AAA (K)			0.708204
GTT (V) / CCT (P)			0.708395
GGA (G) / AAA (K)			0.713961
CAG (Q) / CCG (P)			0.715716
GGG (G) / ACG (T)			0.716441
GCA (A) / GTT (V)			0.718719
AAC (N) / TCG (S)			0.725836
CGT (R) / TGT (C)			0.726908
GCG (A) / ACC (T)			0.728891
CAC (H) / CCC (P)			0.730306
GTA (V) / CCA (P)			0.732111
ACG (T) / CTG (L)			0.733202
TGT (C) / TTT (F)			0.733587
GCA (A) / CTA (L)			0.741135
GGG (G) / AAG (K)			0.744000

codon (AA) substitution	ECM rate synonymous	synonymous SER (S/Z)	nonsynonymous
GAT (D) / CAT (H)			0.746792
AAT (N) / TAT (Y)			0.747870
GTT (V) / TTA (L)			0.748196
CAA (Q) / CAC (H)			0.751904
TGT (C) / TCA (S)			0.751980
GTA (V) / TGT (C)			0.754233
GCG (A) / AAG (K)			0.759132
GTC (V) / CCC (P)			0.760090
CAC (H) / TTC (F)			0.761771
GTA (V) / TTG (L)			0.762425
AGG (R) / ACG (T)			0.765495
AAG (K) / TCG (S)			0.768607
GCT (A) / TCC (S)			0.771771
AGT (Z) / CGT (R)			0.772349
TCT (S) / TTT (F)			0.773935
CAT (H) / CCT (P)			0.775254
AGA (R) / ATA (I)			0.780008
TGG (W) / TTT (F)			0.786871
AAT (N) / CCT (P)			0.787340
CGG (R) / CTG (L)			0.794366
CGC (R) / CCG (P)			0.798346
TAT (Y) / TTA (L)			0.798582
ATG (M) / TTC (F)			0.800602
GAC (D) / GCC (A)			0.802516
ACC (T) / TGC (C)			0.806071
GCA (A) / TGT (C)			0.806554
GTA (V) / TCA (S)			0.813720
AAC (N) / ACG (T)			0.822133
GAG (E) / AGC (Z)			0.822643
TGT (C) / TAT (Y)			0.822999
ACT (T) / CTT (L)			0.831215
GAA (E) / AGT (Z)			0.832592
ATG (M) / CAG (Q)			0.834976
GCG (A) / ATG (M)			0.837302
ACA (T) / ATG (M)			0.853779
AAC (N) / CGC (R)			0.863191
GTT (V) / TTT (F)			0.863970
ACC (T) / CCC (P)			0.870967
ACA (T) / TTA (L)			0.871568
AAT (N) / TCA (S)			0.872084
AAA (K) / ACT (T)			0.878163
GCT (A) / CTT (L)			0.879741
GGC (G) / TCC (S)			0.880032
GAA (E) / CCA (P)			0.890822
GAA (E) / GAC (D)			0.892903
CGG (R) / CCC (P)			0.897101
ACC (T) / CTC (L)			0.897490
ACT (T) / CAT (H)			0.898794

codon (AA) substitution	ECM rate synonymous	synonymous SER (S/Z)	nonsynonymous
AAA (K) / CAT (H)			0.899262
GAA (E) / GCA (A)			0.905488
AGG (R) / ATG (M)			0.905810
GGA (G) / AAT (N)			0.909861
GTG (V) / ATA (I)			0.911193
GAC (D) / CGC (R)			0.915505
CGA (R) / CTA (L)			0.918198
GCA (A) / AAA (K)			0.923644
GAC (D) / CAG (Q)			0.928319
AGC (Z) / AAG (K)			0.928876
GAT (D) / ACT (T)			0.928969
AGA (R) / AAT (N)			0.936840
GGA (G) / AGA (R)			0.938439
GAG (E) / GCC (A)			0.938586
ACA (T) / CTA (L)			0.944560
GTC (V) / TTC (F)			0.946136
GTG (V) / ATT (I)			0.953353
ACG (T) / CCG (P)			0.962295
GGG (G) / GAG (E)			0.968146
GCG (A) / GTC (V)			0.968351
ACC (T) / CAC (H)			0.969387
GCC (A) / CCG (P)			0.971148
GAA (E) / AAT (N)			0.975582
GAC (D) / CAC (H)			0.975727
GAC (D) / CCC (P)			0.976175
GGT (G) / TCT (S)			0.976185
GCA (A) / ATA (I)			0.983926
AAC (N) / CAG (Q)			0.985145
GAG (E) / CCG (P)			0.990330
ATC (I) / TTC (F)			1.006045
GAC (D) / ACC (T)			1.013424
ACA (T) / CCA (P)			1.018005
GAC (D) / TCC (S)			1.023142
GAG (E) / TCG (S)			1.029128
AGT (Z) / CCT (P)			1.035015
CTA (L) / TTT (F)			1.036474
AAG (K) / AAC (N)			1.038682
GCA (A) / TTA (L)			1.041312
AAA (K) / (P) CCA			1.042457
CTG (L) / TTA (L)	1.045943		
AGC (Z) / TCA (S)		1.049877	
ACT (T) / CCT (P)			1.050363
CAA (Q) / CCA (P)			1.052040
ATA (I) / TTG (L)			1.065537
ACC (T) / ATC (I)			1.066285
CCA (P) / CTA (L)			1.073790
ATA (I) / TTT (F)			1.075187
GTC (V) / CTG (L)			1.085170

codon (AA) substitution	ECM rate		
	synonymous	synonymous SER (S/Z)	nonsynonymous
ACT (T) / TGT (C)			1.089585
GAG (E) / GAT (D)			1.091224
AGA (R) / TCA (S)			1.094736
CAG (Q) / CAT (H)			1.106179
GTG (V) / TTG (L)			1.108802
GGC (G) / AAC (N)			1.110726
AGA (R) / CGC (R)	1.113671		
AAC (N) / TCC (S)			1.118208
ATT (I) / TTT (F)			1.119411
GAT (D) / TCT (S)			1.120532
CGG (R) / TGG (W)			1.135910
TGG (W) / TAT (Y)			1.140051
AAG (K) / ACG (T)			1.144639
CAC (H) / TCC (S)			1.144692
AGG (R) / CAG (Q)			1.148387
GGG (G) / AGC (Z)			1.149846
ATG (M) / ATT (I)			1.155098
CGT (R) / CAA (Q)			1.164262
CGG (R) / CCG (P)			1.164525
GAA (E) / ACA (T)			1.164866
AGT (Z) / CAA (Q)			1.177718
GGA (G) / AGT (Z)			1.179053
TGG (W) / TTC (F)			1.183337
TCA (S) / TTA (L)			1.184813
GCC (A) / CTC (L)			1.191514
GAG (E) / AGG (R)			1.195790
AGA (R) / AAG (K)			1.197614
TTG (L) / TTT (F)			1.204356
CAA (Q) / CTA (L)			1.205738
ATG (M) / CTT (L)			1.220020
GGC (G) / GAC (D)			1.228004
GCA (A) / CCA (P)			1.236457
AGA (R) / CAA (Q)			1.242140
AAT (N) / ACA (T)			1.243753
GAG (E) / GCG (A)			1.251589
CAT (H) / TCT (S)			1.253945
ATA (I) / CTT (L)			1.260239
GCG (A) / TCC (S)			1.262618
GAA (E) / TCA (S)			1.265487
ACT (T) / ATT (I)			1.266654
GCT (A) / CCT (P)			1.272426
GTC (V) / TGC (C)			1.273908
GCA (A) / CAA (Q)			1.274118
AGC (Z) / CAC (H)			1.282522
AGG (R) / AGC (Z)			1.286990
ATT (I) / TTG (L)			1.292935
AGT (Z) / CAT (H)			1.293228
AGT (Z) / AAA (K)			1.293860

codon (AA) substitution	ECM rate		
	synonymous	synonymous SER (S/Z)	nonsynonymous
AGA (R) / ACA (T)			1.316696
AGC (Z) / CGC (R)			1.320875
GGT (G) / AAT (N)			1.322234
TGC (C) / TCC (S)			1.323765
ATT (I) / CTA (L)			1.328581
AGC (Z) / CCC (P)			1.337629
CTG (L) / TTC (F)			1.341144
AAC (N) / ACC (T)			1.346385
CGA (R) / CAT (H)			1.353860
AGC (Z) / CGG (R)			1.360141
AGC (Z) / TGC (C)			1.372357
CTC (L) / TTA (L)	1.373823		
ACG (T) / ATG (M)			1.376727
GCC (A) / CCC (P)			1.394892
GGC (G) / GCG (A)			1.398079
GTG (V) / ATG (M)			1.399673
AGC (Z) / CAG (Q)			1.418383
ATG (M) / TTA (L)			1.418466
TGG (W) / TAC (Y)			1.421996
ATG (M) / ATC (I)			1.428293
ATG (M) / CTC (L)			1.434579
GAG (E) / ACG (T)			1.447392
AGG (R) / AGT (Z)			1.477696
ACC (T) / CGC (R)			1.481721
AAA (K) / TCA (S)			1.496628
GCC (A) / TGC (C)			1.514097
CAG (Q) / TCG (S)			1.519211
AAA (K) / ACA (T)			1.523251
GCG (A) / CTG (L)			1.524024
GGG (G) / GCG (A)			1.526141
GCG (A) / CAG (Q)			1.544626
GGG (G) / AGG (R)			1.558784
ACG (T) / TCC (S)			1.562591
GAG (E) / AAG (K)			1.563846
GGT (G) / GCT (A)			1.572808
GTT (V) / TGT (C)			1.598824
GCC (A) / CGC (R)			1.610248
ATG (M) / CTA (L)			1.635281
AGA (R) / AGT (Z)			1.644621
ACA (T) / CAA (Q)			1.660885
GGA (G) / GCA (A)			1.693998
GAT (D) / AGT (Z)			1.702817
GGA (G) / TCA (S)			1.720919
AAT (N) / CAA (Q)			1.759732
ACG (T) / CAG (Q)			1.766961
AAA (K) / CGT (R)			1.769879
AAT (N) / TCT (S)			1.773102
GGC (G) / GCC (A)			1.797671

codon (AA) substitution	ECM rate		
	synonymous	synonymous SER (S/Z)	nonsynonymous
TGT (C) / TCT (S)			1.811753
GCT (A) / TCA (S)			1.825878
AGG (R) / AAA (K)			1.868194
GTT (V) / CTT (L)			1.897150
GCT (A) / TGT (C)			1.899865
CGG (R) / TCG (S)			1.903571
GCA (A) / TCT (S)			1.931350
GCG (A) / CCG (P)			1.950083
GCC (A) / TCG (S)			1.978448
GAC (D) / AGC (Z)			2.005334
AGT (Z) / TGT (C)			2.007768
CTT (L) / TTT (F)			2.016257
ACA (T) / TCT (S)			2.018483
CGC (R) / CAG (Q)			2.029914
GTA (V) / CTA (L)			2.047159
AAT (N) / ACT (T)			2.057449
CAA (Q) / TCA (S)			2.066955
AAA (K) / AAT (N)			2.075154
CCG (P) / TCG (S)			2.075226
ATT (I) / TTA (L)			2.130663
GCC (A) / ACC (T)			2.159328
GGG (G) / TCG (S)			2.163175
GAG (E) / CGG (R)			2.171984
AAC (N) / CAC (H)			2.203539
CGG (R) / CAC (H)			2.208430
GCG (A) / GTG (V)			2.211488
GCC (A) / AGC (Z)			2.214735
ATG (M) / ATA (I)			2.230691
ATG (M) / CTG (L)			2.236557
GAA (E) / AAA (K)			2.258321
ACA (T) / ATA (I)			2.260424
GTG (V) / CTG (L)			2.262490
AAG (K) / CAG (Q)			2.285052
ACT (T) / TCA (S)			2.297807
GTA (V) / TTA (L)			2.299543
CTC (L) / TTC (F)			2.306110
GCT (A) / AGT (Z)			2.323091
GCT (A) / ACT (T)			2.359362
GGT (G) / AGT (Z)			2.382451
GGC (G) / AGC (Z)			2.392606
TTA (L) / TTT (F)			2.395822
ACC (T) / TCG (S)			2.395833
AGT (Z) / ACA (T)			2.397415
AGA (R) / CGG (R)	2.418859		
CGT (R) / CAT (H)			2.460976
ATC (I) / CTG (L)			2.469249
CAA (Q) / CAT (H)			2.473439
CGC (R) / CAC (H)			2.478358

codon (AA) substitution	ECM rate		
	synonymous	synonymous SER (S/Z)	nonsynonymous
CCC (P) / TCC (S)			2.487136
CAG (Q) / CAC (H)			2.503268
GTT (V) / ATA (I)			2.577578
GTA (V) / ATT (I)			2.581654
GCT (A) / GTT (V)			2.600428
GCC (A) / GTC (V)			2.620833
AAT (N) / CAT (H)			2.691226
GTC (V) / CTC (L)			2.720153
AAG (K) / CGC (R)			2.721043
GAC (D) / AAC (N)			2.729647
GCA (A) / ACA (T)			2.735831
GAA (E) / GAT (D)			2.738552
CCT (P) / TCT (S)			2.846677
GTC (V) / ACC (T)			2.862216
GCG (A) / ACG (T)			2.866325
AGC (Z) / ACG (T)			2.871848
GTT (V) / ACT (T)			2.898791
ACG (T) / CGG (R)			2.985421
GAG (E) / CAG (Q)			3.036767
ATG (M) / TTG (L)			3.062807
AAA (K) / CGA (R)			3.065510
GAA (E) / CAA (Q)			3.081614
CAT (H) / TAT (Y)			3.088481
GTG (V) / ACG (T)			3.092500
CCA (P) / TCA (S)			3.121656
CAC (H) / TAC (Y)			3.193996
GAG (E) / GAC (D)			3.195698
AAA (K) / CAA (Q)			3.215817
ATT (I) / CTT (L)			3.330793
GCG (A) / CGG (R)			3.409178
GCA (A) / GTA (V)			3.415722
GCC (A) / TCC (S)			3.452570
GTG (V) / ATC (I)			3.487342
GAT (D) / AAT (N)			3.529593
ATC (I) / CTC (L)			3.765697
ACC (T) / TCC (S)			3.829580
AAG (K) / CGG (R)			3.832200
GTA (V) / ACA (T)			3.879355
GCT (A) / TCT (S)			3.994417
AGG (R) / CGC (R)	4.087042		
AGC (Z) / AAC (N)			4.127466
TAT (Y) / TTT (F)			4.317981
GCA (A) / TCA (S)			4.380679
ATA (I) / CTA (L)			4.400610
TAC (Y) / TTC (F)			4.483501
GCG (A) / TCG (S)			4.658653
ACT (T) / TCT (S)			4.693944
AGC (Z) / ACC (T)			4.699375

codon (AA) substitution	ECM rate synonymous	synonymous SER (S/Z)	nonsynonymous
CGG (R) / CAG (Q)			4.718199
CGA (R) / CAA (Q)			4.725840
CTG (L) / CTT (L)	4.802017		
ACG (T) / TCG (S)			4.919174
AGT (Z) / ACT (T)			4.943439
AGT (Z) / AAT (N)			4.971507
GCG (A) / GCT (A)	5.140068		
ATA (I) / TTA (L)			5.159075
GGA (G) / GGC (G)	5.214689		
GTA (V) / GTC (V)	5.637509		
CTC (L) / TTG (L)	5.651603		
ACA (T) / TCA (S)			5.709933
ATA (I) / ATC (I)	5.784672		
AGA (R) / AAA (K)			5.815294
AGT (Z) / TCA (S)		5.964323	
GTT (V) / ATT (I)			6.546163
GCA (A) / GCC (A)	6.746560		
AGG (R) / CGT (R)	6.798629		
AGG (R) / AAG (K)			7.316623
GTC (V) / ATC (I)			7.487816
CTG (L) / CTA (L)	8.319767		
ACA (T) / ACC (T)	8.373639		
CTA (L) / CTC (L)	8.808275		
GTG (V) / GTT (V)	8.832621		
GCC (A) / GCT (A)	9.612709		
AGC (Z) / TCG (S)		9.818281	
CCG (P) / CCT (P)	10.291826		
AGC (Z) / TCC (S)		10.892976	
GTC (V) / GTT (V)	10.956917		
CTT (L) / TTG (L)	11.161511		
GTA (V) / ATA (I)			11.271062
TCG (S) / TCT (S)	11.510965		
GCG (A) / GCA (A)	11.561124		
CTT (L) / TTA (L)	12.035723		
ACG (T) / ACT (T)	12.116657		
AGT (Z) / TCT (S)		12.169045	
CTG (L) / TTG (L)	12.455337		
CGA (R) / CGC (R)	12.677657		
CGG (R) / CGT (R)	12.868484		
AAG (K) / AAA (K)	12.931524		
GAG (E) / GAA (E)	12.984714		
ATC (I) / ATT (I)	13.609310		
GGC (G) / GGT (G)	13.935950		
GTG (V) / GTA (V)	13.945647		
GGG (G) / GGT (G)	13.981617		
ACC (T) / ACT (T)	13.991583		
GAC (D) / GAT (D)	14.214694		
CCG (P) / CCA (P)	15.127582		

codon (AA) substitution	ECM rate		
	synonymous	synonymous SER (S/Z)	nonsynonymous
AGG (R) / CGA (R)	15.287419		
CCA (P) / CCC (P)	15.426733		
CTG (L) / CTC (L)	15.484088		
CTC (L) / CTT (L)	15.874441		
TCA (S) / TCC (S)	15.982573		
TTC (F) / TTT (F)	16.011531		
ATA (I) / ATT (I)	16.415611		
CTA (L) / TTG (L)	16.560966		
TCG (S) / TCA (S)	17.424222		
AGA (R) / CGT (R)	17.450524		
AAC (N) / AAT (N)	17.634677		
CAG (Q) / CAA (Q)	17.923045		
AGG (R) / CGG (R)	18.531553		
TTG (L) / TTA (L)	18.541946		
GGG (G) / GGC (G)	18.675227		
GTG (V) / GTC (V)	18.744445		
GCG (A) / GCC (A)	19.373137		
ACG (T) / ACA (T)	19.459275		
GGA (G) / GGT (G)	21.171295		
CTA (L) / CTT (L)	21.437257		
CCC (P) / CCT (P)	23.496083		
TCC (S) / TCT (S)	23.655090		
TAC (Y) / TAT (Y)	24.177765		
GCA (A) / GCT (A)	24.400553		
GTA (V) / GTT (V)	25.313949		
GGG (G) / GGA (G)	25.640860		
CTA (L) / TTA (L)	27.219895		
CGC (R) / CGT (R)	27.244097		
ACG (T) / ACC (T)	28.470047		
AGC (Z) / AGT (Z)	28.579806		
CGG (R) / CGA (R)	30.574631		
ACA (T) / ACT (T)	31.915858		
AGA (R) / CGA (R)	31.949764		
CCG (P) / CCC (P)	33.453780		
TCG (S) / TCC (S)	35.359077		
CGG (R) / CGC (R)	35.563093		
TCA (S) / TCT (S)	35.921779		
CAC (H) / CAT (H)	38.685701		
CGA (R) / CGT (R)	39.595443		
CCA (P) / CCT (P)	40.922701		
AGG (R) / AGA (R)	43.916187		
TGC (C) / TGT (C)	56.838378		
