# Supplementary material for: Resolving Discrepancy between Nucleotides and Amino Acids in Deep-Level Arthropod Phylogenomics: Differentiating Serine Codons in 21-Amino-Acid Models
Source: PLoS One. 2012 Nov 20;7(11):e47450. doi: 10.1371/journal.pone.0047450 (PMC3502419; doi:10.1371/journal.pone.0047450)
Supplement: Table S5 — Number of Ser -containing alignment sites in relation to the number of taxa that encode Ser at those sites. (PDF) [file pone.0047450.s012.pdf]

Table S5: Number of SER-containing alignment sites in relation to the number of taxa that encode SER at those sites. Both non-co-SER sites (encoding exclusively S or Z) and co-SER sites (encoding S and Z) have numbers of taxa that encode S and/or Z, respectively, ranging from 2 to 80. The existence of non-co-SER sites with many taxa indicates that S and Z are not entirely freely interchangeable, i.e., that the different codon groups *Ser1* and *Ser2* are not truly synonymous. There is a total of 1,642 non-co- and 1,531 co-SER sites in the alignment, with about half of those non-co-SER sites (819) comprising only a single taxon that encodes S or Z.

# of taxa	non-co-SER	co-SER	# of taxa	non-co-SER	co-SER
1	819	n/a	41	1	7
2	310	144	42	0	3
3	137	132	43	0	3
4	78	99	44	0	9
5	65	101	45	1	9
6	25	63	46	2	8
7	17	63	47	1	11
8	19	48	48	0	8
9	12	45	49	2	9
10	12	37	50	4	8
11	10	37	51	3	15
12	12	22	52	0	9
13	8	24	53	1	10
14	4	22	54	1	17
15	4	14	55	2	5
16	0	23	56	4	11
17	1	10	57	5	11
18	3	12	58	8	16
19	4	12	59	1	16
20	2	12	60	0	12
21	0	18	61	0	13
22	0	16	62	3	9
23	0	13	63	0	23
24	1	5	64	3	13
25	0	14	65	2	20
26	0	10	66	1	18
27	0	7	67	0	24
28	0	9	68	5	12
29	2	6	69	9	17
30	1	6	70	3	8
31	2	14	71	2	10
32	1	5	72	4	21
33	1	6	73	2	12
34	2	6	74	4	15
35	1	6	75	3	14
36	4	5	76	3	6
37	1	3	77	0	2
38	0	9	78	0	2
39	0	4	79	1	1
40	1	9	80	2	3
