# Supplementary material for: Resolving Discrepancy between Nucleotides and Amino Acids in Deep-Level Arthropod Phylogenomics: Differentiating Serine Codons in 21-Amino-Acid Models
Source: PLoS One. 2012 Nov 20;7(11):e47450. doi: 10.1371/journal.pone.0047450 (PMC3502419; doi:10.1371/journal.pone.0047450)

Figure S1. Arthropod relationships and classification scheme based on *degen1* analysis of 75 ingroup plus five outgroup species [5].

Nodes that receive bootstrap support of at least 80% by one of four approaches [5] are arbitrarily numbered for convenient reference. Six groups that show major differences in their bootstrap values between *degen1* and *20AA* analyses are in boldface with filled circles identifying their nodes. Edafopoda is a newly proposed name (see text).

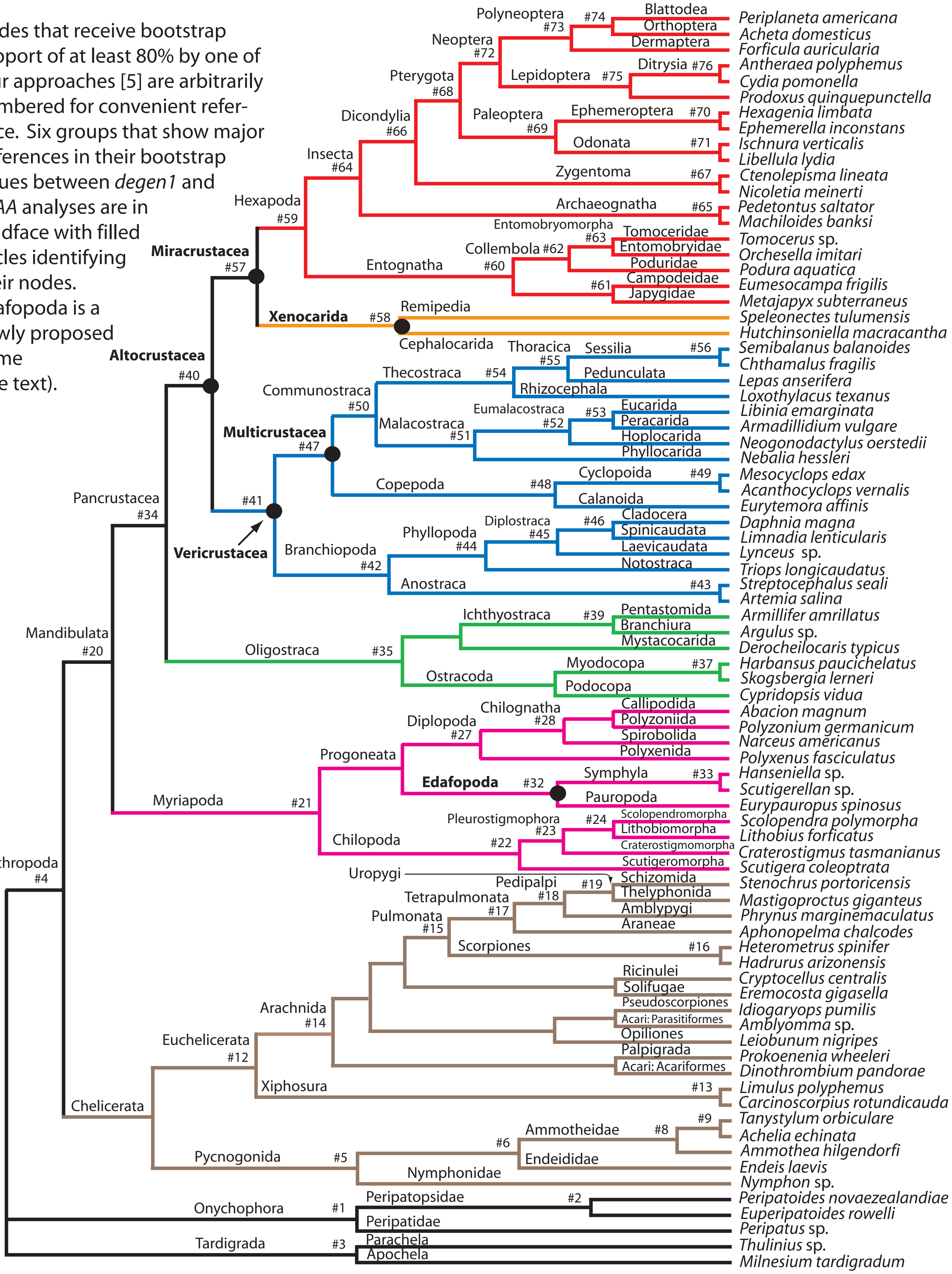

Supplement: Figure S1 — Arthropod relationships and classification scheme based on degen1 analysis of 75 ingroup plus five outgroup species [5] . (PDF) [file pone.0047450.s001.pdf]
