# Supplementary material for: Resolving Discrepancy between Nucleotides and Amino Acids in Deep-Level Arthropod Phylogenomics: Differentiating Serine Codons in 21-Amino-Acid Models
Source: PLoS One. 2012 Nov 20;7(11):e47450. doi: 10.1371/journal.pone.0047450 (PMC3502419; doi:10.1371/journal.pone.0047450)
Supplement: Figure S2 — Strict consensus of four maximum parsimony trees for 21AA data set plus bootstrap values (above branches) from 20AA (left) and 21AA (right) analyses. (PDF) [file pone.0047450.s002.pdf]

Figure S2. Strict consensus of four maximum parsimony trees for *21AA* data set plus bootstrap values (above branches) from *20AA* (left) and *21AA* (right) analyses.

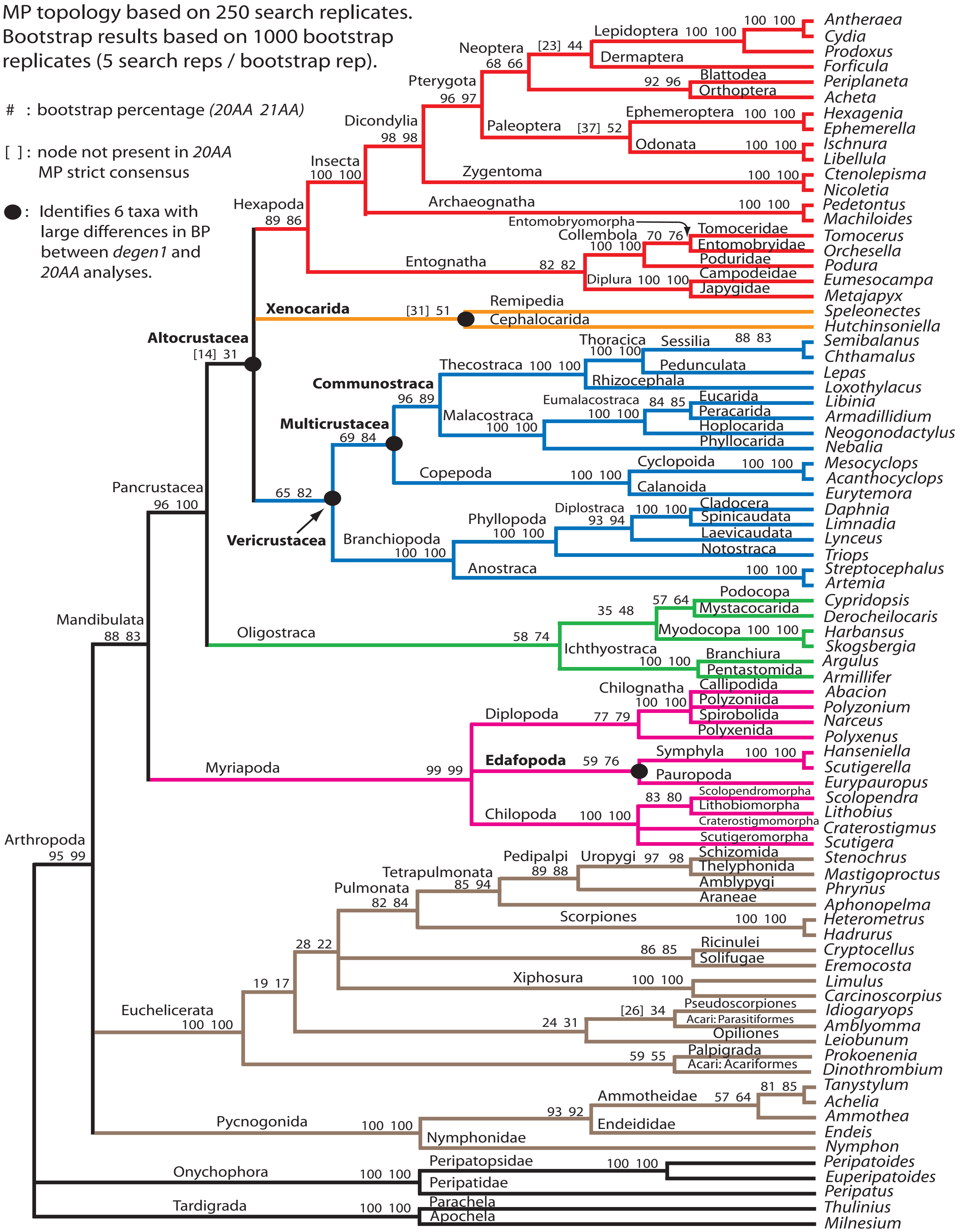

Commentary on Figure S2: Miracrustacea (BP, 24%), Progoneata (BP, 33%), and Chelicerata (BP, 57%) are all present in subsets of the four MP trees for the *21AA* analysis. Miracrustacea (BP, 12%) and Xenocarida (BP, 31%) are both present in subsets of the five MP trees for the *20AA* analysis, but not Altocrustacea (BP, 14%) and Chelicerata (40%). Progoneata (BP, 35%) is present in the strict consensus of the *20AA* analysis.
